# Costunolide and dehydrocostuslactone combination treatment inhibit breast cancer by inducing cell cycle arrest and apoptosis through c-Myc/p53 and AKT/14-3-3 pathway

**DOI:** 10.1038/srep41254

**Published:** 2017-01-24

**Authors:** Zhangxiao Peng, Yan Wang, Jianhui Fan, Xuejing Lin, Chunying Liu, Yang Xu, Weidan Ji, Chao Yan, Changqing Su

**Affiliations:** 1 Department of Molecular Oncology, Eastern Hepatobiliary Surgical Hospital & National Center of Liver Cancer, Second Military Medical University, Shanghai 200438, China; 2 School of Pharmacy, Shanghai Jiao Tong University, Shanghai 200240, China

## Abstract

Our previous studies demonstrated that volatile oil from *saussurea lappa* root (VOSL), rich in two natural sesquiterpene lactones, costunolide (Cos) and dehydrocostuslactone (Dehy), exerts better anti-breast cancer efficacy and lower side effects than Cos or Dehy alone *in vivo*, however, their anti-cancer molecular mechanisms were still unknown. In this study, we investigated the underlying mechanisms of Cos and Dehy combination treatment (CD) on breast cancer cells through proteomics technology coupled with Western blot validation. Ingenuity Pathways Analysis (IPA) results based on the differentially expressed proteins revealed that both VOSL and CD affect the 14-3-3-mediated signaling, c-Myc mediated apoptosis signaling and protein kinase A (PKA) signaling. Western blot coupled with cell cycle and apoptosis analysis validated the results of proteomics analysis. Cell cycle arrest and apoptosis were induced in a dose-dependent manner, and the expressions of p53 and p-14-3-3 were significantly up-regulated, whereas the expressions of c-Myc, p-AKT, p-BID were significantly down-regulated, furthermore, the ratio of BAX/BCL-2 were significantly increased in breast cancer cells after CD and VOSL treatment. The findings indicated that VOSL and CD could induce breast cancer cell cycle arrest and apoptosis through c-Myc/p53 and AKT/14-3-3 signaling pathways and may be novel effective candidates for breast cancer treatment.

Medicinal plants have long been used to treat various diseases including cancers for thousands of years. In contrast to the conventional cancer chemotherapy agents targeting single molecule, the mixture of phytochemicals is able to target multiple-molecules involved in the same pathway or several pathways responsible for cancer development, and exerts better therapeutic efficacy and lower side effects^1^. Dried 4- to 5-year-old roots of *Saussurea lappa*, known as Mu-xiang, are commonly used as medicine to treat breast cancer and breast hyperplasia in China, Japan and India^2^. Our previous study demonstrated that volatile oil from *Saussurea lappa* root (VOSL), sesquiterpene lactones-rich fraction, is responsible for the anti-breast cancer activity of Mu-xiang^3^. Gas chromatography-mass spectrometer (GC-MS) and liquid chromatography-mass spectrometer (LC-MS) analyses revealed that Costunolide (Cos) and Dehydrocostuslactone (Dehy), two natural sesquiterpene lactones, are the main ingredients of VOSL. Moreover, the combination treatment of Cos and Dehy (CD) showed synergistic anti-breast cancer efficiency both *in vitro* and *in vivo*^3^,^4^.

Much evidence indicates that the α,β-unsaturated carbonyl group in the α-methylene-γ-butyrolactone (Fig. 1) moiety of Cos and Dehy may play crucial roles through conjugation with SH-groups of target proteins to exert various biological activities, such as anti-inflammatory, anti-cancer, anti-virus, anti-oxidant, anti-diabetes, anti-ulcer, and anthelmintic activities, *etc*.^5^, of which, the anti-cancer activities and associated molecular mechanisms of Cos or Dehy have been reported in recent years, including inhibiting cancer cell proliferation^6^, accelerating apoptosis^7^, inducing cancer cell differentiation^8^, inhibiting metastasis and invasion^9^, reversing multidrug resistance^10^, restraining angiogenesis^11^. Our previous studies had demonstrated that VOSL has better anti-breast cancer efficacy and lower side effects than Cos or Dehy *in vivo*^3^, however, to the best of our knowledge, the synergistic anti-cancer molecular mechanism of Cos and Dehy (CD) in VOSL has not yet been studied.

Protein phosphorylation is a reversible protein post-translational modification which likes a molecular switch controlling important biological processes such as cell division, growth, differentiation, and death. Its misregulation is often associated with many human diseases, including cancer^12^. The research results from Choi *et al*. showed that sesquiterpene lactones can act as phosphatase inhibitors^13^. Therefore, we speculated that the cytotoxicity of VOSL or CD towards human breast cancer cells should be associated with protein phosphorylation pathways. Developments of phosphopeptide enrichment technologies along with improvements in mass spectrometer sensitivity, protein database and bioinformatics algorithms, have facilitated the qualitative and quantitative analyses of phosphopeptides from complex cell extracts and greatly revolutionized the fields of cell biology and cell signaling^14^. Currently, TiO_2_ has been considered as the most effective enrichment material for phosphopeptides^15^, and isobaric tags for relative and absolute quantification (iTRAQ) technology has been widely used to proteome research. In this study, we explored the anti-breast cancer molecular mechanism of VOSL and CD through TiO_2_-based enrichment of phosphopeptides and iTRAQ-based liquid chromatography and tandem mass spectrometry (LC-MS/MS) proteomics, coupled with Western blot validation.

## Results

### Identification of differentially expressed proteins and interaction networks analysis

Two sets isotope-labelled mixed samples (set one is Ctr (114) and Cos (117); set two is Ctr (114), Dehy (115), CD (116) and VOSL (117)) were analyzed by Nano LC–Q/TOF MS^E^ tandem mass spectrometry and identified a total of 430 proteins in set one (Supplementary Table S1), and 469 proteins in set two (Supplementary Table S2). Only protein quantification data with relative expression of >1.5 or <0.66 were chosen as differentially expressed proteins (Supplementary Table S3). The numbers of differentially expressed proteins in the Cos, Dehy, CD, and VOSL-treated group were 67, 59, 38, and 47, respectively. The differentially expressed proteins were imported into the IPA software for function annotation and interaction network analyses. The interaction networks of differentially expressed proteins in the Cos-treated group enriched 27 proteins (Supplementary Fig. S1A), thereinto, 15 proteins, which were APEX1, C1QBP, COL1A1, FAM162A, FXR1, HSPB8, HSPD1, LMNA, MAPT, NPM1, PGRMC1, RPS3, SET, SFN, STMN1, involved in the physiologic functions of cell death and survival. The interaction networks of differentially expressed proteins in the Dehy-treated group enriched 26 proteins (Supplementary Fig. S1B), of which there were 14 proteins, API5, BID, CFL1, EZR, FASN, GNB2L1, HMGB1, HSPB1, MAPT, SFN, SMARCB1, SON, TOP1 and YWHAZ, involved in the physiologic functions of cell death and survival. The interaction networks of differentially expressed proteins in the CD-treated group enriched 22 proteins (Supplementary Fig. S1C), among which there were 4 proteins, MAPT, CFL1, FLNB and SMARCA4, involved in the physiologic functions of cellular assembly, organization and cell cycle. The interaction networks in the VOSL-treated group enriched 23 differentially expressed proteins (Supplementary Fig. S1D), in which there were 11 proteins, API5, BID, GNB2L1, HSPB8, PARP1, RBM25, SFN, SND1, SON, TXN and UBR4, involved in the physiologic functions of cell death and survival.

### Common differentially expressed proteins

**Figure 2:**
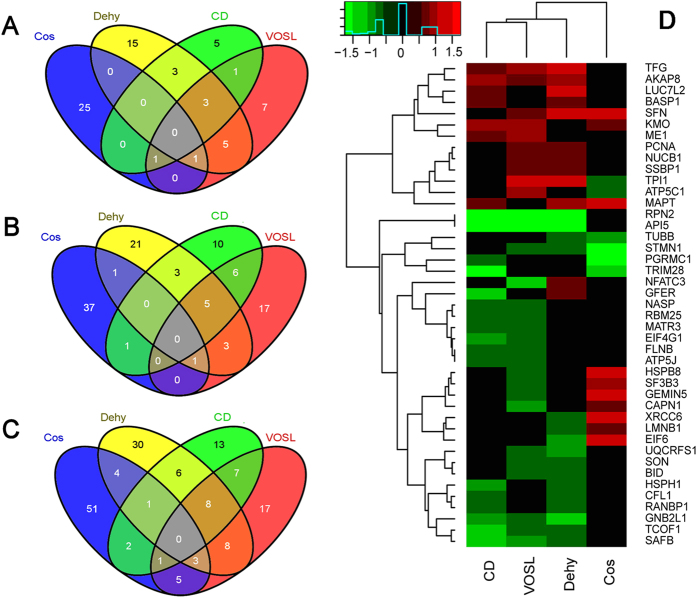
Venn diagram and heatmap of differentially expressed proteins. The results from proteomics revealed that (**A**) there are 14 common up-regulated proteins, (**B**) 20 common down-regulated proteins, and (**C**) 43 common differentially expressed proteins at the VOSL, CD, Dehy, and Cos-treated groups. Moreover, (**D**) the heatmap and cluster analysis revealed that the VOLS-treated group shared the most differentially expressed proteins with the CD-treated group, which further demonstrated that CD is the most important anti-breast cancer ingredients in VOSL and share the same pharmacological mechanisms with VOSL.

As we described above, Cos and Dehy are both natural sesquiterpene lactones, and they account for nearly 75% of VOSL by weight. Therefore, there should be some common differentially expressed proteins among the Cos, Dehy, CD and VOSL treated groups. Our results validated this speculation, 14 common up-regulated proteins, 20 common down-regulated proteins, and 43 common differentially expressed proteins were observed (Fig. 2A–C) and their alterations were depicted as a heatmap in Fig. 2D (use fold change, relative to Ctr). The results of cluster analysis revealed that the VOLS-treated group shared the most differentially expressed proteins with the CD-treated group, which further demonstrated that CD are the most important anti-breast cancer ingredients in VOSL and share the same pharmacological mechanisms with VOSL.

### Pathway analysis

**Figure 3:**
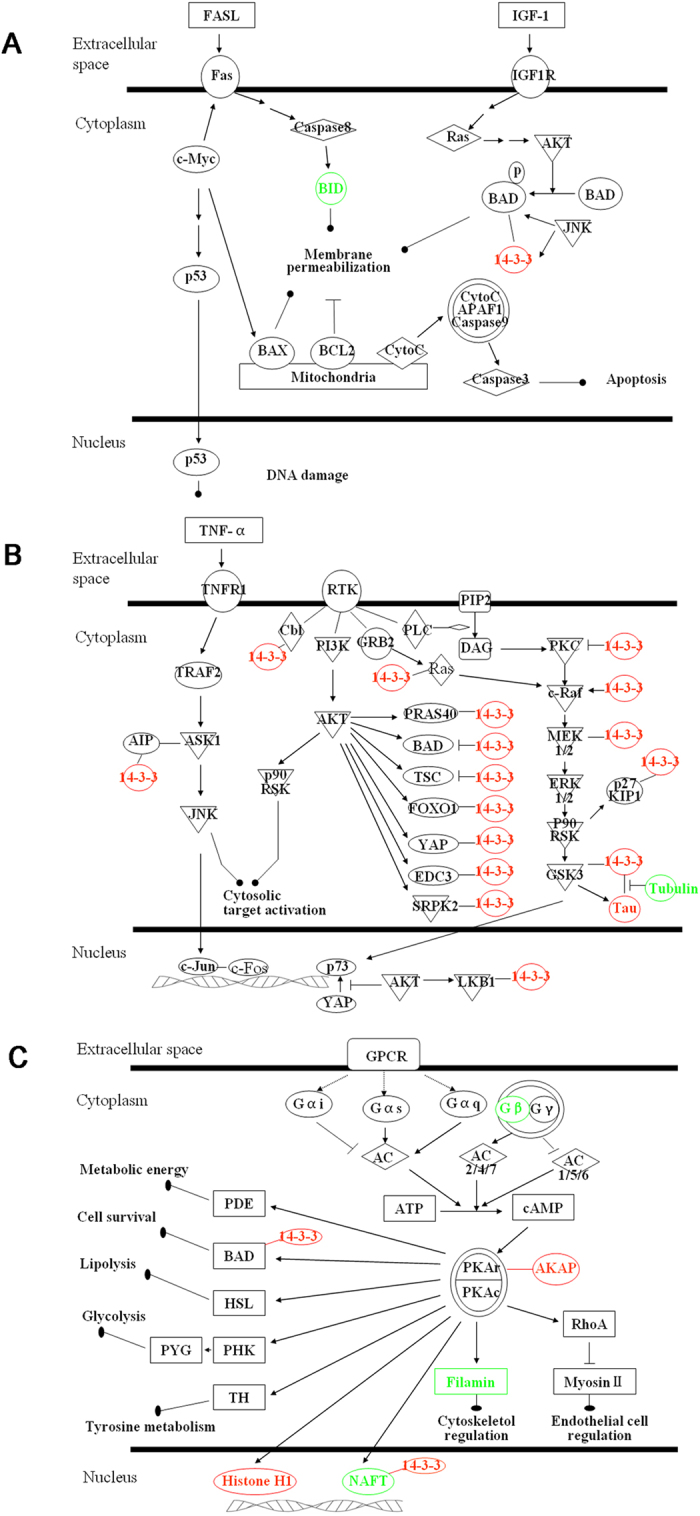
The signaling pathways affected by Cos, Dehy, CD or VOSL. (**A**) c-Myc mediated apoptosis signaling, (**B**) 14-3-3-mediated signaling, and (**C**) protein kinase A (PKA) signaling. Red and green colors represent up- and down- regulated, respectively.

The differentially expressed proteins at different treatment groups were imported into the IPA software for pathway analysis, the results demonstrated that the top pathway is c-Myc mediated apoptosis signaling and 14-3-3-mediated signaling for Dehy or Cos treatment, respectively (Fig. 3A and B), VOSL and CD shared the common top pathways, c-Myc mediated apoptosis signaling and protein kinase A (PKA) signaling (Fig. 3C).

### Western blot analysis and cAMP determination

**Figure 4:**
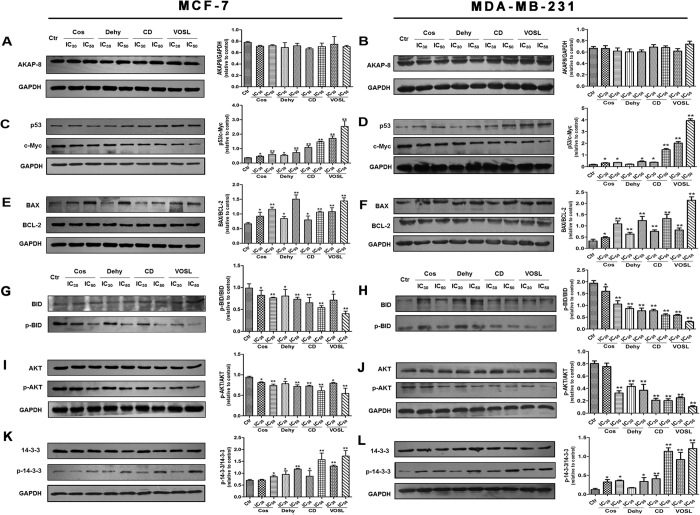
Cos, Dehy, CD and VOSL regulated c-Myc mediated apoptosis signaling and 14-3-3-mediated signaling pathways in breast cancer cells. MCF-7 cells (**A**,**C**,**E**,**G**,**I**,**K**) or MDA-MB-231 cells (**B**,**D**,**F**,**H**,**J**,**L**) were cultured in cell culture dishes, treated with 0 (Ctr), IC_30_ and IC_50_ of Cos, Dehy, CD, or VOSL, respectively for 48 h, then the expression of the indicated factors was examined by Western blot. Glyceraldehydes 3-phosphate dehydrogenase (GAPDH) was used as the loading control. The densitometry analysis of every factor was performed, and normalized with the corresponding GAPDH content. Values were presented as mean ± standard error (SE) of three independent experiments; *p < 0.05 and **p < 0.01 compared with the control group.

Pathway analysis based on the IPA software demonstrated that Cos, Dehy, CD or VOSL treatment affected some important signaling pathways in breast cancer cells, such as c-Myc mediated apoptosis signaling, 14-3-3-mediated signaling, and PKA signaling, therefore, Western blot analysis was used to validate these results. The results revealed that Cos, Dehy, CD and VOSL treatment all did not significantly regulated the expression of AKAP8 (Fig. 4A and B), however, CD and VOSL treatment can up-regulate the expression of p53 and down-regulate the expression of c-Myc significantly. The ratios of p53/c-Myc were increased dose-dependently in the four test groups compared with the control group, and the ratios of p53/c-Myc in the CD and VOSL treated groups were obviously bigger than those in the Cos or Dehy treated groups (Fig. 4C and D). p53 is a well-known tumor suppressor protein, its overexpression can induce up-regulation of Bax and mitochondria-dependent apopotosis^16^. In present study, we also found that the ratio of BAX/BCL-2 was increased (Fig. 4E and F) and the ratio of p-BID/BID (Fig. 4G and H) was decreased dose-dependently in the four test groups compared with the control group, which meant that Cos, Dehy, CD and VOSL all can induce MCF-7 and MDA-MB-231 cell apoptosis by the mitochondria-dependent intrinsic pathway.

Numerous studies showed that activation of AKT is positively correlated with cancer development, and c-Jun NH2-terminal kinase (JNK) can antagonize AKT-mediated survival signals by phosphorylating 14-3-3. In this study, the phosphorylation level of AKT was down-regulated (Fig. 4I and J) and the phosphorylation level of 14-3-3 was up-regulated (Fig. 4K and L) dose-dependently, with no obvious changes of the total AKT and 14-3-3 levels in the four test groups compared with the control group, and the ratios of p-AKT/AKT in the CD and VOSL treated groups were obviously lower than that in the Cos and Dehy treated groups, and the ratios of p-14-3-3/14-3-3 in the CD and VOSL treated groups were obviously higher than that in the Cos and Dehy treated groups.

**Figure 5:**
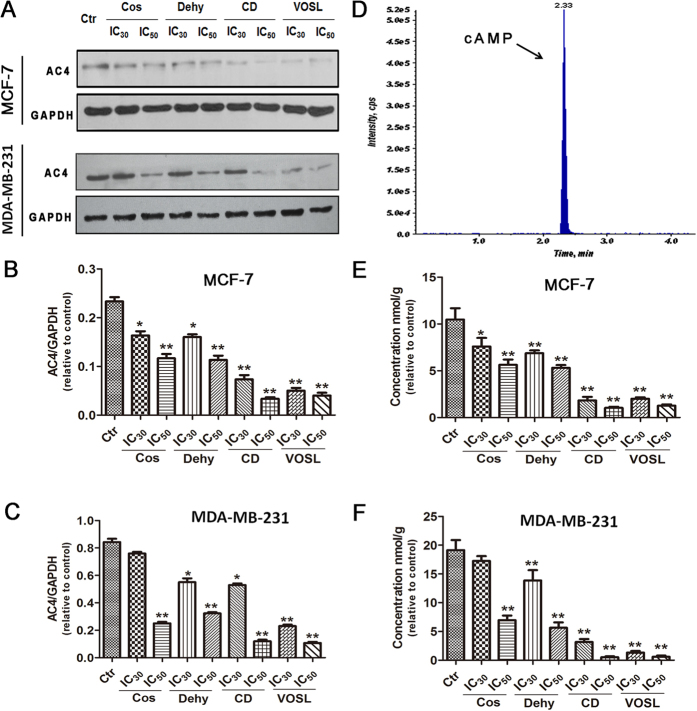
Expression levels of AC4 and cAMP in different treatment groups. MCF-7 cells or MDA-MB-231 cells were cultured in cell culture dishes, treated with 0 (Ctr), IC_30_ and IC_50_ of Cos, Dehy, CD, or VOSL, respectively, for 48 h, then the expression of AC4 was examined by Western blot (**A**). GAPDH was used as the loading control. The densitometry analysis of AC4 was performed, and normalized with GAPDH content (**B**,**C**). Quantification of intracellular cAMP was performed on a Waters Acquity UPLC system using a Waters Acquity BEH C18 column (2.1 × 50 mm^2^, 1.7 μm) coupled to an AB Sciex Triple Quad^TM^ 6500 mass spectrometer (**D**), and the concentrations of cAMP in different treatment groups were shown in (**E**,**F**). Values were presented as mean ± standard error (SE) of three independent experiments; *p < 0.05 and **p < 0.01 compared with the control group.

Adenyl cyclases (ACs) can convert adenosine triphosphate (ATP) into cyclic adenosine monophosphate (cAMP), which is necessary for the activation of PKA. The IPA analysis revealed that the signal pathway Gβγ/AC2/4/cAMP/PKA was inhibited by VOSL or CD. AC2 is distributed in brain and lung tissues, however, AC4 is widely distributed in various tissues. Therefore, we determined the levels of AC4 (Fig. 5A–C) and its catalysate cAMP (Fig. 5D–F). The results showed that the levels of AC4 and cAMP were decreased dose-dependently in the four test groups compared with the control group, and the levels of AC4 and cAMP in the CD and VOSL treated groups were obviously lower than those in the Cos and Dehy treated groups.

### Analyses of Cell cycle and apoptosis

**Figure 6:**
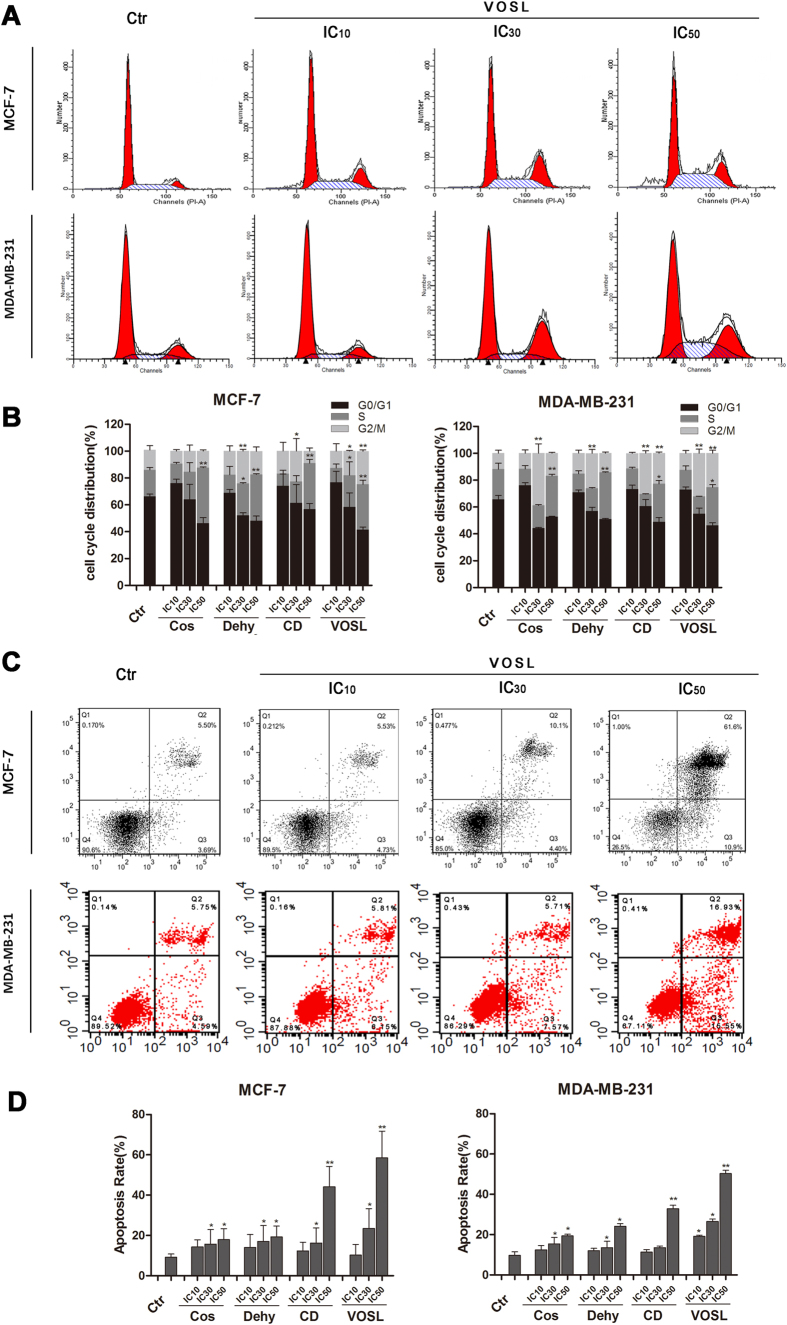
Effects on cell cycle and apoptosis of breast cancer cells after Cos, Dehy, CD, or VOSL treatment. MCF-7 cells or MDA-MB-231 cells were planted into 6-well plates at 3 × 10^5^ cells/well, incubated with the respective IC_10_, IC_30_, IC_50_ concentrations of Cos, Dehy, CD, or VOSL for 48 h, cells were harvested by trypsinisation, and then fixed by ice-cold ethanol (70%). After washing with PBS, the cell pellets were resuspended in propidium iodid (PI) staining buffer (50 μL/mL PI, RNase A). After 15 min of incubation at 37 °C, cell cycle distribution was analyzed by a FACScalibur System using ModFit software (**A**,**B**). MCF-7 or MDA-MB-231 cells were planted into 6-well plates at 2 × 10^5^ cells per well, treated with the respective IC_10_, IC_30_, IC_50_ concentrations of Cos, Dehy, CD, or VOSL for 48 h, stained with Annexin V-FITC/PI, and then detected by a FACScalibur system (**C**,**D**). The cell cycle distribution and apoptotic percentages from three independent experiments were analyzed and compared, *p < 0.05 and **p < 0.01 compared with the control group.

Western blot analysis demonstrated that Cos, Dehy, CD, and VOSL treatment all can increase the phosphorylation level of 14-3-3 protein in breast cancer cells. As 14-3-3 protein is a G_2_/M checkpoint regulator, which can regulate cell cycle progression and promote cell apoptosis^17^,^18^,^19^. Therefore, we thought Cos, Dehy, CD, and VOSL treatment should induce breast cancer cell cycle arrest. The results of cell cycle analysis were shown in Fig. 6A and B, which verified our speculation. IC_10_ of tested compound treatment did not change the progression of breast cancer cell cycle, interestingly, IC_30_ of Cos or Dehy treatment was apt to induce G_2_/M phase arrest, however IC_50_ of Cos or Dehy treatment was apt to induce S phase arrest. Moreover, IC_50_ of CD can significantly induce S phase arrest for MCF-7 cells and significantly induce S phase and G_2_/M phase arrest for MDA-MB-231 cells. IC_50_ of VOSL treatment can significantly induce S phase and G_2_/M phase arrest for the two breast cancer cell lines.

IPA analysis results revealed that apoptosis induction for MCF-7 cells is an important anti-cancer molecular mechanism of Cos, Dehy, CD, and VOSL. In this study, we further validated the IPA analysis results based on Annexin V-FITC/PI apoptosis analysis. The results of cell apoptosis analysis were shown in Fig. 6C and D. Cos, Dehy, CD and VOSL treatment all can dose-dependently induce MCF-7 cell and MDA-MB-231 cell apoptosis, moreover, the effects of apoptosis induction in the CD or VOSL-treated group are stronger than those in the Cos or Dehy-treated group.

### VOSL suppresses breast cancer xenograft growth

**Figure 7:**
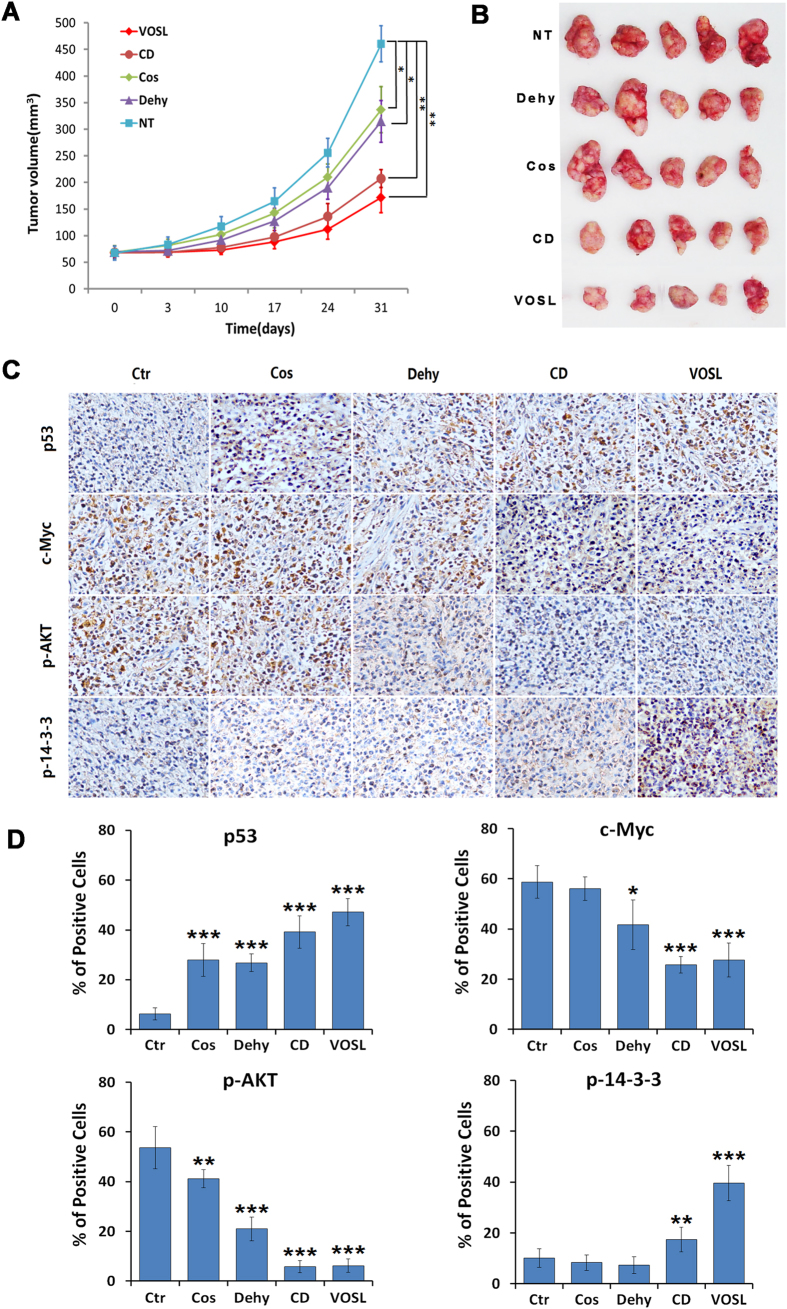
VOSL and its main active ingredients suppress the growth of breast cancer MDA-MB-231 xenografts. (**A**,**B**) The xenograft mouse models were randomly divided into five groups. The Cos, Dehy, CD and VOSL-treated groups were injected intraperitoneally at a dose of 20 mg/kg/day, respectively. The negative control (NT) was treated with an equal volume of vehicle. Tumor size was monitored at 0, 3, 10, 17, 24 and 31 days post-treatment and compared at 31 days post-treatment; *p < 0.05 and **p < 0.01 compared with the negative control (NT) group. (**C**,**D**) Tumor-bearing mice were sacrificed after 30 times of administrations and tumors were harvested and weighed, and then were cut into consecutive sections for examining the expression of p-AKT, p53, p-14-3-3 and c-Myc by immunohistochemistry. Original magnification 200×. The positive cells of the relevant factors in xenografts were presented as mean ± SD, *p < 0.05 and **p < 0.01 compared with the NT control group.

Our previous study demonstrated that VOSL and its main active ingredients can suppress the growth of estrogen receptor positive breast cancer MCF-7 xenografts^3^. In this study, we used the estrogen receptor negative breast cancer MDA-MB-231 xenograft model to evaluate further the anti-breast cancer efficiency of VOSL *in vivo*. The results revealed that VOSL and its main active ingredients also can suppress the growth of MDA-MB-231 xenografts, and VOSL and CD exhibited better anti-breast cancer activity than Cos or Dehy treatment alone (Fig. 7A and B). The inhibitory rates of VOSL, CD, Dehy, and Cos on MDA-MB-231 xenografts are 62.75%, 54.94%, 31.63%,and 27.85%, respectively, after intraperitoneal injections for 30 times. In addition, the expression of several key molecules, such as p53, c-Myc, p-AKT, p-14-3-3, in tumor tissues was determined by immunohistochemistry. The results demonstrated that the expression levels of c-Myc and p-AKT were all reduced and the expression levels of p53 and p-14-3-3 were all elevated in the treatment groups compared with the negative control group. Moreover, the CD or VOSL treated groups showed more obvious expression differences of these molecules than the Cos or Dehy treated groups (Fig. 7C and D). The results were consistent with the *in vitro* results. Therefore, we concluded that combination treatment of Cos and Dehy inhibits breast cancer through c-Myc/p53 and AKT/14-3-3 pathway.

## Discussion

Our previous researches revealed that Cos and Dehy in VOSL exhibited synergistic anti-breast cancer efficiency both *in vitro* and *in vivo.* In this study, we tried to investigate their molecular mechanisms. Increased proliferation capacities, uncontrolled cell cycle progression and apoptosis inhibition are the hallmark of cancer. Accordingly, the agents targeting one or more of these processes should be ideal cancer chemopreventive candidates^5^. Our research results demonstrated that VOSL, sesquiterpene lactones-rich fraction, can inhibit MCF-7 cell proliferation, induce cell cycle arrest and promote apoptosis through c-Myc/p53 signaling pathway and AKT/14-3-3 signaling pathway.

Much evidence showed that c-Myc plays a critical role in the control of cell proliferation, regulation of cell cycle, and serves as a link between proliferation and cell death by inducing p53-dependent apoptosis^20^,^21^. c-Myc has been documented to be both a positive and a negative signal for induction of apoptosis^22^. It is well known that overexpression of c-Myc induces normal cell apoptosis^23^. However, down-regulation of c-Myc expression may be mandatory for induction of apoptosis in many cancer cells, such as leukemia cells^24^, prostate cancer cells^25^, lung cancer cells^26^, and liver cancer cells^27^. c-Myc is frequently overexpressed in cancer cells^28^, enhanced expression of c-Myc will lead to activation of Cdk/Rb/E2F pathway, which is critical for cell cycle progression from G1 into S phase^20^. Moreover, c-Myc plays an important role in controlling various genes endcoding protein-synthesis components and regulating the expression of critical proteins in DNA replication machinery^29^,^30^. Therefore, its overexpression can activate the general apparatus for cellular metabolism and promote the process of DNA replication, so as to prepare cancer cell for continued proliferation. Conversely, deregulated c-Myc expression results in S phase arrest and cell apoptosis^20^. In addition, down-regulation of c-Myc expression can significantly decrease telomerase activity and inhibit growth of cancer cells^31^,^32^. Therefore, reduction of c-Myc expression has been considered as a potential therapeutic strategy for cancer^33^. In the present study, Cos, Dehy, CD and VOSL treatment all can reduce the expression of c-Myc, inhibit MCF-7 cell proliferation and induce its apoptosis.

The intrinsically dual nature of c-Myc function in growth and apoptosis and c-Myc-mediated apoptosis in normal cells requires wild-type p53^34^, however, the mechanisms of c-Myc-induciable apoptosis and how c-Myc and p53 involved in cancer cell apoptosis are not fully clarified. p53 is a well-known tumor suppressor protein, its overexpression can induce up-regulation of BAX and mitochondria-dependent apoptosis^35^. Our results were consistent with the references. Dehy, CD and VOSL treatment all can up-regulate the expression of p53, and increase the ratio of BAX to BCL-2. BAX and BCL-2 are both the BCL-2 family members, which serve as critical regulators of the mitochondrial-dependent apoptotic pathway. Thereinto, BCL-2 negatively regulates apoptosis and promotes cell survival, whereas BAX acts as a positive regulator of apoptosis to stimulate mitochondrial damage. The rise in ratio of BAX to BCL-2 will cause an opening of the mitochondrial permeability transition pore, which results in releasing pro-apoptotic proteins from the intermembrane space into the cytosol and triggering the mitochondrial-dependent apoptotic pathway^36^,^37^. Moreover, phosphorylation of 14-3-3 was dramatically increased and phosphorylation of BID was decreased in the VOSL-treated group. Phosphorylation of 14-3-3 will induce dephosphorylation of BAD. Dephosphorylated BAD and dephosphorylated BID were translocated to mitochondria, where they associate with Bcl-2/Bcl-x(L) to induce the mitochondrial-dependent apoptosis^38^. Taken together, CD and VOSL treatment can up-regulate the expression of p53, down-regulate the phosphorylation levels of BID and BAD and increase the ratio of BAX to BCL-2, to trigger the mitochondrial-dependent apoptotic pathway.

14-3-3 proteins are a family of evolutionary conserved modulator proteins, which regulate multiple signaling pathways involved in mitogenesis, cell cycle progression, and apoptosis in cells through binding to specific Ser/Thr-phosphorylated motifs on target proteins^39^. It has been considered as an integration point which integrates a variety of apoptotic and survival signals to adjudicate cell survival or death^38^. Mammals express seven 14-3-3 isoforms which can form homo and hetero dimers. Upon target binding, 14-3-3 proteins can affect the function of target protein by modulating the enzymatic activity of target protein, its protein stability, cellular localization or its association with other proteins. AKT is a central mediator of the PI3K/AKT pathway, its activation was positively correlated with cancer development^40^,^41^,^42^. Accumulating evidence showed that many AKT targets are also regulated by 14-3-3, including BAD^43^, TSC2^44^, p27Kip1^45^, YAP^46^, GSK3^47^, PRAS40^48^, and LKB1^49^. This sharing of targets is due to the overlap between the recognition motifs of AKT and 14-3-3: RxRxxS/T for AKT and RSxpS/TxP for 14-3-3^40^.

In our study, three isoforms of 14-3-3 proteins, 14-3-3σ (SFN), 14-3-3β (YWHAB) and 14-3-3ζ (YWHAZ), were enriched in 14-3-3-mediated signaling pathway by IPA analysis, and Western blot analysis demonstrated that the phosphorylation of 14-3-3 in MCF-7 cells and MDA-MB-231 cells was obviously increased after Cos, Dehy, CD or VOSL treatment. c-Jun NH2-terminal kinase (JNK) can antagonize AKT-mediated survival signals by phosphorylating 14-3-3. Research results from Choi *et al*. revealed that Cos treatment can activate JNK and induce apoptosis in Human Leukemia Cells^50^. Therefore, we proposed that Cos, Dehy, CD and VOSL treatment all can activate JNK and inactive AKT in breast cancer cells, and then the activated JNK will promote the phosphorylation of 14-3-3, which resulted in releasing the proapoptotic proteins, such as BAD and FOXO, and enhancing the activity of tumor suppressor, such as LKB1, to antagonize AKT-mediated survival signals, and finally to induce cancer cell apoptosis.

Protein kinase A (PKA) is a holoenzyme, which composes of two regulatory subunits (R) and two catalytic subunits (C). The two C-subunits are maintained in an inactive conformation by an R-subunit dimmer^51^. Elevated intracellular cAMP binds to the R-subunit of PKA causing phosphorylation of C-subunit of PKA, and then the activated C-subunit of PKA phosphorylates a range of substrate proteins on serine/threonine residues to govern many biological processes in cells^52^. The broad-substrate specificity of PKA is directed toward specific intracellular substrates by a multigene family of A-kinase anchor proteins (AKAPs), which target PKA to distinct subcellular loci and coordinate multiple signaling enzymes in supramolecular complexes^53^. In the present study, IPA analysis revealed that VOSL or CD treatment inhibits the PKA signaling pathway. The flux of cAMP is governed by two sets of enzymes: adenyl cyclase (AC) and phosphodiesterase (PDE), the former is activated by G-proteins to synthesize cAMP from ATP, and the later terminates cAMP signaling by hydrolyzing it to AMP^54^. Moreover, accumulating documents demonstrated that in addition to anchoring PKA many AKAPs contribute to the local degradation of cAMP by co-localizing PDEs^55^. Therefore, we proposed that CD or VOSL can act as a G protein-coupled receptor (GPCR) inhibitor to inhibit G-protein activity and decrease cAMP synthesis, and the decreased levels of AC4 and intracellular cAMP in the CD- and VOSL-treated groups supported this deduction. Meanwhile, CD or VOSL treatment might up-regulate the phosphorylation level of AKAP8 with no obvious changes of the total AKAP8, and increase the local degradation of cAMP by co-localizing PDE4A^56^,^57^. The decreased intracellular cAMP would cause inactivation of PKA and its downstream proteins, such as PDE, BAD, HSL, PHK, TH, NAFT, and Filamin, in turn affect the metabolic energy, lipolysis, glycolysis, tyrosine metabolism, and cytoskeletol regulation in cancer cells (Fig. 3C). These results are consistent with our previous report that is VOSL or CD treatment was able to attenuate the metabolic perturbation in energy metabolism, lipid metabolism, glycolysis, and tyrosine metabolism of MCF-7 xenograft mice^3^.

Taken together, VOSL contained multiple anti-cancer ingredients, at least Cos and Dehy, which targeted multiple signaling pathways, at least c-Myc/p53, AKT/14-3-3 and PKA signaling pathways to exhibit synergistic anti-breast cancer efficiency, and our previous study demonstrated that VOSL and CD shows better anti-breast cancer efficacy and lower side effects than Cos or Dehy alone *in vivo*^3^. Therefore, it is expected that VOSL and CD may serve as novel anti-tumor agents in prevention and treatment of breast cancer.

## Methods

### Reagents and antibodies

The annexin V-fluorescein isothiocyanate (FITC) Apoptosis Detection Kit was from MultiSciences Biotech (Shanghai, China). 4-Plex iTRAQ reagent was obtained from Applied Biosystems (Framingham, MA). Radio-Immunoprecipitation Assay (RIPA) lysis buffer, phenylmethanesulfonyl fluoride (PMSF), protease inhibitor cocktails and phosphatase inhibitor cocktails were purchased from Boster Biotech (Wuhan, China). Bradford Protein Assay Kit was purchased from Beyotime Biotech (Shanghai, China). Costunolide (Cos) and Dehydrocostus lactone (Dehy) (>98.0% purity) were purchased from Shanghai Yuanye Biotech (Shanghai, China). Nonphosphorylated peptides LY-6 (H-Leu-Thr-Arg-Pro-Arg-Tyr-OH), DE-11 (H-Asp-Ala-Glu- Phe-Arg-His-Asp-Ser-Gly-Tyr-Glu-OH), and phosphorylated peptides LY-6p (H-Leu-Thr-Arg -Pro-Arg-{pTyr}-OH), DE-11p (H-Asp-Ala-Glu-Phe-Arg-His-Asp-{pSer}-Gly-Tyr-Glu- OH) (>95.0% purity) were obtained from GL Biochem (Shanghai, China). cAMP (>98.0% purity) and other reagents used were purchased from Sigma-Aldrich (WI, USA).

Rabbit anti-human phospho-AKT (Thr308), rabbit anti-human total-AKT, rabbit anti-human p53, rabbit anti-human c-Myc, horseradish peroxidase-conjugated sheep anti-rabbit IgG antibodies were from Cell Signaling Technology (Cell Signaling Technology, Danvers, MA, USA). Mouse anti-human glyceraldehydes 3-phosphate dehydrogenase (GAPDH) was purchased from Bio-tech (Kangchen Bio-tech, Shanghai, China). Rabbit anti-human 14-3-3, rabbit anti-human phospho-14-3-3 β + ζ (Ser184 + Ser186), rabbit anti-human AKAP8, rabbit anti-human total-BID, rabbit anti-human phospho-BID (Ser61), rabbit anti-human BAX, rabbit anti-human BCL-2, rabbit anti-human adenyl cyclase 4 (AC4) were purchased from Abcam (Cambridge, UK).

### Experimental design

**Figure 8:**
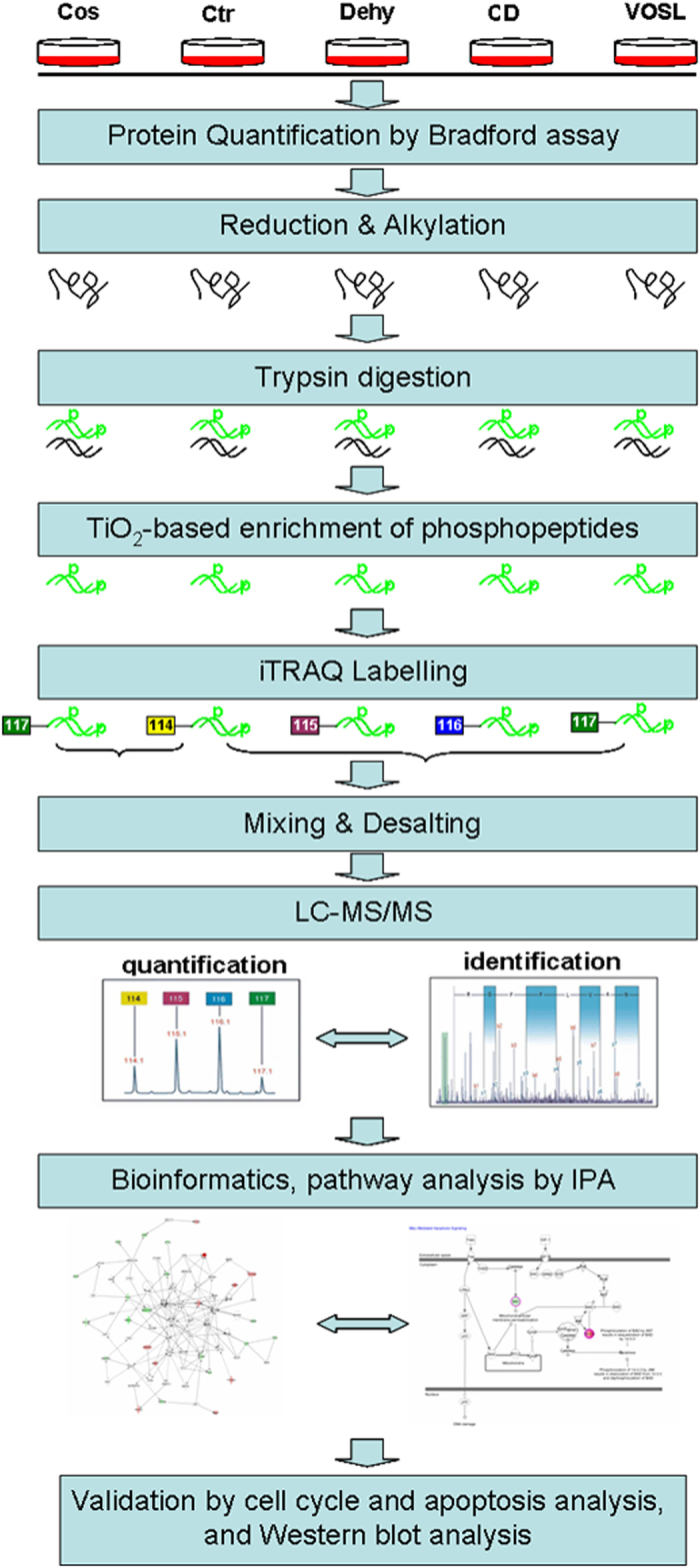
Workflow of this experiment. The proteins from five experimental groups including the control group (Ctr), Cos treated group, Dehy treated group, CD treated group, and VOSL treated group, were harvested and quantified. The quantified proteins were reduced and alkylated, and then digested into peptides. After TiO_2_-based enrichment, the phosphopeptides in each group were labeled by 4-plex iTRAQ reagent separately. The labeled phosphopeptides were mixed into two pools, and the resulting phosphopeptide pools were desalted and then analyzed by liquid chromatography- tandem mass spectrometry (LC-MS/MS) system. Identified differential expression proteins were further analyzed using Ingenuity Pathways Analysis (IPA) (version 9.0) (Ingenuity^®^ Systems, http://www.ingenuity.com) to statistically determine the functions and pathways most strongly associated with the protein list. Finally, the results of bioinformatics analysis were validated by cell cycle and apoptosis experiments, and Western blot experiments.

An illustration of the experimental workflow as well as the data analysis for this study is shown in Fig. 8. The proteins from five experimental groups, including the control group (Ctr), Cos treated group, Dehy treated group, CD (Cos/Dehy = 1/2, w/w; simulating the composition ratio of VOSL) treated group, and VOSL treated group, were harvested and quantified. The quantified proteins were reduced and alkylated, and then digested into peptides. After TiO_2_-based enrichment, the phosphopeptides in each group were labeled by 4-plex iTRAQ reagent separately. The labeled phosphopeptides were mixed into two pools, thereinto, one pool was consisted of Cos-treated group (labeled with m/z 117 isotope ion) and Ctr group (labeled with m/z 114 isotope ion), and the other pool was consisted of Ctr group (labeled with m/z 114 isotope ion), Dehy-treated group (labeled with m/z 115 isotope ion), CD treated group (labeled with m/z 116 isotope ion), and VOSL treated group (labeled with m/z 117 isotope ion). The resulting phosphopeptide pools were desalted and then injected into liquid chromatography- tandem mass spectrometry (LC-MS/MS) system. The phosphopeptides in each group were relatively quantified by reporter ions and identified based on sequence information from MS/MS. Identified differential expression proteins were further analyzed using Ingenuity Pathways Analysis (IPA) (version 9.0) (Ingenuity^®^ Systems, http://www.ingenuity.com) to statistically determine the functions and pathways most strongly associated with the protein list. Finally, the results of bioinformatics analysis were validated by cell cycle and apoptosis experiments, and Western blot experiments.

### Preparation of *Saussurea lappa* extracts

The extract of *Saussurea lappa* root was prepared as previously described^4^. Briefly, 10 g of *Saussurea lappa* roots were crushed into powder, and then was extracted with 100 mL hexane by sonication. The filtrates were evaporated to get VOSL. Analytic results from comprehensive two-dimensional gas chromatography- time-of-flight mass spectrometry (LECO Corporation, St Joseph, MI, USA) indicated that Cos and Dehy are the main ingredients of VOSL, accounting for nearly 72% of VOSL by weight (Supplementary Fig. S2 and Table S4). Commercial pure Cos, Dehy, or test sample of VOSL were dissolved in dimethyl sulfoxide (DMSO) to 10 mg/mL as a stock solution. According to the previous results of MCF-7 cell proliferation assays, 10%, 30% and 50% maximal inhibitory concentrations (IC_10_, IC_30_ and IC_50_) of Cos were calculated to be 0.9, 1.3 and 2.2 μg/mL, respectively. IC_10_, IC_30_ and IC_50_ of Dehy were 0.7, 1.1 and 1.7 μg/mL, respectively. IC_10_, IC_30_ and IC_50_ of CD were 0.4, 0.9 and 1.4 μg/mL, respectively. IC_10_, IC_30_ and IC_50_ of VOSL were 1.5, 2.4 and 3.3 μg/mL, respectively^4^. Moreover, according to the previous results of MDA-MB-231 cell proliferation assays, 10%, 30% and 50% maximal inhibitory concentrations (IC_10_, IC_30_ and IC_50_) of Cos were calculated to be 1.1, 2.3 and 4.2 μg/mL, respectively. IC_10_, IC_30_ and IC_50_ of Dehy were 0.8, 1.5 and 3.3 μg/mL, respectively. IC_10_, IC_30_ and IC_50_ of CD were 0.7, 1.3 and 2.8 μg/mL, respectively. IC_10_, IC_30_ and IC_50_ of VOSL were 1.3, 2.8 and 4.6 μg/mL, respectively.

### Cell culture and treatment

Human breast cancer MCF-7 and MDA-MB-231 cells were purchased from Chinese Academy of Sciences Cell Bank (Shanghai, China) on November 2015, which were authenticated and tested by the short tandem repeat (STR) method. Cells were cultured in high-glucose Dulbecco’s Modified Eagle’s Medium (DMEM) (Gibco, Gaithersburg, MD, USA) supplemented with 10% fatal bovine serum (FBS) (Hyclone, Thermo Scientific, UT, USA). Exponentially growing cultures were maintained in a humidified atmosphere of 5% CO_2_ at 37 °C.

MCF-7 cells were trypsinized and seeded into cell culture dishes (15 cm in diameter) at a density of 3 × 10^6^ cells per dish. The cells were cultured for 12 h to allow their adhesion to the dish, and then replaced with fresh medium containing IC_50_ of Cos, Dehy, CD, or VOSL, respectively. Whereas, the same volume of fresh medium without the test compounds was added to the cells as blank control. All culture dishes were then incubated for 48 h. Biological replicates for each group were performed in duplicate.

### Cell lysis and protein sample preparation

Cells were cultured for 48 h, the culture medium was aspirated, and the cells were rinsed 3 times with ice-cold phosphate buffer solution (PBS). The cells were harvested and softly homogenized in an ice-cold RIPA Lysis Reagent (BOSTER, Wuhan, China) containing 1% protease inhibitor cocktail and phosphatase inhibitor cocktail (BOSTER, Wuhan, China), sonicated for 400 W × 4 min (6 s on, 4 s off), and centrifuged at 20,000 × *g* for 15 min. The supernatant containing the total MCF-7 cell proteins was precipitated with 5 volumes of ice-cold ethanol/ acetone/acetic acid (50/50/0.1, v/v/v) at −20 °C for 2 h. Protein precipitant was centrifuged at 20,000 × *g* for 1 h. The pellet was washed separately with 1 ml of acetone and 75% ethanol, and redissolved in 600 μL of 6 M guanidine hydrochloride, 100 mM ammonium bicarbonate solution, the protein concentration was determined by Bradford Protein Assay Kit (BOSTER, Wuhan, China) according to the manufacturer’s instructions. The protein concentration of each sample was diluted to 3.00 mg/mL with 6 M guanidine hydrochloride, 100 mM ammonium bicarbonate solution. Cysteine bonds were reduced with 20 mM DTT (dithiothreitol) (Sigma-Aldrich) for 1 h at 56 °C and alkylated with 60 mM IAA (iodoacetamide) (Sigma-Aldrich) for 40 min at room temperature in darkness, and the resulting mixed solution was transferred into ultrafiltration units (10 K, PN: VN01H02, Sartorious). After 30 min of centrifugation at 10,000 × *g*, the protein adhered to the filter membrane was rinsed 3 times with 0.5 M urea, 100 mM ammonium bicarbonate solution and incubated overnight with trypsin (enzyme-to-protein ratio = 1:50, w/w) in 0.5 M urea, 100 mM ammonium bicarbonate solution at 37 °C. The peptide samples in ultrafiltration units were collected by centrifugation at 10,000 × *g* for 30 min, and acidified to 1% formic acid. The resulting samples were transferred to 1.5 mL EP tubes for vacuum-dry at 25 °C, the residues were redissolved in loading buffer (65% acetonitrile/2% trifluoroacetic acid/ saturated by glutamic acid) for further TiO_2_ beads enrichment.

### TiO_2_ enrichment and iTRAQ labeling

The commercial TiO_2_ beads (GL Sciences, Tokyo, Japan) were used to enrich phosphorylated peptides according to the manufacturer’s instructions. Briefly, 200 μL of loading buffer (65% acetonitrile/ 2% trifluoroacetic acid/ saturated by glutamic acid) was used to equilibrium of TiO_2_ beads, and then 200 μL of peptide samples in loading buffer were incubated with TiO_2_ beads (peptides/beads = 1/4, w/w) for 20 min at room temperature. For consecutive incubations, the peptide-beads slurry was incubated and centrifuged, then the supernatant was incubated with another aliquot of freshly prepared TiO_2_ beads for the next enrichment. The enrichment procedures were repeated two times. The incubated beads were then washed with 800 μL of wash buffer I (65% acetonitrile/0.5% trifluoroacetic acid) and buffer II (65% acetonitrile/ 0.1% trifluoroacetic acid). and the bound peptides were eluted once with the 200 μL elution buffer I (300 mM aqua ammonia/50% acetonitrile) and twice with 200 μL elution buffer II (500 mM aqua ammonia/60% acetonitrile). All the incubation, washing as well as elution procedure, was rotated end-over-end for 20 min at room temperature. The eluates were dried by a vacuum dryer (RE-52AA, Shanghai Zhenjie Instrument Co. Ltd) to obtain the phosphorylated peptides. The enrichment efficiency of commercial TiO_2_ beads for phosphopeptide was evaluated and the results were shown in Supplementary Figs S3 and S4 and Table S5.

The iTRAQ labeling was carried out using an iTRAQ Reagent 4-Plex kit (Applied Biosystems, USA) according to the manufacturer’s protocol. Two sets isotope-labelled samples (one set is Ctr (m/z 114) and Cos (m/z 117); the other is Ctr (m/z 114), Dehy (m/z 115), CD (m/z 116) and VOSL (m/z 117)) were mixed and dried by a vacuum dryer, respectively. The resulting samples were redissolved in 0.1% formic acid (FA), and then were desalted on a C_18_ ZipTip (P10, Millipore, Billerica) prior to analysis.

### Mass spectrometric analysis

Dionex ultimate 3000 nano liquid chromatography system (Thermofisher Dionex) was used for online reversed phase chromatographic separation of the phosphopeptide samples prior to nanoelectrospray ion source and mass spectrometric detection. 5 μL of peptide samples (dissolved in 98% H_2_O/ 2% acetonitrile / 0.1% formic acid) were loaded onto a C_18_ trap column (0.1 mm × 2 cm, 5 μm, Thermofisher Dionex) using a flow rate of 10 μL/min. The oven temperature was set at 50 °C. Separation of the peptides was performed on a C_18_ analytical column (75 μm × 150 mm, 3 μm, Thermofisher Dionex) with a flow rate of 400 nL/min. The mobile phase of the binary gradient elution consisted of (A) water with 0.1% formic acid and (B) acetonitrile with 0.1% formic acid, and separation was performed using the following gradient: 2–8% B over 0–2 min, 8–20% B over 2–75 min, 20–35% B over 75–93 min, 35–80% B over 93–98 min, the composition was held at 80% B for 10 min. All the samples were kept at 4 °C during analysis. The mass spectrometric (MS) data were collected using a maxis impact Q-TOF (Bruker) equipped with an electrospray ionization (ESI) source operating in nanospray positive ion mode. The mass spectrometer was set as follows: Scan mode, full-scan MS and MS/MS scan; MS spectra rate, 3 HZ; MS/MS spectra rate, 5~10 HZ; Acquisition modes, data dependent analysis; Mass and MS/MS range, 50~2500 amu; lockmass, 445 Da; Precursor ion list, 350~1500 m/z; Source Capillary, 1800–1900 v; Dry gas, 2.0 L/min; Dry temperature, 120 °C.

### Protein identification, quantification and bioinformatic analysis

The spectral data were imported to “Compass 4.1” software to generate the “.mgf” files, which were subsequently submitted to “Mascot 2.4” software (Matrix Science, London, UK) for protein identification. Database searches of each file were performed using the SwissProt (2013–6) *homo sapiens* species specific database with the following parameters: peptide tolerance, 10–30 ppm; MS/MS tolerance, 0.05 Da; Quantitation, itraq 4plex; Enzyme, typsin; Max missed cleavages, 2; Fixed modification, carboxymethyl, methylthio of cysteine, iTRAQ 4plex of lysine, and iTRAQ 4plex of the n-terminus; Variable modification, oxidation, phospho of serine, threonine and tyrosine, and iTRAQ 4plex of tyrosine. The identification results of peptides were acceptable when the similarity was >96% and the false discovery rate (FDR) was <1%. The resulting Mascot result files (*.dat) were loaded into the Scaffold Q + S 4.2.0 software (Proteome Software Inc., Portland, OR) for further processing. For relative quantitation, only peptides unique for a given protein were considered, thus excluding those common to other isoforms or proteins of the same family. Proteins were identified on the basis of having at least one peptide with an ion score above 99% confidence. Identified peptides that are unique to a specific protein were used to determine relative quantitation of a protein between the four samples or the two samples, and the intensities of the iTRAQ^®^ reporter ion m/z values (114, 115, 116 and 117) were used to estimate the relative abundances of a particular peptide. For proteins with more than one qualified peptide matches, multiple average peak area ratios were calculated using the peak area ratios of the peptides originating from the same protein^58^. In the present study, only protein quantification data with relative expression of >1.5 or <0.66 were chosen as differentially expressed proteins, which were analyzed by IPA software version 9.0 (Ingenuity^®^ Systems, California, USA; http://www.ingenuity.com). The functional analysis identified the top pathways and biological functions that were most significant to the data set.

### Western blot analysis

Cells were seeded into cell culture dishes (10 cm in diameter), at a density of 1 × 10^6^ cells per dish, and were cultured for 12 h to allow their adhesion to the dish, and then replaced with fresh medium containing two concentration levels, IC_30_ and IC_50_, of Cos, Dehy, CD, or VOSL, respectively, whereas the same volume of fresh medium without test compounds were added to the cells as blank control. All culture dishes were incubated for 48 h, and then cells were harvested, resuspended in RIPA lysis buffer, containing 1% protease inhibitor cocktail and phosphatase inhibitor cocktail, and centrifuged at 13,000 rpm for 30 min at 4 °C. Protein concentration was determined by bicinchoninic acid (BCA) method, using bovine serum albumin as standard. Proteins were separated on 10% sodium dodecyl sulfate-polyacrylamide gel (SDS-PAGE) and transferred onto polyvinylidene fluoride (PVDF) membrane. The membranes were blocked in defatted milk (5% in Tris-buffered saline with Tween-20 buffer) at 37 °C for 1 h, and then were incubated with various primary antibodies overnight at 4 °C. Finally, primary antibodies were revealed using horseradish peroxidase-conjugated anti-rabbit antibodies and an ECL chemiluminescence detection system (Pierce). The bands were semi-quantified using Image J software.

### cAMP determination

Cells were seeded into cell culture dishes (10 cm in diameter), at a density of 1 × 10^6^ cells per dish, and cultured for 12 h to allow their adhesion to the dish, and then replaced with fresh medium containing two concentration levels, IC_30_ and IC_50_, of Cos, Dehy, CD, or VOSL, respectively, whereas, the same volume of fresh medium without test compounds were added to the cells as a blank control. After incubation for 48 h, cell culture medium was removed and the adherent cells were washed twice with PBS quickly, and then the metabolic activity of adherent cells were quenched with liquid nitrogen, and the intracellular cAMP was extracted and determined as Shao *et al*.^59^ with a few modifications. Briefly, 1.0 mL of cold methanol-water (v/v = 4/1) containing 0.1% formic acid was added into the culture dish, and then the adherent cells were scraped, collected and transferred to 2 mL centrifuged tubes, and then ultrasonicated for 3 min (4 s off, 6 s on) in ice bath. After that, the resulting samples were centrifuged at 4 °C for 10 min at 15000 g, and 0.6 mL supernatant was transferred into a 1.5 mL centrifuged tubes, and then dried using nitrogen stream. The residues were resolved with 200 μL acetonitrile-water (v/v = 1/4), and centrifuged at 4 °C for 10 min at 15000 g, 150 μL supernatant was transferred into a glass auto-sampler for LC-MS/MS analysis.

Quantification of cAMP was performed on a Waters Acquity UPLC system (Waters^®^ Corporation, MA, USA) using a Waters Acquity BEH C18 column (2.1 × 50 mm^2^, 1.7 μm) coupled to an AB Sciex Triple Quad^TM^ 6500 mass spectrometer (Applied Biosystems Corporation, MA, USA). The binary gradient elution system consisted of (A) water (containing 0.1% formic acid, v/v) and (B) acetonitrile (containing 0.1% formic acid, v/v) and separation on BEH C18 column (2.1 × 50 mm^2^, 1.7 μm) was achieved under the following gradient: 5–5% B over 0–0.2 min, 5–35% B over 0.2–3.5 min, 35–80% B over 3.5–6 min, 80–100% B over 6–6.5 min, 100–100% B over 6.5–8 min. The flow rate was 0.4 mL/min. The mass spectrometric data were collected using positive ion mode. The source temperature was set at 120 °C with a cone gas flow of 40 L/h, a desolvation gas temperature of 400 °C with a gas flow of 650 L/h, the capillary voltage was set to 5500 V. Collision energy (CE) and declustering potential (DP) were optimized for each standard manually. The external standard method was used for quantification of cAMP, and the contents of cAMP in different groups were adjusted by the total protein concentrations in different groups.

### Cell cycle analysis

Cell cycle was analyzed according to Lohberger *et al*. report^60^. Briefly, after incubation with the respective IC_10_, IC_30_, IC_50_ concentrations of Cos, Dehy, CD, or VOSL for 48, cells were harvested by trypsinisation, and then were fixed by ice-cold ethanol (70%) for 10 min at 4 °C. After washing with PBS, the cell pellets were resuspended in propidium iodid (PI) staining buffer (50 μL/mL PI, RNAse A; Beckman Coulter). After 15 min of incubation at 37 °C, cell cycle distribution was analyzed by a FACScalibur System (BD Biosciences) using ModFit software.

### Annexin V-FITC/PI apoptosis analysis

Annexin V-FITC/PI double staining was employed to quantify the apoptotic rate of MCF-7 cells. Briefly, cells were plated into 6-well plates at 2 × 10^5^ cells per well and cultured for 12 h to allow their adhesion to the dish. After incubation with the respective IC_10_, IC_30_, IC_50_ concentrations of Cos, Dehy, CD, or VOSL for 48 h, cells were harvested by trypsinisation, and then were stained with annexin V-FITC/PI double fluorescence apoptosis detection kit (BD Biosciences) following the manufacturer’s instruction. After incubation for 15 min at room temperature in darkness, the apoptotic cells were detected using a FACScalibur System (BD Biosciences). Compensation was performed by single annexin and PI measurements and data were analyzed by FCS3 express software (De Novo software). Untreated cells were used as a negative control.

### *In vivo* anti-xenograft study

A total of thirty healthy female BALB/c nude mice (4 weeks old) were provided by Shanghai SLAC Laboratory Animal Center of Chinese Academy of Sciences (Shanghai, China), and maintained in specific pathogen-free (SPF) conditions. MDA-MB-231 cells were injected subcutaneously into the right armpit of the nude mice at 4 × 10^6^ cells in 150 μl PBS. After 15 days incubation, three mice with the largest tumors and two mice with the smallest tumors were excluded, and the other twenty-five xenograft mice with tumor size reaching a diameter of about 5 mm were randomly divided into five groups. The Cos, Dehy, CD and VOSL-treated groups were injected intraperitoneally with the corresponding agents at a dose of 20 mg/kg/day, respectively. The negative control (NT) was treated with an equal volume of vehicle. Tumor size was monitored at 0, 3, 10, 17, 24 and 31 days post-treatment, and tumor volume was calculated using the formula: “maximum diameter × minimum diameter^2^/2. Tumor-bearing mice were sacrificed after 30 times of administrations. Tumors were harvested and weighed. The tissues were cut into consecutive sections for examining the expression of p-AKT, p53, p-14-3-3 and c-Myc by immunohistochemistry. All procedures involving animals were reviewed and approved by the Experimental Animal Ethics Committee of Second Military Medical University, and we confirm that all experiments were performed in accordance with relevant guidelines and regulations.

### Statistical analysis

All values are expressed as mean values ± standard error (SE). Student’s unpaired *t*-test was used to evaluate differences between treated groups and their respective controls. The significance of dose responses was evaluated by repeated measures analysis. IC_10_, IC_30_ and IC_50_ values were calculated using the PASW Statistics 18.0.

## Additional Information

**How to cite this article**: Peng, Z. *et al*. Costunolide and dehydrocostuslactone combination treatment inhibit breast cancer by inducing cell cycle arrest and apoptosis through c-Myc/p53 and AKT/14-3-3 pathway. *Sci. Rep.* **7**, 41254; doi: 10.1038/srep41254 (2017).

**Publisher's note:** Springer Nature remains neutral with regard to jurisdictional claims in published maps and institutional affiliations.

## Supplementary Material

Supplementary Information

## Figures and Tables

**Figure 1:**
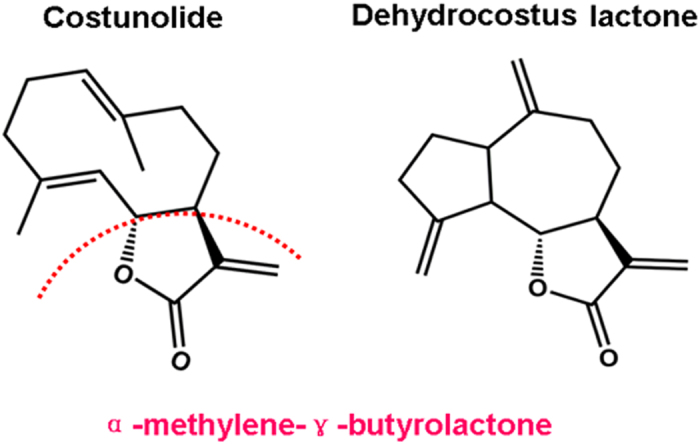
Chemical structures of Cos (C_15_H_20_O_2_) and Dehy (C_15_H_18_O_2_). The α,β-unsaturated carbonyl group in the α-methylene-γ-butyrolactone moiety of Cos and Dehy is very important for exerting their various biological activities, such as anti-inflammatory, anti-cancer, anti-virus, anti-oxidant, anti-diabetes, anti-ulcer, and anthelmintic activities.
