# Supplementary material for: Costunolide and dehydrocostuslactone combination treatment inhibit breast cancer by inducing cell cycle arrest and apoptosis through c-Myc/p53 and AKT/14-3-3 pathway
Source: Sci Rep. 2017 Jan 24;7:41254. doi: 10.1038/srep41254 (PMC5259746; doi:10.1038/srep41254)
Supplement: Supplementary Information [file srep41254-s1.pdf]

Supplementary Information

Costunolide and dehydrocostuslactone combination treatment inhibit breast cancer by inducing cell cycle arrest and apoptosis through c-Myc/p53 and AKT/14-3-3 pathway

Zhangxiao Peng[1,2,*], Yan Wang[2,*], Jianhui Fan[1,*], Xuejing Lin[1], Chunying Liu[1], Yang Xu[1], Weidan Ji[1], Chao Yan[2]& Changqing Su[1]

[1] Department of Molecular Oncology, Eastern Hepatobiliary Surgical Hospital & National Center of Liver Cancer, Second Military Medical University, Shanghai 200438, China

[2] School of Pharmacy, Shanghai Jiao Tong University, Shanghai 200240, China

* These authors contributed equally to this work.

Correspondence and requests for materials should be addressed to C. Su (email: suchangqing@gmail.com) or C. Yan. (email: chaoyan@unimicrotech.com)

Table S1 Identified a total of 430 proteins in set one

#	Identified Proteins	Accession Number	Molecular Weight	Probability
1	Neuroblast differentiation-associated protein AHNAK OS=Homo sapiens GN=AHNAK PE=1 SV=2	AHNK_HUMAN	629 kDa	100%
2	Keratin, type II cytoskeletal 8 OS=Homo sapiens GN=KRT8 PE=1 SV=7	K2C8_HUMAN	54 kDa	100%
3	Myosin-9 OS=Homo sapiens GN=MYH9 PE=1 SV=4	MYH9_HUMAN	227 kDa	100%
4	Cluster of Tubulin beta chain OS=Homo sapiens GN=TUBB PE=1 SV=2 (TBB5_HUMAN)	TBB5_HUMAN [2]	50 kDa	100%
4.1	Tubulin beta chain OS=Homo sapiens GN=TUBB PE=1 SV=2	TBB5_HUMAN	50 kDa	100%
4.2	Tubulin beta-4B chain OS=Homo sapiens GN=TUBB4B PE=1 SV=1	TBB4B_HUMAN	50 kDa	100%
5	Tubulin alpha-1A chain OS=Homo sapiens GN=TUBA1A PE=1 SV=1	TBA1A_HUMAN (+2)	50 kDa	100%
6	Transcription intermediary factor 1-beta OS=Homo sapiens GN=TRIM28 PE=1 SV=5	TIF1B_HUMAN	89 kDa	100%
7	Nucleolin OS=Homo sapiens GN=NCL PE=1 SV=3	NUCL_HUMAN	77 kDa	100%
8	Heat shock 70 kDa protein 4 OS=Homo sapiens GN=HSPA4 PE=1 SV=4	HSP74_HUMAN	94 kDa	98%
9	Cluster of Filamin-A OS=Homo sapiens GN=FLNA PE=1 SV=4 (FLNA_HUMAN)	FLNA_HUMAN [3]	281 kDa	100%
9.1	Filamin-A OS=Homo sapiens GN=FLNA PE=1 SV=4	FLNA_HUMAN	281 kDa	100%
9.2	Filamin-B OS=Homo sapiens GN=FLNB PE=1 SV=2	FLNB_HUMAN	278 kDa	99%
9.3	Filamin-C OS=Homo sapiens GN=FLNC PE=1 SV=3	FLNC_HUMAN	291 kDa	76%
10	Cluster of Tropomyosin alpha-4 chain OS=Homo sapiens GN=TPM4 PE=1 SV=3 (TPM4_HUMAN)	TPM4_HUMAN [3]	29 kDa	100%
10.1	Tropomyosin alpha-4 chain OS=Homo sapiens GN=TPM4 PE=1 SV=3	TPM4_HUMAN	29 kDa	100%
10.2	Tropomyosin alpha-3 chain OS=Homo sapiens GN=TPM3 PE=1 SV=1	TPM3_HUMAN	33 kDa	84%
11	Brain acid soluble protein 1 OS=Homo sapiens GN=BASP1 PE=1 SV=2	BASP1_HUMAN	23 kDa	99%

12	Keratin, type I cytoskeletal 18 OS=Homo sapiens GN=KRT18 PE=1 SV=2	K1C18_HUMAN	48 kDa	100%
13	Fructose-bisphosphate aldolase C OS=Homo sapiens GN=ALDOC PE=1 SV=2	ALDOC_HUMAN	39 kDa	99%
14	Profilin-1 OS=Homo sapiens GN=PFN1 PE=1 SV=2	PROF1_HUMAN	15 kDa	99%
15	Pyruvate kinase isozymes M1/M2 OS=Homo sapiens GN=PKM PE=1 SV=4	KPYM_HUMAN	58 kDa	100%
16	Alpha-enolase OS=Homo sapiens GN=ENO1 PE=1 SV=2	ENOA_HUMAN	47 kDa	100%
17	Cluster of Actin, cytoplasmic 1 OS=Homo sapiens GN=ACTB PE=1 SV=1 (ACTB_HUMAN)	ACTB_HUMAN [6]	42 kDa	100%
17.1	Actin, cytoplasmic 1 OS=Homo sapiens GN=ACTB PE=1 SV=1	ACTB_HUMAN (+1)	42 kDa	81%
17.2	Actin, aortic smooth muscle OS=Homo sapiens GN=ACTA2 PE=1 SV=1	ACTA_HUMAN (+3)	42 kDa	79%
18	60S ribosomal protein L4 OS=Homo sapiens GN=RPL4 PE=1 SV=5	RL4_HUMAN	48 kDa	100%
19	Ubiquitin-60S ribosomal protein L40 OS=Homo sapiens GN=UBA52 PE=1 SV=2	RL40_HUMAN (+3)	15 kDa	99%
20	78 kDa glucose-regulated protein OS=Homo sapiens GN=HSPA5 PE=1 SV=2	GRP78_HUMAN	72 kDa	100%
21	14-3-3 protein zeta/delta OS=Homo sapiens GN=YWHAZ PE=1 SV=1	1433Z_HUMAN	28 kDa	100%
22	NEDD8 OS=Homo sapiens GN=NEDD8 PE=1 SV=1	NEDD8_HUMAN	9 kDa	100%
23	Translational activator GCN1 OS=Homo sapiens GN=GCN1L1 PE=1 SV=6	GCN1L_HUMAN	293 kDa	100%
24	Nuclease-sensitive element-binding protein 1 OS=Homo sapiens GN=YBX1 PE=1 SV=3	YBOX1_HUMAN (+2)	36 kDa	99%
25	Leucine-rich repeat-containing protein 59 OS=Homo sapiens GN=LRRC59 PE=1 SV=1	LRC59_HUMAN	35 kDa	99%
26	Peptidyl-prolyl cis-trans isomerase FKBP4 OS=Homo sapiens GN=FKBP4 PE=1 SV=3	FKBP4_HUMAN	52 kDa	100%
27	60 kDa heat shock protein, mitochondrial OS=Homo sapiens GN=HSPD1 PE=1 SV=2	CH60_HUMAN	61 kDa	100%
28	Chromobox protein homolog 3 OS=Homo sapiens GN=CBX3 PE=1 SV=4	CBX3_HUMAN	21 kDa	100%
29	Heterogeneous nuclear ribonucleoprotein H OS=Homo sapiens GN=HNRNPH1 PE=1 SV=4	HNRH1_HUMAN	49 kDa	100%
30	Heat shock protein HSP 90-alpha OS=Homo sapiens GN=HSP90AA1 PE=1 SV=5	HS90A_HUMAN	85 kDa	100%
31	Cathepsin D OS=Homo sapiens GN=CTSD PE=1 SV=1	CATD_HUMAN	45 kDa	100%
32	Heterogeneous nuclear ribonucleoproteins A2/B1 OS=Homo sapiens GN=HNRNPA2B1 PE=1 SV=2	ROA2_HUMAN	37 kDa	100%

33	Gelsolin OS=Homo sapiens GN=GSN PE=1 SV=1	GELS_HUMAN	86 kDa	99%
34	60S ribosomal protein L10 OS=Homo sapiens GN=RPL10 PE=1 SV=4	RL10_HUMAN	25 kDa	95%
35	Annexin A2 OS=Homo sapiens GN=ANXA2 PE=1 SV=2	ANXA2_HUMAN	39 kDa	100%
36	Calnexin OS=Homo sapiens GN=CANX PE=1 SV=2	CALX_HUMAN	68 kDa	100%
37	Keratin, type I cytoskeletal 19 OS=Homo sapiens GN=KRT19 PE=1 SV=4	K1C19_HUMAN	44 kDa	100%
38	Heat shock protein HSP 90-beta OS=Homo sapiens GN=HSP90AB1 PE=1 SV=4	HS90B_HUMAN	83 kDa	100%
39	Eukaryotic translation initiation factor 2 subunit 2 OS=Homo sapiens GN=EIF2S2 PE=1 SV=2	IF2B_HUMAN	38 kDa	100%
40	Serine/arginine-rich splicing factor 3 OS=Homo sapiens GN=SRSF3 PE=1 SV=1	SRSF3_HUMAN (+1)	19 kDa	96%
41	Putative pre-mRNA-splicing factor ATP-dependent RNA helicase DHX15 OS=Homo sapiens GN=DHX15 PE=1 SV=2	DHX15_HUMAN	91 kDa	100%
42	ATP-dependent RNA helicase A OS=Homo sapiens GN=DHX9 PE=1 SV=4	DHX9_HUMAN	141 kDa	100%
43	40S ribosomal protein S7 OS=Homo sapiens GN=RPS7 PE=1 SV=1	RS7_HUMAN	22 kDa	99%
44	Plasminogen activator inhibitor 1 RNA-binding protein OS=Homo sapiens GN=SERBP1 PE=1 SV=2	PAIRB_HUMAN	45 kDa	99%
45	Protein SET OS=Homo sapiens GN=SET PE=1 SV=3	SET_HUMAN	33 kDa	100%
46	PRKC apoptosis WT1 regulator protein OS=Homo sapiens GN=PAWR PE=1 SV=1	PAWR_HUMAN	37 kDa	100%
47	60S acidic ribosomal protein P2 OS=Homo sapiens GN=RPLP2 PE=1 SV=1	RLA2_HUMAN	12 kDa	100%
48	Cluster of Protein arginine N-methyltransferase 8 OS=Homo sapiens GN=PRMT8 PE=1 SV=2 (ANM8_HUMAN)	ANM8_HUMAN [2]	45 kDa	100%
48.2	Protein arginine N-methyltransferase 1 OS=Homo sapiens GN=PRMT1 PE=1 SV=2	ANM1_HUMAN	42 kDa	91%
49	Protein disulfide-isomerase A6 OS=Homo sapiens GN=PDIA6 PE=1 SV=1	PDIA6_HUMAN	48 kDa	100%
50	Bifunctional purine biosynthesis protein PURH OS=Homo sapiens GN=ATIC PE=1 SV=3	PUR9_HUMAN	65 kDa	100%
51	Cluster of Sodium/potassium-transporting ATPase subunit alpha-1 OS=Homo sapiens GN=ATP1A1 PE=1 SV=1 (AT1A1_HUMAN)	AT1A1_HUMAN [2]	113 kDa	100%
52	Thioredoxin OS=Homo sapiens GN=TXN PE=1 SV=3	THIO_HUMAN	12 kDa	99%
53	Hematological and neurological expressed 1 protein OS=Homo sapiens GN=HN1 PE=1 SV=3	HN1_HUMAN	16 kDa	99%
54	Complement component 1 Q subcomponent-binding protein, mitochondrial OS=Homo sapiens GN=C1QBP PE=1 SV=1	C1QBP_HUMAN	31 kDa	100%
55	X-ray repair cross-complementing protein 6 OS=Homo sapiens GN=XRCC6 PE=1 SV=2	XRCC6_HUMAN	70 kDa	100%
56	Myoferlin OS=Homo sapiens GN=MYOF PE=1 SV=1	MYOF_HUMAN	235 kDa	100%

57	L-lactate dehydrogenase A chain OS=Homo sapiens GN=LDHA PE=1 SV=2	LDHA_HUMAN	37 kDa	99%
58	Neuron navigator 1 OS=Homo sapiens GN=NAV1 PE=1 SV=2	NAV1_HUMAN	202 kDa	99%
59	Plectin OS=Homo sapiens GN=PLEC PE=1 SV=3	PLEC_HUMAN	532 kDa	100%
60	SUMO-activating enzyme subunit 2 OS=Homo sapiens GN=UBA2 PE=1 SV=2	SAE2_HUMAN	71 kDa	100%
61	40S ribosomal protein S3 OS=Homo sapiens GN=RPS3 PE=1 SV=2	RS3_HUMAN	27 kDa	100%
62	Actin-related protein 3 OS=Homo sapiens GN=ACTR3 PE=1 SV=3	ARP3_HUMAN	47 kDa	100%
63	Heterogeneous nuclear ribonucleoprotein A1-like 2 OS=Homo sapiens GN=HNRNPA1L2 PE=2 SV=2	RA1L2_HUMAN (+1)	34 kDa	100%
64	Bifunctional 3'-phosphoadenosine 5'-phosphosulfate synthase 2 OS=Homo sapiens GN=PAPSS2 PE=1 SV=2	PAPS2_HUMAN	70 kDa	100%
65	Triosephosphate isomerase OS=Homo sapiens GN=TPI1 PE=1 SV=3	TPIS_HUMAN	31 kDa	100%
66	Ubiquitin-like modifier-activating enzyme 1 OS=Homo sapiens GN=UBA1 PE=1 SV=3	UBA1_HUMAN	118 kDa	100%
67	Calmodulin OS=Homo sapiens GN=CALM1 PE=1 SV=2	CALM_HUMAN	17 kDa	100%
68	Na(+)/H(+) exchange regulatory cofactor NHE-RF1 OS=Homo sapiens GN=SLC9A3R1 PE=1 SV=4	NHRF1_HUMAN	39 kDa	100%
69	Exportin-2 OS=Homo sapiens GN=CSE1L PE=1 SV=3	XPO2_HUMAN	110 kDa	100%
70	Isocitrate dehydrogenase [NADP], mitochondrial OS=Homo sapiens GN=IDH2 PE=1 SV=2	IDHP_HUMAN	51 kDa	100%
71	Serrate RNA effector molecule homolog OS=Homo sapiens GN=SRRT PE=1 SV=1	SRRT_HUMAN	101 kDa	100%
72	Apolipoprotein B-100 OS=Homo sapiens GN=APOB PE=1 SV=2	APOB_HUMAN	516 kDa	100%
73	Serine/arginine-rich splicing factor 5 OS=Homo sapiens GN=SRSF5 PE=1 SV=1	SRSF5_HUMAN	31 kDa	99%
74	Small nuclear ribonucleoprotein E OS=Homo sapiens GN=SNRPE PE=1 SV=1	RUXE_HUMAN	11 kDa	97%
75	14-3-3 protein gamma OS=Homo sapiens GN=YWHAG PE=1 SV=2	1433G_HUMAN	28 kDa	100%
76	Putative tropomyosin alpha-3 chain-like protein OS=Homo sapiens PE=5 SV=2	TPM3L_HUMAN	26 kDa	100%
77	Protein disulfide-isomerase OS=Homo sapiens GN=P4HB PE=1 SV=3	PDIA1_HUMAN	57 kDa	99%
78	Sorting nexin-3 OS=Homo sapiens GN=SNX3 PE=1 SV=3	SNX3_HUMAN	19 kDa	99%
79	Proliferating cell nuclear antigen OS=Homo sapiens GN=PCNA PE=1 SV=1	PCNA_HUMAN	29 kDa	99%
80	Heterogeneous nuclear ribonucleoprotein Q OS=Homo sapiens GN=SYNCRIP PE=1 SV=2	HNRPQ_HUMAN	70 kDa	100%
81	Staphylococcal nuclease domain-containing protein 1 OS=Homo sapiens GN=SND1 PE=1 SV=1	SND1_HUMAN	102 kDa	100%
82	40S ribosomal protein S17-like OS=Homo sapiens GN=RPS17L PE=2 SV=1	RS17L_HUMAN (+1)	16 kDa	100%

83	SUMO-activating enzyme subunit 1 OS=Homo sapiens GN=SAE1 PE=1 SV=1	SAE1_HUMAN	38 kDa	99%
84	Cytosol aminopeptidase OS=Homo sapiens GN=LAP3 PE=1 SV=3	AMPL_HUMAN	56 kDa	99%
85	Heat shock protein beta-8 OS=Homo sapiens GN=HSPB8 PE=1 SV=1	HSPB8_HUMAN	22 kDa	98%
86	A-kinase anchor protein 8-like OS=Homo sapiens GN=AKAP8L PE=1 SV=3	AKP8L_HUMAN	72 kDa	98%
87	ATP-dependent RNA helicase DDX39A OS=Homo sapiens GN=DDX39A PE=1 SV=2	DX39A_HUMAN (+1)	49 kDa	97%
88	Basic leucine zipper and W2 domain-containing protein 2 OS=Homo sapiens GN=BZW2 PE=1 SV=1	BZW2_HUMAN	48 kDa	96%
89	Postacrosomal sheath WW domain-binding protein OS=Homo sapiens GN=WBP2NL PE=2 SV=1	WBP2L_HUMAN	32 kDa	91%
90	Dolichyl-diphosphooligosaccharide--protein glycosyltransferase subunit 1 OS=Homo sapiens GN=RPN1 PE=1 SV=1	RPN1_HUMAN	69 kDa	100%
91	14-3-3 protein eta OS=Homo sapiens GN=YWHAH PE=1 SV=4	1433F_HUMAN	28 kDa	100%
92	Vesicular integral-membrane protein VIP36 OS=Homo sapiens GN=LMAN2 PE=1 SV=1	LMAN2_HUMAN	40 kDa	100%
93	Chloride intracellular channel protein 1 OS=Homo sapiens GN=CLIC1 PE=1 SV=4	CLIC1_HUMAN	27 kDa	99%
94	Glutaredoxin-3 OS=Homo sapiens GN=GLRX3 PE=1 SV=2	GLRX3_HUMAN	37 kDa	99%
95	Glutamine--fructose-6-phosphate aminotransferase [isomerizing] 1 OS=Homo sapiens GN=GFPT1 PE=1 SV=3	GFPT1_HUMAN	79 kDa	99%
96	Heterogeneous nuclear ribonucleoprotein A3 OS=Homo sapiens GN=HNRNPA3 PE=1 SV=2	ROA3_HUMAN	40 kDa	99%
97	Peroxiredoxin-5, mitochondrial OS=Homo sapiens GN=PRDX5 PE=1 SV=4	PRDX5_HUMAN	22 kDa	99%
98	Splicing factor 3B subunit 3 OS=Homo sapiens GN=SF3B3 PE=1 SV=4	SF3B3_HUMAN	136 kDa	99%
99	Stathmin OS=Homo sapiens GN=STMN1 PE=1 SV=3	STMN1_HUMAN	17 kDa	99%
100	Lamin-B1 OS=Homo sapiens GN=LMNB1 PE=1 SV=2	LMNB1_HUMAN	66 kDa	98%
101	Kynurenine 3-monooxygenase OS=Homo sapiens GN=KMO PE=1 SV=2	KMO_HUMAN	56 kDa	91%
102	DBIRD complex subunit KIAA1967 OS=Homo sapiens GN=KIAA1967 PE=1 SV=2	K1967_HUMAN	103 kDa	100%
103	DNA replication licensing factor MCM3 OS=Homo sapiens GN=MCM3 PE=1 SV=3	MCM3_HUMAN	91 kDa	100%
104	F-actin-capping protein subunit alpha-1 OS=Homo sapiens GN=CAPZA1 PE=1 SV=3	CAZA1_HUMAN	33 kDa	100%
105	Calpain-1 catalytic subunit OS=Homo sapiens GN=CAPN1 PE=1 SV=1	CAN1_HUMAN	82 kDa	100%
106	Interleukin enhancer-binding factor 3 OS=Homo sapiens GN=ILF3 PE=1 SV=3	ILF3_HUMAN	95 kDa	100%
107	Myristoylated alanine-rich C-kinase substrate OS=Homo sapiens GN=MARCKS PE=1 SV=4	MARCS_HUMAN	32 kDa	100%
108	Calcium-regulated heat stable protein 1 OS=Homo sapiens GN=CARHSP1 PE=1 SV=2	CHSP1_HUMAN	16 kDa	100%

109	40S ribosomal protein S3a OS=Homo sapiens GN=RPS3A PE=1 SV=2	RS3A_HUMAN	30 kDa	100%
110	14-3-3 protein epsilon OS=Homo sapiens GN=YWHAE PE=1 SV=1	1433E_HUMAN	29 kDa	100%
111	BolA-like protein 2 OS=Homo sapiens GN=BOLA2 PE=1 SV=1	BOLA2_HUMAN	10 kDa	99%
112	40S ribosomal protein S14 OS=Homo sapiens GN=RPS14 PE=1 SV=3	RS14_HUMAN	16 kDa	99%
113	Stomatin-like protein 2, mitochondrial OS=Homo sapiens GN=STOML2 PE=1 SV=1	STML2_HUMAN	39 kDa	99%
114	Hypoxia up-regulated protein 1 OS=Homo sapiens GN=HYOU1 PE=1 SV=1	HYOU1_HUMAN	111 kDa	99%
115	Heterogeneous nuclear ribonucleoprotein A/B OS=Homo sapiens GN=HNRNPAB PE=1 SV=2	ROAA_HUMAN	36 kDa	99%
116	Eukaryotic translation initiation factor 4 gamma 1 OS=Homo sapiens GN=EIF4G1 PE=1 SV=4	IF4G1_HUMAN	175 kDa	99%
117	Opioid growth factor receptor OS=Homo sapiens GN=OGFR PE=1 SV=3	OGFR_HUMAN	73 kDa	100%
118	Leucine-rich PPR motif-containing protein, mitochondrial OS=Homo sapiens GN=LRPPRC PE=1 SV=3	LPPRC_HUMAN	158 kDa	100%
119	N-alpha-acetyltransferase 10 OS=Homo sapiens GN=NAA10 PE=1 SV=1	NAA10_HUMAN	26 kDa	100%
120	DNA ligase 1 OS=Homo sapiens GN=LIG1 PE=1 SV=1	DNLI1_HUMAN	102 kDa	100%
121	Golgi to ER traffic protein 4 homolog OS=Homo sapiens GN=GET4 PE=1 SV=1	GET4_HUMAN	37 kDa	99%
122	PCTP-like protein OS=Homo sapiens GN=STARD10 PE=1 SV=2	PCTL_HUMAN	33 kDa	99%
123	Neutral alpha-glucosidase AB OS=Homo sapiens GN=GANAB PE=1 SV=3	GANAB_HUMAN	107 kDa	99%
124	Collagen alpha-1(I) chain OS=Homo sapiens GN=COL1A1 PE=1 SV=5	CO1A1_HUMAN	139 kDa	99%
125	Endoplasmin OS=Homo sapiens GN=HSP90B1 PE=1 SV=1	ENPL_HUMAN	92 kDa	98%
126	Transformer-2 protein homolog beta OS=Homo sapiens GN=TRA2B PE=1 SV=1	TRA2B_HUMAN	34 kDa	96%
127	DNA-(apurinic or apyrimidinic site) lyase OS=Homo sapiens GN=APEX1 PE=1 SV=2	APEX1_HUMAN	36 kDa	99%
128	Uncharacterized protein C7orf50 OS=Homo sapiens GN=C7orf50 PE=1 SV=1	CG050_HUMAN	22 kDa	99%
129	Nuclear autoantigenic sperm protein OS=Homo sapiens GN=NASP PE=1 SV=2	NASP_HUMAN	85 kDa	99%
130	T-complex protein 1 subunit beta OS=Homo sapiens GN=CCT2 PE=1 SV=4	TCPB_HUMAN	57 kDa	99%
131	UBX domain-containing protein 1 OS=Homo sapiens GN=UBXN1 PE=1 SV=2	UBXN1_HUMAN	33 kDa	99%
132	Cytochrome b-c1 complex subunit 7 OS=Homo sapiens GN=UQCRB PE=1 SV=2	QCR7_HUMAN	14 kDa	99%
133	Glycogen phosphorylase, brain form OS=Homo sapiens GN=PYGB PE=1 SV=5	PYGB_HUMAN	97 kDa	99%
134	14-3-3 protein sigma OS=Homo sapiens GN=SFN PE=1 SV=1	1433S_HUMAN	28 kDa	99%

135	Myosin-10 OS=Homo sapiens GN=MYH10 PE=1 SV=3	MYH10_HUMAN	229 kDa	99%
136	Gem-associated protein 5 OS=Homo sapiens GN=GEMIN5 PE=1 SV=3	GEMI5_HUMAN	169 kDa	98%
137	Cell division control protein 42 homolog OS=Homo sapiens GN=CDC42 PE=1 SV=2	CDC42_HUMAN	21 kDa	95%
138	Fragile X mental retardation syndrome-related protein 1 OS=Homo sapiens GN=FXR1 PE=1 SV=3	FXR1_HUMAN	70 kDa	100%
139	A-kinase anchor protein 8 OS=Homo sapiens GN=AKAP8 PE=1 SV=1	AKAP8_HUMAN	76 kDa	99%
140	Platelet-activating factor acetylhydrolase IB subunit alpha OS=Homo sapiens GN=PAFAH1B1 PE=1 SV=2	LIS1_HUMAN	47 kDa	99%
141	Prefoldin subunit 4 OS=Homo sapiens GN=PFDN4 PE=1 SV=1	PFD4_HUMAN	15 kDa	99%
142	Sorting nexin-6 OS=Homo sapiens GN=SNX6 PE=1 SV=1	SNX6_HUMAN	47 kDa	99%
143	6-phosphofructokinase type C OS=Homo sapiens GN=PFKP PE=1 SV=2	K6PP_HUMAN	86 kDa	99%
144	Membrane-associated progesterone receptor component 1 OS=Homo sapiens GN=PGRMC1 PE=1 SV=3	PGRC1_HUMAN	22 kDa	99%
145	Large proline-rich protein BAG6 OS=Homo sapiens GN=BAG6 PE=1 SV=2	BAG6_HUMAN	119 kDa	99%
146	Glucosidase 2 subunit beta OS=Homo sapiens GN=PRKCSH PE=1 SV=2	GLU2B_HUMAN	59 kDa	98%
147	Eukaryotic translation initiation factor 5A-1 OS=Homo sapiens GN=EIF5A PE=1 SV=2	IF5A1_HUMAN	17 kDa	98%
148	Semaphorin-4C OS=Homo sapiens GN=SEMA4C PE=2 SV=2	SEM4C_HUMAN	93 kDa	98%
149	26S proteasome non-ATPase regulatory subunit 6 OS=Homo sapiens GN=PSMD6 PE=1 SV=1	PSMD6_HUMAN	46 kDa	98%
150	Clathrin heavy chain 1 OS=Homo sapiens GN=CLTC PE=1 SV=5	CLH1_HUMAN	192 kDa	98%
151	Protein FAM162A OS=Homo sapiens GN=FAM162A PE=1 SV=2	F162A_HUMAN	17 kDa	97%
152	Dual specificity mitogen-activated protein kinase kinase 2 OS=Homo sapiens GN=MAP2K2 PE=1 SV=1	MP2K2_HUMAN	44 kDa	94%
153	Ribonuclease inhibitor OS=Homo sapiens GN=RNH1 PE=1 SV=2	RINI_HUMAN	50 kDa	94%
154	ATP synthase subunit e, mitochondrial OS=Homo sapiens GN=ATP5I PE=1 SV=2	ATP5I_HUMAN	8 kDa	93%
155	Histone H2A.V OS=Homo sapiens GN=H2AFV PE=1 SV=3	H2AV_HUMAN (+1)	14 kDa	93%
156	Coiled-coil domain-containing protein 90B, mitochondrial OS=Homo sapiens GN=CCDC90B PE=1 SV=2	CC90B_HUMAN	30 kDa	92%
157	26S proteasome non-ATPase regulatory subunit 2 OS=Homo sapiens GN=PSMD2 PE=1 SV=3	PSMD2_HUMAN	100 kDa	99%
158	Transcription activator BRG1 OS=Homo sapiens GN=SMARCA4 PE=1 SV=2	SMCA4_HUMAN	185 kDa	99%
159	Gamma-taxilin OS=Homo sapiens GN=TXLNG PE=1 SV=2	TXLNG_HUMAN	61 kDa	99%
160	Transcription elongation factor B polypeptide 2 OS=Homo sapiens GN=TCEB2 PE=1 SV=1	ELOB_HUMAN	13 kDa	97%

161	Drebrin-like protein OS=Homo sapiens GN=DBNL PE=1 SV=1	DBNL_HUMAN	48 kDa	96%
162	CD2 antigen cytoplasmic tail-binding protein 2 OS=Homo sapiens GN=CD2BP2 PE=1 SV=1	CD2B2_HUMAN	38 kDa	95%
163	Lon protease homolog, mitochondrial OS=Homo sapiens GN=LONP1 PE=1 SV=2	LONM_HUMAN	106 kDa	94%
164	Eukaryotic translation initiation factor 6 OS=Homo sapiens GN=EIF6 PE=1 SV=1	IF6_HUMAN	27 kDa	94%
165	Prolactin regulatory element-binding protein OS=Homo sapiens GN=PREB PE=1 SV=2	PREB_HUMAN	45 kDa	94%
166	GrpE protein homolog 1, mitochondrial OS=Homo sapiens GN=GRPEL1 PE=1 SV=2	GRPE1_HUMAN	24 kDa	94%
167	ATP synthase subunit gamma, mitochondrial OS=Homo sapiens GN=ATP5C1 PE=1 SV=1	ATPG_HUMAN	33 kDa	94%
168	Nuclear cap-binding protein subunit 1 OS=Homo sapiens GN=NCBP1 PE=1 SV=1	NCBP1_HUMAN	92 kDa	93%
169	39S ribosomal protein L41, mitochondrial OS=Homo sapiens GN=MRPL41 PE=1 SV=1	RM41_HUMAN	15 kDa	93%
170	WASH complex subunit FAM21A OS=Homo sapiens GN=FAM21A PE=2 SV=3	FA21A_HUMAN (+2)	147 kDa	92%
171	Prelamin-A/C OS=Homo sapiens GN=LMNA PE=1 SV=1	LMNA_HUMAN	74 kDa	92%
172	Fructose-bisphosphate aldolase A OS=Homo sapiens GN=ALDOA PE=1 SV=2	ALDOA_HUMAN	39 kDa	92%
173	Microtubule-associated protein tau OS=Homo sapiens GN=MAPT PE=1 SV=5	TAU_HUMAN	79 kDa	92%
174	Nucleophosmin OS=Homo sapiens GN=NPM1 PE=1 SV=2	NPM_HUMAN	33 kDa	92%
175	1,2-dihydroxy-3-keto-5-methylthiopentene dioxygenase OS=Homo sapiens GN=ADI1 PE=1 SV=1	MTND_HUMAN	21 kDa	91%
176	Sorting and assembly machinery component 50 homolog OS=Homo sapiens GN=SAMM50 PE=1 SV=3	SAM50_HUMAN	52 kDa	91%
177	Neuroblast differentiation-associated protein AHNAK OS=Homo sapiens GN=AHNAK PE=1 SV=2	AHNK_HUMAN	629 kDa	100%
178	Cluster of Heat shock protein HSP 90-alpha OS=Homo sapiens GN=HSP90AA1 PE=1 SV=5 (HS90A_HUMAN)	HS90A_HUMAN [3]	85 kDa	100%
179	Keratin, type II cytoskeletal 8 OS=Homo sapiens GN=KRT8 PE=1 SV=7	K2C8_HUMAN	54 kDa	100%
180	Cluster of Heat shock 70 kDa protein 1A/1B OS=Homo sapiens GN=HSPA1A PE=1 SV=5 (HSP71_HUMAN)	HSP71_HUMAN [2]	70 kDa	100%
181	Cluster of Tubulin beta chain OS=Homo sapiens GN=TUBB PE=1 SV=2 (TBB5_HUMAN)	TBB5_HUMAN [4]	50 kDa	100%
182	Cluster of Alpha-actinin-4 OS=Homo sapiens GN=ACTN4 PE=1 SV=2 (ACTN4_HUMAN)	ACTN4_HUMAN [3]	105 kDa	100%
183	Keratin, type I cytoskeletal 18 OS=Homo sapiens GN=KRT18 PE=1 SV=2	K1C18_HUMAN	48 kDa	100%
184	Tubulin alpha-1A chain OS=Homo sapiens GN=TUBA1A PE=1 SV=1	TBA1A_HUMAN (+2)	50 kDa	100%
185	Cluster of Actin, cytoplasmic 1 OS=Homo sapiens GN=ACTB PE=1 SV=1 (ACTB_HUMAN)	ACTB_HUMAN [6]	42 kDa	100%

186	Cluster of Tropomyosin alpha-4 chain OS=Homo sapiens GN=TPM4 PE=1 SV=3 (TPM4_HUMAN)	TPM4_HUMAN [3]	29 kDa	100%
187	Myosin-9 OS=Homo sapiens GN=MYH9 PE=1 SV=4	MYH9_HUMAN	227 kDa	100%
188	14-3-3 protein zeta/delta OS=Homo sapiens GN=YWHAZ PE=1 SV=1	1433Z_HUMAN	28 kDa	100%
189	Calnexin OS=Homo sapiens GN=CANX PE=1 SV=2	CALX_HUMAN	68 kDa	100%
190	Cluster of Clathrin heavy chain 1 OS=Homo sapiens GN=CLTC PE=1 SV=5 (CLH1_HUMAN)	CLH1_HUMAN [2]	192 kDa	100%
191	Profilin-1 OS=Homo sapiens GN=PFN1 PE=1 SV=2	PROF1_HUMAN	15 kDa	99%
192	Protein SEC13 homolog OS=Homo sapiens GN=SEC13 PE=1 SV=3	SEC13_HUMAN	36 kDa	99%
193	78 kDa glucose-regulated protein OS=Homo sapiens GN=HSPA5 PE=1 SV=2	GRP78_HUMAN	72 kDa	100%
194	60 kDa heat shock protein, mitochondrial OS=Homo sapiens GN=HSPD1 PE=1 SV=2	CH60_HUMAN	61 kDa	100%
195	Pyruvate kinase isozymes M1/M2 OS=Homo sapiens GN=PKM PE=1 SV=4	KPYM_HUMAN	58 kDa	100%
196	Alpha-enolase OS=Homo sapiens GN=ENO1 PE=1 SV=2	ENOA_HUMAN	47 kDa	100%
197	Nucleolin OS=Homo sapiens GN=NCL PE=1 SV=3	NUCL_HUMAN	77 kDa	100%
198	Myosin-14 OS=Homo sapiens GN=MYH14 PE=1 SV=2	MYH14_HUMAN	228 kDa	100%
199	Protein disulfide-isomerase OS=Homo sapiens GN=P4HB PE=1 SV=3	PDIA1_HUMAN	57 kDa	100%
200	Dipeptidyl peptidase 3 OS=Homo sapiens GN=DPP3 PE=1 SV=2	DPP3_HUMAN	83 kDa	99%
201	Staphylococcal nuclease domain-containing protein 1 OS=Homo sapiens GN=SND1 PE=1 SV=1	SND1_HUMAN	102 kDa	100%
202	DNA-dependent protein kinase catalytic subunit OS=Homo sapiens GN=PRKDC PE=1 SV=3	PRKDC_HUMAN	469 kDa	100%
203	Filamin-A OS=Homo sapiens GN=FLNA PE=1 SV=4	FLNA_HUMAN	281 kDa	100%
204	Plectin OS=Homo sapiens GN=PLEC PE=1 SV=3	PLEC_HUMAN	532 kDa	100%
205	Fructose-bisphosphate aldolase C OS=Homo sapiens GN=ALDOC PE=1 SV=2	ALDOC_HUMAN	39 kDa	100%
206	Partner of Y14 and mago OS=Homo sapiens GN=WIBG PE=1 SV=1	WIBG_HUMAN	23 kDa	99%
207	Nucleophosmin OS=Homo sapiens GN=NPM1 PE=1 SV=2	NPM_HUMAN	33 kDa	100%
208	Peptidyl-prolyl cis-trans isomerase FKBP4 OS=Homo sapiens GN=FKBP4 PE=1 SV=3	FKBP4_HUMAN	52 kDa	100%
209	Transcription intermediary factor 1-beta OS=Homo sapiens GN=TRIM28 PE=1 SV=5	TIF1B_HUMAN	89 kDa	100%
210	Radixin OS=Homo sapiens GN=RDX PE=1 SV=1	RADI_HUMAN	69 kDa	99%
211	Annexin A2 OS=Homo sapiens GN=ANXA2 PE=1 SV=2	ANXA2_HUMAN	39 kDa	100%

212	Calreticulin OS=Homo sapiens GN=CALR PE=1 SV=1	CALR_HUMAN	48 kDa	99%
213	Thioredoxin OS=Homo sapiens GN=TXN PE=1 SV=3	THIO_HUMAN	12 kDa	100%
214	Cluster of Heat shock cognate 71 kDa protein OS=Homo sapiens GN=HSPA8 PE=1 SV=1 (HSP7C_HUMAN)	HSP7C_HUMAN	71 kDa	100%
215	SUMO-activating enzyme subunit 1 OS=Homo sapiens GN=SAE1 PE=1 SV=1	SAE1_HUMAN	38 kDa	100%
216	NEDD8 OS=Homo sapiens GN=NEDD8 PE=1 SV=1	NEDD8_HUMAN	9 kDa	100%
217	60S acidic ribosomal protein P2 OS=Homo sapiens GN=RPLP2 PE=1 SV=1	RLA2_HUMAN	12 kDa	100%
218	40S ribosomal protein S3 OS=Homo sapiens GN=RPS3 PE=1 SV=2	RS3_HUMAN	27 kDa	100%
219	14-3-3 protein epsilon OS=Homo sapiens GN=YWHAE PE=1 SV=1	1433E_HUMAN	29 kDa	100%
220	Bcl-2-associated transcription factor 1 OS=Homo sapiens GN=BCLAF1 PE=1 SV=2	BCLF1_HUMAN	106 kDa	100%
221	Complement component 1 Q subcomponent-binding protein, mitochondrial OS=Homo sapiens GN=C1QBP PE=1 SV=1	C1QBP_HUMAN	31 kDa	100%
222	Chromobox protein homolog 3 OS=Homo sapiens GN=CBX3 PE=1 SV=4	CBX3_HUMAN	21 kDa	100%
223	Protein disulfide-isomerase A4 OS=Homo sapiens GN=PDIA4 PE=1 SV=2	PDIA4_HUMAN	73 kDa	100%
224	SUMO-activating enzyme subunit 2 OS=Homo sapiens GN=UBA2 PE=1 SV=2	SAE2_HUMAN	71 kDa	100%
225	Protein unc-45 homolog A OS=Homo sapiens GN=UNC45A PE=1 SV=1	UN45A_HUMAN	103 kDa	100%
226	Heterogeneous nuclear ribonucleoprotein Q OS=Homo sapiens GN=SYNCRIP PE=1 SV=2	HNRPQ_HUMAN	70 kDa	100%
227	Prelamin-A/C OS=Homo sapiens GN=LMNA PE=1 SV=1	LMNA_HUMAN	74 kDa	100%
228	Serine/arginine-rich splicing factor 5 OS=Homo sapiens GN=SRSF5 PE=1 SV=1	SRSF5_HUMAN	31 kDa	99%
229	26S proteasome non-ATPase regulatory subunit 11 OS=Homo sapiens GN=PSMD11 PE=1 SV=3	PSD11_HUMAN	47 kDa	100%
230	6-phosphogluconate dehydrogenase, decarboxylating OS=Homo sapiens GN=PGD PE=1 SV=3	6PGD_HUMAN	53 kDa	100%
231	Myoferlin OS=Homo sapiens GN=MYOF PE=1 SV=1	MYOF_HUMAN	235 kDa	100%
232	Protein disulfide-isomerase A6 OS=Homo sapiens GN=PDIA6 PE=1 SV=1	PDIA6_HUMAN	48 kDa	100%
233	Calmodulin OS=Homo sapiens GN=CALM1 PE=1 SV=2	CALM_HUMAN	17 kDa	100%
234	Cathepsin D OS=Homo sapiens GN=CTSD PE=1 SV=1	CATD_HUMAN	45 kDa	100%
235	Spectrin alpha chain, non-erythrocytic 1 OS=Homo sapiens GN=SPTAN1 PE=1 SV=3	SPTN1_HUMAN	285 kDa	100%
236	Na(+)/H(+) exchange regulatory cofactor NHE-RF1 OS=Homo sapiens GN=SLC9A3R1 PE=1 SV=4	NHRF1_HUMAN	39 kDa	100%
237	60S ribosomal protein L4 OS=Homo sapiens GN=RPL4 PE=1 SV=5	RL4_HUMAN	48 kDa	100%

238	Anterior gradient protein 2 homolog OS=Homo sapiens GN=AGR2 PE=1 SV=1	AGR2_HUMAN	20 kDa	99%
239	Protein SET OS=Homo sapiens GN=SET PE=1 SV=3	SET_HUMAN	33 kDa	100%
240	X-ray repair cross-complementing protein 5 OS=Homo sapiens GN=XRCC5 PE=1 SV=3	XRCC5_HUMAN	83 kDa	100%
241	Serine/arginine-rich splicing factor 2 OS=Homo sapiens GN=SRSF2 PE=1 SV=4	SRSF2_HUMAN	25 kDa	99%
242	Proliferation-associated protein 2G4 OS=Homo sapiens GN=PA2G4 PE=1 SV=3	PA2G4_HUMAN	44 kDa	99%
243	Ubiquitin-like modifier-activating enzyme 1 OS=Homo sapiens GN=UBA1 PE=1 SV=3	UBA1_HUMAN	118 kDa	100%
244	Lamin-B1 OS=Homo sapiens GN=LMNB1 PE=1 SV=2	LMNB1_HUMAN	66 kDa	100%
245	Ras GTPase-activating-like protein IQGAP1 OS=Homo sapiens GN=IQGAP1 PE=1 SV=1	IQGA1_HUMAN	189 kDa	98%
246	Src substrate cortactin OS=Homo sapiens GN=CTTN PE=1 SV=2	SRC8_HUMAN	62 kDa	100%
247	Cluster of Y-box-binding protein 3 OS=Homo sapiens GN=YBX3 PE=1 SV=4 (YBOX3_HUMAN)	YBOX3_HUMAN	40 kDa	99%
248	Cold shock domain-containing protein E1 OS=Homo sapiens GN=CSDE1 PE=1 SV=2	CSDE1_HUMAN	89 kDa	100%
249	Keratin, type I cytoskeletal 19 OS=Homo sapiens GN=KRT19 PE=1 SV=4	K1C19_HUMAN	44 kDa	100%
250	Prefoldin subunit 4 OS=Homo sapiens GN=PFDN4 PE=1 SV=1	PFD4_HUMAN	15 kDa	100%
251	Pre-mRNA-processing factor 40 homolog A OS=Homo sapiens GN=PRPF40A PE=1 SV=2	PR40A_HUMAN	109 kDa	100%
252	Heterogeneous nuclear ribonucleoprotein A3 OS=Homo sapiens GN=HNRNPA3 PE=1 SV=2	ROA3_HUMAN	40 kDa	100%
253	pre-rRNA processing protein FTSJ3 OS=Homo sapiens GN=FTSJ3 PE=1 SV=2	SPB1_HUMAN	97 kDa	100%
254	Transitional endoplasmic reticulum ATPase OS=Homo sapiens GN=VCP PE=1 SV=4	TERA_HUMAN	89 kDa	100%
255	Filamin-B OS=Homo sapiens GN=FLNB PE=1 SV=2	FLNB_HUMAN	278 kDa	99%
256	Ubiquitin-conjugating enzyme E2 variant 1 OS=Homo sapiens GN=UBE2V1 PE=1 SV=2	UB2V1_HUMAN	16 kDa	99%
257	Fructose-bisphosphate aldolase A OS=Homo sapiens GN=ALDOA PE=1 SV=2	ALDOA_HUMAN	39 kDa	100%
258	Heterogeneous nuclear ribonucleoproteins A2/B1 OS=Homo sapiens GN=HNRNPA2B1 PE=1 SV=2	ROA2_HUMAN	37 kDa	99%
259	Isocitrate dehydrogenase [NADP], mitochondrial OS=Homo sapiens GN=IDH2 PE=1 SV=2	IDHP_HUMAN	51 kDa	100%
260	Triosephosphate isomerase OS=Homo sapiens GN=TPI1 PE=1 SV=3	TPIS_HUMAN	31 kDa	100%
261	Ubiquitin carboxyl-terminal hydrolase 14 OS=Homo sapiens GN=USP14 PE=1 SV=3	UBP14_HUMAN	56 kDa	99%
262	Peroxiredoxin-6 OS=Homo sapiens GN=PRDX6 PE=1 SV=3	PRDX6_HUMAN	25 kDa	100%
263	X-ray repair cross-complementing protein 6 OS=Homo sapiens GN=XRCC6 PE=1 SV=2	XRCC6_HUMAN	70 kDa	100%

264	26S proteasome non-ATPase regulatory subunit 6 OS=Homo sapiens GN=PSMD6 PE=1 SV=1	PSMD6_HUMAN	46 kDa	100%
265	Elongation factor 2 OS=Homo sapiens GN=EEF2 PE=1 SV=4	EF2_HUMAN	95 kDa	100%
266	40S ribosomal protein S14 OS=Homo sapiens GN=RPS14 PE=1 SV=3	RS14_HUMAN	16 kDa	99%
267	Glyceraldehyde-3-phosphate dehydrogenase OS=Homo sapiens GN=GAPDH PE=1 SV=3	G3P_HUMAN	36 kDa	100%
268	Putative tropomyosin alpha-3 chain-like protein OS=Homo sapiens PE=5 SV=2	TPM3L_HUMAN	26 kDa	100%
269	Eukaryotic translation initiation factor 2 subunit 2 OS=Homo sapiens GN=EIF2S2 PE=1 SV=2	IF2B_HUMAN	38 kDa	100%
270	Neutral alpha-glucosidase AB OS=Homo sapiens GN=GANAB PE=1 SV=3	GANAB_HUMAN	107 kDa	100%
271	Heterogeneous nuclear ribonucleoproteins C1/C2 OS=Homo sapiens GN=HNRNPC PE=1 SV=4	HNRPC_HUMAN	34 kDa	100%
272	Peptidyl-prolyl cis-trans isomerase A OS=Homo sapiens GN=PPIA PE=1 SV=2	PPIA_HUMAN	18 kDa	100%
273	Putative pre-mRNA-splicing factor ATP-dependent RNA helicase DHX15 OS=Homo sapiens GN=DHX15 PE=1 SV=2	DHX15_HUMAN	91 kDa	100%
274	Cluster of Interleukin enhancer-binding factor 3 OS=Homo sapiens GN=ILF3 PE=1 SV=3 (ILF3_HUMAN)	ILF3_HUMAN	95 kDa	100%
275	Programmed cell death 6-interacting protein OS=Homo sapiens GN=PDCD6IP PE=1 SV=1	PDC6I_HUMAN	96 kDa	100%
276	Vesicle-trafficking protein SEC22b OS=Homo sapiens GN=SEC22B PE=1 SV=4	SC22B_HUMAN	25 kDa	93%
277	Splicing factor 3B subunit 3 OS=Homo sapiens GN=SF3B3 PE=1 SV=4	SF3B3_HUMAN	136 kDa	100%
278	Fatty acid synthase OS=Homo sapiens GN=FASN PE=1 SV=3	FAS_HUMAN	273 kDa	100%
279	Heterogeneous nuclear ribonucleoprotein A1-like 2 OS=Homo sapiens GN=HNRNPA1L2 PE=2 SV=2	RA1L2_HUMAN (+1)	34 kDa	100%
280	F-actin-capping protein subunit alpha-1 OS=Homo sapiens GN=CAPZA1 PE=1 SV=3	CAZA1_HUMAN	33 kDa	99%
281	Eukaryotic translation initiation factor 4 gamma 1 OS=Homo sapiens GN=EIF4G1 PE=1 SV=4	IF4G1_HUMAN	175 kDa	100%
282	ATP-dependent RNA helicase A OS=Homo sapiens GN=DHX9 PE=1 SV=4	DHX9_HUMAN	141 kDa	100%
283	BolA-like protein 2 OS=Homo sapiens GN=BOLA2 PE=1 SV=1	BOLA2_HUMAN	10 kDa	100%
284	Ubiquitin-60S ribosomal protein L40 OS=Homo sapiens GN=UBA52 PE=1 SV=2	RL40_HUMAN (+3)	15 kDa	99%
285	Fragile X mental retardation syndrome-related protein 1 OS=Homo sapiens GN=FXR1 PE=1 SV=3	FXR1_HUMAN	70 kDa	100%
286	4F2 cell-surface antigen heavy chain OS=Homo sapiens GN=SLC3A2 PE=1 SV=3	4F2_HUMAN	68 kDa	100%
287	Bifunctional purine biosynthesis protein PURH OS=Homo sapiens GN=ATIC PE=1 SV=3	PUR9_HUMAN	65 kDa	100%
288	C-1-tetrahydrofolate synthase, cytoplasmic OS=Homo sapiens GN=MTHFD1 PE=1 SV=3	C1TC_HUMAN	102 kDa	100%
289	14-3-3 protein eta OS=Homo sapiens GN=YWHAH PE=1 SV=4	1433F_HUMAN	28 kDa	100%

290	Heat shock protein beta-1 OS=Homo sapiens GN=HSPB1 PE=1 SV=2	HSPB1_HUMAN	23 kDa	100%
291	Cluster of Sodium/potassium-transporting ATPase subunit alpha-1 OS=Homo sapiens GN=ATP1A1 PE=1 SV=1 (AT1A1_HUMAN)	AT1A1_HUMAN	113 kDa	100%
292	Glycogen phosphorylase, brain form OS=Homo sapiens GN=PYGB PE=1 SV=5	PYGB_HUMAN	97 kDa	100%
293	C-Jun-amino-terminal kinase-interacting protein 4 OS=Homo sapiens GN=SPAG9 PE=1 SV=4	JIP4_HUMAN	146 kDa	100%
294	Proteasome activator complex subunit 2 OS=Homo sapiens GN=PSME2 PE=1 SV=4	PSME2_HUMAN	27 kDa	100%
295	Glutaredoxin-3 OS=Homo sapiens GN=GLRX3 PE=1 SV=2	GLRX3_HUMAN	37 kDa	100%
296	Cluster of Protein arginine N-methyltransferase 1 OS=Homo sapiens GN=PRMT1 PE=1 SV=2 (ANM1_HUMAN)	ANM1_HUMAN [2]	42 kDa	100%
297	Heterogeneous nuclear ribonucleoprotein K OS=Homo sapiens GN=HNRNPK PE=1 SV=1	HNRPK_HUMAN	51 kDa	99%
298	Eukaryotic translation initiation factor 3 subunit B OS=Homo sapiens GN=EIF3B PE=1 SV=3	EIF3B_HUMAN	92 kDa	100%
299	26S protease regulatory subunit 6A OS=Homo sapiens GN=PSMC3 PE=1 SV=3	PRS6A_HUMAN	49 kDa	99%
300	Cluster of Keratin, type I cytoskeletal 14 OS=Homo sapiens GN=KRT14 PE=1 SV=4 (K1C14_HUMAN)	K1C14_HUMAN [3]	52 kDa	100%
301	Lupus La protein OS=Homo sapiens GN=SSB PE=1 SV=2	LA_HUMAN	47 kDa	100%
302	Myristoylated alanine-rich C-kinase substrate OS=Homo sapiens GN=MARCKS PE=1 SV=4	MARCS_HUMAN	32 kDa	79%
303	UDP-glucose 6-dehydrogenase OS=Homo sapiens GN=UGDH PE=1 SV=1	UGDH_HUMAN	55 kDa	100%
304	14-3-3 protein gamma OS=Homo sapiens GN=YWHAG PE=1 SV=2	1433G_HUMAN	28 kDa	100%
305	Dolichyl-diphosphooligosaccharide--protein glycosyltransferase subunit 1 OS=Homo sapiens GN=RPN1 PE=1 SV=1	RPN1_HUMAN	69 kDa	100%
306	Calpastatin OS=Homo sapiens GN=CAST PE=1 SV=4	ICAL_HUMAN	77 kDa	100%
307	Gelsolin OS=Homo sapiens GN=GSN PE=1 SV=1	GELS_HUMAN	86 kDa	100%
308	Cytochrome b-c1 complex subunit 7 OS=Homo sapiens GN=UQCRB PE=1 SV=2	QCR7_HUMAN	14 kDa	100%
309	Bifunctional 3'-phosphoadenosine 5'-phosphosulfate synthase 2 OS=Homo sapiens GN=PAPSS2 PE=1 SV=2	PAPS2_HUMAN	70 kDa	100%
310	Endoplasmin OS=Homo sapiens GN=HSP90B1 PE=1 SV=1	ENPL_HUMAN	92 kDa	100%
311	PDZ domain-containing protein GIPC1 OS=Homo sapiens GN=GIPC1 PE=1 SV=2	GIPC1_HUMAN	36 kDa	100%
312	Aspartate--tRNA ligase, cytoplasmic OS=Homo sapiens GN=DARS PE=1 SV=2	SYDC_HUMAN	57 kDa	99%
313	Rho GDP-dissociation inhibitor 1 OS=Homo sapiens GN=ARHGDIA PE=1 SV=3	GDIR1_HUMAN	23 kDa	100%
314	Nuclear autoantigenic sperm protein OS=Homo sapiens GN=NASP PE=1 SV=2	NASP_HUMAN	85 kDa	100%
315	Tyrosine--tRNA ligase, cytoplasmic OS=Homo sapiens GN=YARS PE=1 SV=4	SYYC_HUMAN	59 kDa	100%

316	Superkiller viralicidic activity 2-like 2 OS=Homo sapiens GN=SKIV2L2 PE=1 SV=3	SK2L2_HUMAN	118 kDa	100%
317	Tumor protein D54 OS=Homo sapiens GN=TPD52L2 PE=1 SV=2	TPD54_HUMAN	22 kDa	99%
318	Stomatin-like protein 2, mitochondrial OS=Homo sapiens GN=STOML2 PE=1 SV=1	STML2_HUMAN	39 kDa	99%
319	Transcription activator BRG1 OS=Homo sapiens GN=SMARCA4 PE=1 SV=2	SMCA4_HUMAN	185 kDa	100%
320	Plasminogen activator inhibitor 1 RNA-binding protein OS=Homo sapiens GN=SERBP1 PE=1 SV=2	PAIRB_HUMAN	45 kDa	100%
321	Heterogeneous nuclear ribonucleoprotein R OS=Homo sapiens GN=HNRNPR PE=1 SV=1	HNRPR_HUMAN	71 kDa	100%
322	Prostaglandin E synthase 3 OS=Homo sapiens GN=PTGES3 PE=1 SV=1	TEBP_HUMAN	19 kDa	100%
323	DNA-(apurinic or apyrimidinic site) lyase OS=Homo sapiens GN=APEX1 PE=1 SV=2	APEX1_HUMAN	36 kDa	99%
324	Uncharacterized protein C7orf50 OS=Homo sapiens GN=C7orf50 PE=1 SV=1	CG050_HUMAN	22 kDa	99%
325	14-3-3 protein sigma OS=Homo sapiens GN=SFN PE=1 SV=1	1433S_HUMAN	28 kDa	100%
326	Heterogeneous nuclear ribonucleoprotein D0 OS=Homo sapiens GN=HNRNPD PE=1 SV=1	HNRPD_HUMAN	38 kDa	93%
327	14-3-3 protein theta OS=Homo sapiens GN=YWHAQ PE=1 SV=1	1433T_HUMAN	28 kDa	100%
328	60S ribosomal protein L23a OS=Homo sapiens GN=RPL23A PE=1 SV=1	RL23A_HUMAN	18 kDa	100%
329	Exportin-2 OS=Homo sapiens GN=CSE1L PE=1 SV=3	XPO2_HUMAN	110 kDa	100%
330	Cluster of Spliceosome RNA helicase DDX39B OS=Homo sapiens GN=DDX39B PE=1 SV=1 (DX39B_HUMAN)	DX39B_HUMAN [2]	49 kDa	100%
331	Fructose-1,6-bisphosphatase 1 OS=Homo sapiens GN=FBP1 PE=1 SV=5	F16P1_HUMAN	37 kDa	100%
332	L-lactate dehydrogenase A chain OS=Homo sapiens GN=LDHA PE=1 SV=2	LDHA_HUMAN	37 kDa	100%
333	Proteasome subunit alpha type-7 OS=Homo sapiens GN=PSMA7 PE=1 SV=1	PSA7_HUMAN	28 kDa	99%
334	Calpain-1 catalytic subunit OS=Homo sapiens GN=CAPN1 PE=1 SV=1	CAN1_HUMAN	82 kDa	100%
335	Endoplasmic reticulum resident protein 29 OS=Homo sapiens GN=ERP29 PE=1 SV=4	ERP29_HUMAN	29 kDa	100%
336	Putative RNA-binding protein Luc7-like 2 OS=Homo sapiens GN=LUC7L2 PE=1 SV=2	LC7L2_HUMAN	47 kDa	100%
337	Proteasome activator complex subunit 1 OS=Homo sapiens GN=PSME1 PE=1 SV=1	PSME1_HUMAN	29 kDa	100%
338	N-alpha-acetyltransferase 10 OS=Homo sapiens GN=NAA10 PE=1 SV=1	NAA10_HUMAN	26 kDa	100%
339	60S ribosomal export protein NMD3 OS=Homo sapiens GN=NMD3 PE=1 SV=1	NMD3_HUMAN	58 kDa	100%
340	Eukaryotic translation initiation factor 3 subunit C OS=Homo sapiens GN=EIF3C PE=1 SV=1	EIF3C_HUMAN	105 kDa	99%
341	Mitochondrial import receptor subunit TOM20 homolog OS=Homo sapiens GN=TOMM20 PE=1 SV=1	TOM20_HUMAN	16 kDa	99%

342	Ran-specific GTPase-activating protein OS=Homo sapiens GN=RANBP1 PE=1 SV=1	RANG_HUMAN	23 kDa	100%
343	Nucleobindin-1 OS=Homo sapiens GN=NUCB1 PE=1 SV=4	NUCB1_HUMAN	54 kDa	100%
344	RNA-binding protein 28 OS=Homo sapiens GN=RBM28 PE=1 SV=3	RBM28_HUMAN	86 kDa	100%
345	Exportin-1 OS=Homo sapiens GN=XPO1 PE=1 SV=1	XPO1_HUMAN	123 kDa	100%
346	Calumenin OS=Homo sapiens GN=CALU PE=1 SV=2	CALU_HUMAN	37 kDa	99%
347	Heterogeneous nuclear ribonucleoprotein U OS=Homo sapiens GN=HNRNPU PE=1 SV=6	HNRPU_HUMAN	91 kDa	98%
348	Ribonuclease inhibitor OS=Homo sapiens GN=RNH1 PE=1 SV=2	RINI_HUMAN	50 kDa	99%
349	Small nuclear ribonucleoprotein E OS=Homo sapiens GN=SNRPE PE=1 SV=1	RUXE_HUMAN	11 kDa	99%
350	Peroxiredoxin-5, mitochondrial OS=Homo sapiens GN=PRDX5 PE=1 SV=4	PRDX5_HUMAN	22 kDa	99%
351	LDLR chaperone MESD OS=Homo sapiens GN=MESDC2 PE=1 SV=2	MESD_HUMAN	26 kDa	100%
352	Heterogeneous nuclear ribonucleoprotein H2 OS=Homo sapiens GN=HNRNPH2 PE=1 SV=1	HNRH2_HUMAN	49 kDa	18%
353	Hepatoma-derived growth factor-related protein 2 OS=Homo sapiens GN=HDGFRP2 PE=1 SV=1	HDGR2_HUMAN	74 kDa	100%
354	Perilipin-3 OS=Homo sapiens GN=PLIN3 PE=1 SV=3	PLIN3_HUMAN	47 kDa	99%
355	Sorting nexin-5 OS=Homo sapiens GN=SNX5 PE=1 SV=1	SNX5_HUMAN	47 kDa	100%
356	Endoplasmic reticulum resident protein 44 OS=Homo sapiens GN=ERP44 PE=1 SV=1	ERP44_HUMAN	47 kDa	100%
357	Aspartyl/asparaginyl beta-hydroxylase OS=Homo sapiens GN=ASPH PE=1 SV=3	ASPH_HUMAN	86 kDa	99%
358	Cytoskeleton-associated protein 4 OS=Homo sapiens GN=CKAP4 PE=1 SV=2	CKAP4_HUMAN	66 kDa	100%
359	RNA-binding motif protein, X chromosome OS=Homo sapiens GN=RBMX PE=1 SV=3	RBMX_HUMAN (+1)	42 kDa	99%
360	ATP-dependent RNA helicase DDX19A OS=Homo sapiens GN=DDX19A PE=1 SV=1	DD19A_HUMAN	54 kDa	99%
361	PCTP-like protein OS=Homo sapiens GN=STARD10 PE=1 SV=2	PCTL_HUMAN	33 kDa	100%
362	Platelet-activating factor acetylhydrolase IB subunit alpha OS=Homo sapiens GN=PAFAH1B1 PE=1 SV=2	LIS1_HUMAN	47 kDa	99%
363	Glucose-6-phosphate 1-dehydrogenase OS=Homo sapiens GN=G6PD PE=1 SV=4	G6PD_HUMAN	59 kDa	99%
364	40S ribosomal protein S17-like OS=Homo sapiens GN=RPS17L PE=2 SV=1	RS17L_HUMAN (+1)	16 kDa	100%
365	Nucleolar RNA helicase 2 OS=Homo sapiens GN=DDX21 PE=1 SV=5	DDX21_HUMAN	87 kDa	100%
366	Scaffold attachment factor B1 OS=Homo sapiens GN=SAFB PE=1 SV=4	SAFB1_HUMAN	103 kDa	100%
367	Vesicular integral-membrane protein VIP36 OS=Homo sapiens GN=LMAN2 PE=1 SV=1	LMAN2_HUMAN	40 kDa	100%

368	Alanine--tRNA ligase, cytoplasmic OS=Homo sapiens GN=AARS PE=1 SV=2	SYAC_HUMAN	107 kDa	100%
369	Protein furry homolog-like OS=Homo sapiens GN=FRYL PE=1 SV=2	FRYL_HUMAN	340 kDa	100%
370	Hsp90 co-chaperone Cdc37 OS=Homo sapiens GN=CDC37 PE=1 SV=1	CDC37_HUMAN	44 kDa	100%
371	Sorting nexin-6 OS=Homo sapiens GN=SNX6 PE=1 SV=1	SNX6_HUMAN	47 kDa	99%
372	26S proteasome non-ATPase regulatory subunit 1 OS=Homo sapiens GN=PSMD1 PE=1 SV=2	PSMD1_HUMAN	106 kDa	100%
373	Transportin-1 OS=Homo sapiens GN=TNPO1 PE=1 SV=2	TNPO1_HUMAN	102 kDa	100%
374	Transportin-2 OS=Homo sapiens GN=TNPO2 PE=1 SV=3	TNPO2_HUMAN	101 kDa	100%
375	Signal recognition particle receptor subunit beta OS=Homo sapiens GN=SRPRB PE=1 SV=3	SRPRB_HUMAN	30 kDa	99%
376	Vacuolar protein sorting-associated protein 26A OS=Homo sapiens GN=VPS26A PE=1 SV=2	VP26A_HUMAN	38 kDa	78%
377	Valine--tRNA ligase OS=Homo sapiens GN=VARS PE=1 SV=4	SYVC_HUMAN	140 kDa	100%
378	A-kinase anchor protein 8-like OS=Homo sapiens GN=AKAP8L PE=1 SV=3	AKP8L_HUMAN	72 kDa	99%
379	BH3-interacting domain death agonist OS=Homo sapiens GN=BID PE=1 SV=1	BID_HUMAN	22 kDa	99%
380	Heterogeneous nuclear ribonucleoprotein F OS=Homo sapiens GN=HNRNPF PE=1 SV=3	HNRPF_HUMAN	46 kDa	99%
381	Prohibitin-2 OS=Homo sapiens GN=PHB2 PE=1 SV=2	PHB2_HUMAN	33 kDa	99%
382	Adenylyl cyclase-associated protein 1 OS=Homo sapiens GN=CAP1 PE=1 SV=5	CAP1_HUMAN	52 kDa	100%
383	Heterogeneous nuclear ribonucleoprotein H OS=Homo sapiens GN=HNRNPH1 PE=1 SV=4	HNRH1_HUMAN	49 kDa	100%
384	Eukaryotic translation initiation factor 3 subunit J OS=Homo sapiens GN=EIF3J PE=1 SV=2	EIF3J_HUMAN	29 kDa	100%
385	Inorganic pyrophosphatase OS=Homo sapiens GN=PPA1 PE=1 SV=2	IPYR_HUMAN	33 kDa	100%
386	tRNA (cytosine(34)-C(5))-methyltransferase OS=Homo sapiens GN=NSUN2 PE=1 SV=2	NSUN2_HUMAN	86 kDa	100%
387	Histone H2A.V OS=Homo sapiens GN=H2AFV PE=1 SV=3	H2AV_HUMAN (+1)	14 kDa	99%
388	Dihydrolipoyllysine-residue succinyltransferase component of 2-oxoglutarate dehydrogenase complex, mitochondrial OS=Homo sapiens GN=DLST PE=1 SV=4	ODO2_HUMAN	49 kDa	99%
389	39S ribosomal protein L41, mitochondrial OS=Homo sapiens GN=MRPL41 PE=1 SV=1	RM41_HUMAN	15 kDa	99%
390	Small glutamine-rich tetratricopeptide repeat-containing protein alpha OS=Homo sapiens GN=SGTA PE=1 SV=1	SGTA_HUMAN	34 kDa	99%
391	Superoxide dismutase [Cu-Zn] OS=Homo sapiens GN=SOD1 PE=1 SV=2	SODC_HUMAN	16 kDa	99%
392	Filamin-C OS=Homo sapiens GN=FLNC PE=1 SV=3	FLNC_HUMAN	291 kDa	94%

393	Chloride intracellular channel protein 1 OS=Homo sapiens GN=CLIC1 PE=1 SV=4	CLIC1_HUMAN	27 kDa	100%
394	Zinc finger CCHC domain-containing protein 8 OS=Homo sapiens GN=ZCCHC8 PE=1 SV=2	ZCHC8_HUMAN	79 kDa	100%
395	14-3-3 protein beta/alpha OS=Homo sapiens GN=YWHAB PE=1 SV=3	1433B_HUMAN	28 kDa	100%
396	Rho-related GTP-binding protein RhoB OS=Homo sapiens GN=RHOB PE=1 SV=1	RHOB_HUMAN	22 kDa	98%
397	Histone H4 OS=Homo sapiens GN=HIST1H4A PE=1 SV=2	H4_HUMAN	11 kDa	99%
398	Peptidyl-prolyl cis-trans isomerase FKBP5 OS=Homo sapiens GN=FKBP5 PE=1 SV=2	FKBP5_HUMAN	51 kDa	99%
399	ERO1-like protein alpha OS=Homo sapiens GN=ERO1L PE=1 SV=2	ERO1A_HUMAN	54 kDa	99%
400	Glutamine--fructose-6-phosphate aminotransferase [isomerizing] 1 OS=Homo sapiens GN=GFPT1 PE=1 SV=3	GFPT1_HUMAN	79 kDa	99%
401	60S ribosomal protein L14 OS=Homo sapiens GN=RPL14 PE=1 SV=4	RL14_HUMAN	23 kDa	99%
402	Serine/threonine-protein kinase OSR1 OS=Homo sapiens GN=OXSR1 PE=1 SV=1	OXSR1_HUMAN	58 kDa	99%
403	Chromobox protein homolog 1 OS=Homo sapiens GN=CBX1 PE=1 SV=1	CBX1_HUMAN	21 kDa	99%
404	ATP-citrate synthase OS=Homo sapiens GN=ACLY PE=1 SV=3	ACLY_HUMAN	121 kDa	80%
405	Epidermal growth factor receptor kinase substrate 8-like protein 1 OS=Homo sapiens GN=EPS8L1 PE=1 SV=3	ES8L1_HUMAN	80 kDa	100%
406	Dynamin-like 120 kDa protein, mitochondrial OS=Homo sapiens GN=OPA1 PE=1 SV=3	OPA1_HUMAN	112 kDa	100%
407	Nucleosome assembly protein 1-like 4 OS=Homo sapiens GN=NAP1L4 PE=1 SV=1	NP1L4_HUMAN	43 kDa	99%
408	Annexin A1 OS=Homo sapiens GN=ANXA1 PE=1 SV=2	ANXA1_HUMAN	39 kDa	99%
409	AP-1 complex subunit sigma-1A OS=Homo sapiens GN=AP1S1 PE=1 SV=1	AP1S1_HUMAN	19 kDa	99%
410	Copper chaperone for superoxide dismutase OS=Homo sapiens GN=CCS PE=1 SV=1	CCS_HUMAN	29 kDa	99%
411	A-kinase anchor protein 8 OS=Homo sapiens GN=AKAP8 PE=1 SV=1	AKAP8_HUMAN	76 kDa	62%
412	Acyl-CoA desaturase OS=Homo sapiens GN=SCD PE=1 SV=2	ACOD_HUMAN	42 kDa	99%
413	Membrane-associated progesterone receptor component 1 OS=Homo sapiens GN=PGRMC1 PE=1 SV=3	PGRC1_HUMAN	22 kDa	99%
414	Double-stranded RNA-binding protein Staufen homolog 1 OS=Homo sapiens GN=STAU1 PE=1 SV=2	STAU1_HUMAN	63 kDa	99%
415	Dynactin subunit 2 OS=Homo sapiens GN=DCTN2 PE=1 SV=4	DCTN2_HUMAN	44 kDa	99%
416	26S proteasome non-ATPase regulatory subunit 4 OS=Homo sapiens GN=PSMD4 PE=1 SV=1	PSMD4_HUMAN	41 kDa	99%
417	Histone-binding protein RBBP4 OS=Homo sapiens GN=RBBP4 PE=1 SV=3	RBBP4_HUMAN	48 kDa	99%
418	60S ribosomal protein L35a OS=Homo sapiens GN=RPL35A PE=1 SV=2	RL35A_HUMAN	13 kDa	99%

419	Cytosol aminopeptidase OS=Homo sapiens GN=LAP3 PE=1 SV=3	AMPL_HUMAN	56 kDa	100%
420	Large proline-rich protein BAG6 OS=Homo sapiens GN=BAG6 PE=1 SV=2	BAG6_HUMAN	119 kDa	99%
421	Coatomer subunit zeta-1 OS=Homo sapiens GN=COPZ1 PE=1 SV=1	COPZ1_HUMAN	20 kDa	99%
422	Importin-7 OS=Homo sapiens GN=IPO7 PE=1 SV=1	IPO7_HUMAN	120 kDa	99%
423	Rab11 family-interacting protein 1 OS=Homo sapiens GN=RAB11FIP1 PE=1 SV=2	RFIP1_HUMAN	137 kDa	99%
424	Opioid growth factor receptor OS=Homo sapiens GN=OGFR PE=1 SV=3	OGFR_HUMAN	73 kDa	99%
425	Prolactin regulatory element-binding protein OS=Homo sapiens GN=PREB PE=1 SV=2	PREB_HUMAN	45 kDa	99%
426	Asparagine--tRNA ligase, cytoplasmic OS=Homo sapiens GN=NARS PE=1 SV=1	SYNC_HUMAN	63 kDa	99%
427	Transketolase OS=Homo sapiens GN=TKT PE=1 SV=3	TKT_HUMAN	68 kDa	99%
428	Vacuolar protein sorting-associated protein 37A OS=Homo sapiens GN=VPS37A PE=1 SV=1	VP37A_HUMAN	44 kDa	99%
429	AN1-type zinc finger protein 6 OS=Homo sapiens GN=ZFAND6 PE=1 SV=2	ZFAN6_HUMAN	23 kDa	99%
430	40S ribosomal protein S21 OS=Homo sapiens GN=RPS21 PE=1 SV=1	RS21_HUMAN	9 kDa	99%

Table S2 Identified a total of 469 proteins in set two

#	Identified Proteins	Accession Number	Molecular Weight	Probability
1	Neuroblast differentiation-associated protein AHNAK OS=Homo sapiens GN=AHNAK PE=1 SV=2	AHNK_HUMAN	629 kDa	100%
2	Cluster of Heat shock protein HSP 90-alpha OS=Homo sapiens GN=HSP90AA1 PE=1 SV=5 (HS90A_HUMAN)	HS90A_HUMAN [3]	85 kDa	100%
2.1	Heat shock protein HSP 90-alpha OS=Homo sapiens GN=HSP90AA1 PE=1 SV=5	HS90A_HUMAN	85 kDa	100%
2.2	Heat shock protein HSP 90-beta OS=Homo sapiens GN=HSP90AB1 PE=1 SV=4	HS90B_HUMAN	83 kDa	100%
3	Keratin, type II cytoskeletal 8 OS=Homo sapiens GN=KRT8 PE=1 SV=7	K2C8_HUMAN	54 kDa	100%
4	Tubulin alpha-1A chain OS=Homo sapiens GN=TUBA1A PE=1 SV=1	TBA1A_HUMAN (+2)	50 kDa	100%
5	Heat shock 70 kDa protein 1A/1B OS=Homo sapiens GN=HSPA1A PE=1 SV=5	HSP71_HUMAN	70 kDa	100%
6	Keratin, type I cytoskeletal 18 OS=Homo sapiens GN=KRT18 PE=1 SV=2	K1C18_HUMAN	48 kDa	100%
7	Cluster of Alpha-actinin-4 OS=Homo sapiens GN=ACTN4 PE=1 SV=2 (ACTN4_HUMAN)	ACTN4_HUMAN [2]	105 kDa	100%
7.1	Alpha-actinin-4 OS=Homo sapiens GN=ACTN4 PE=1 SV=2	ACTN4_HUMAN	105 kDa	100%
7.2	Alpha-actinin-1 OS=Homo sapiens GN=ACTN1 PE=1 SV=2	ACTN1_HUMAN	103 kDa	100%
8	Cluster of Tubulin beta chain OS=Homo sapiens GN=TUBB PE=1 SV=2 (TBB5_HUMAN)	TBB5_HUMAN [6]	50 kDa	100%
8.1	Tubulin beta chain OS=Homo sapiens GN=TUBB PE=1 SV=2	TBB5_HUMAN	50 kDa	100%
8.3	Tubulin beta-3 chain OS=Homo sapiens GN=TUBB3 PE=1 SV=2	TBB3_HUMAN	50 kDa	81%
8.4	Tubulin beta-4B chain OS=Homo sapiens GN=TUBB4B PE=1 SV=1	TBB4B_HUMAN	50 kDa	76%
9	Cluster of Actin, cytoplasmic 1 OS=Homo sapiens GN=ACTB PE=1 SV=1 (ACTB_HUMAN)	ACTB_HUMAN [6]	42 kDa	100%
9.1	Actin, cytoplasmic 1 OS=Homo sapiens GN=ACTB PE=1 SV=1	ACTB_HUMAN (+1)	42 kDa	100%
9.2	Actin, aortic smooth muscle OS=Homo sapiens GN=ACTA2 PE=1 SV=1	ACTA_HUMAN (+2)	42 kDa	99%
9.3	POTE ankyrin domain family member F OS=Homo sapiens GN=POTEF PE=1 SV=2	POTEF_HUMAN	121 kDa	94%
10	Cluster of Myosin-9 OS=Homo sapiens GN=MYH9 PE=1 SV=4 (MYH9_HUMAN)	MYH9_HUMAN [2]	227 kDa	100%
10.1	Myosin-9 OS=Homo sapiens GN=MYH9 PE=1 SV=4	MYH9_HUMAN	227 kDa	100%
11	14-3-3 protein zeta/delta OS=Homo sapiens GN=YWHAZ PE=1 SV=1	1433Z_HUMAN	28 kDa	100%
12	Cluster of Clathrin heavy chain 1 OS=Homo sapiens GN=CLTC PE=1 SV=5 (CLH1_HUMAN)	CLH1_HUMAN [2]	192 kDa	100%

12.1	Clathrin heavy chain 1 OS=Homo sapiens GN=CLTC PE=1 SV=5	CLH1_HUMAN	192 kDa	100%
13	Protein SEC13 homolog OS=Homo sapiens GN=SEC13 PE=1 SV=3	SEC13_HUMAN	36 kDa	99%
14	Cluster of Tropomyosin alpha-4 chain OS=Homo sapiens GN=TPM4 PE=1 SV=3 (TPM4_HUMAN)	TPM4_HUMAN [2]	29 kDa	100%
14.1	Tropomyosin alpha-4 chain OS=Homo sapiens GN=TPM4 PE=1 SV=3	TPM4_HUMAN	29 kDa	100%
14.2	Tropomyosin alpha-3 chain OS=Homo sapiens GN=TPM3 PE=1 SV=1	TPM3_HUMAN	33 kDa	100%
15	Proteasome subunit beta type-7 OS=Homo sapiens GN=PSMB7 PE=1 SV=1	PSB7_HUMAN	30 kDa	100%
16	Calnexin OS=Homo sapiens GN=CANX PE=1 SV=2	CALX_HUMAN	68 kDa	100%
17	Cluster of Filamin-A OS=Homo sapiens GN=FLNA PE=1 SV=4 (FLNA_HUMAN)	FLNA_HUMAN [3]	281 kDa	100%
17.1	Filamin-A OS=Homo sapiens GN=FLNA PE=1 SV=4	FLNA_HUMAN	281 kDa	100%
17.2	Filamin-B OS=Homo sapiens GN=FLNB PE=1 SV=2	FLNB_HUMAN	278 kDa	100%
18	78 kDa glucose-regulated protein OS=Homo sapiens GN=HSPA5 PE=1 SV=2	GRP78_HUMAN	72 kDa	100%
19	Cytoplasmic dynein 1 heavy chain 1 OS=Homo sapiens GN=DYNC1H1 PE=1 SV=5	DYHC1_HUMAN	532 kDa	100%
20	Profilin-1 OS=Homo sapiens GN=PFN1 PE=1 SV=2	PROF1_HUMAN	15 kDa	99%
21	60 kDa heat shock protein, mitochondrial OS=Homo sapiens GN=HSPD1 PE=1 SV=2	CH60_HUMAN	61 kDa	100%
22	Fructose-bisphosphate aldolase C OS=Homo sapiens GN=ALDOC PE=1 SV=2	ALDOC_HUMAN	39 kDa	100%
23	Alpha-enolase OS=Homo sapiens GN=ENO1 PE=1 SV=2	ENOA_HUMAN	47 kDa	100%
24	Nucleolin OS=Homo sapiens GN=NCL PE=1 SV=3	NUCL_HUMAN	77 kDa	100%
25	Dipeptidyl peptidase 3 OS=Homo sapiens GN=DPP3 PE=1 SV=2	DPP3_HUMAN	83 kDa	100%
26	Radixin OS=Homo sapiens GN=RDX PE=1 SV=1	RADI_HUMAN	69 kDa	99%
27	Nucleophosmin OS=Homo sapiens GN=NPM1 PE=1 SV=2	NPM_HUMAN	33 kDa	100%
28	Pyruvate kinase isozymes M1/M2 OS=Homo sapiens GN=PKM PE=1 SV=4	KPYM_HUMAN	58 kDa	100%
29	Annexin A2 OS=Homo sapiens GN=ANXA2 PE=1 SV=2	ANXA2_HUMAN	39 kDa	100%
30	Calreticulin OS=Homo sapiens GN=CALR PE=1 SV=1	CALR_HUMAN	48 kDa	100%
31	Cluster of Heat shock cognate 71 kDa protein OS=Homo sapiens GN=HSPA8 PE=1 SV=1 (HSP7C_HUMAN)	HSP7C_HUMAN	71 kDa	100%
31.1	Heat shock cognate 71 kDa protein OS=Homo sapiens GN=HSPA8 PE=1 SV=1	HSP7C_HUMAN	71 kDa	100%
32	Protein disulfide-isomerase OS=Homo sapiens GN=P4HB PE=1 SV=3	PDIA1_HUMAN	57 kDa	100%

33	Transcription intermediary factor 1-beta OS=Homo sapiens GN=TRIM28 PE=1 SV=5	TIF1B_HUMAN	89 kDa	100%
34	Myosin-14 OS=Homo sapiens GN=MYH14 PE=1 SV=2	MYH14_HUMAN	228 kDa	100%
35	Peptidyl-prolyl cis-trans isomerase FKBP4 OS=Homo sapiens GN=FKBP4 PE=1 SV=3	FKBP4_HUMAN	52 kDa	100%
36	Staphylococcal nuclease domain-containing protein 1 OS=Homo sapiens GN=SND1 PE=1 SV=1	SND1_HUMAN	102 kDa	100%
37	Protein disulfide-isomerase A4 OS=Homo sapiens GN=PDIA4 PE=1 SV=2	PDIA4_HUMAN	73 kDa	100%
38	DNA-dependent protein kinase catalytic subunit OS=Homo sapiens GN=PRKDC PE=1 SV=3	PRKDC_HUMAN	469 kDa	100%
39	PRA1 family protein 2 OS=Homo sapiens GN=PRAF2 PE=1 SV=1	PRAF2_HUMAN	19 kDa	99%
40	Partner of Y14 and mago OS=Homo sapiens GN=WIBG PE=1 SV=1	WIBG_HUMAN	23 kDa	98%
41	14-3-3 protein epsilon OS=Homo sapiens GN=YWHAE PE=1 SV=1	1433E_HUMAN	29 kDa	100%
42	Plectin OS=Homo sapiens GN=PLEC PE=1 SV=3	PLEC_HUMAN	532 kDa	100%
43	SUMO-activating enzyme subunit 2 OS=Homo sapiens GN=UBA2 PE=1 SV=2	SAE2_HUMAN	71 kDa	100%
44	SUMO-activating enzyme subunit 1 OS=Homo sapiens GN=SAE1 PE=1 SV=1	SAE1_HUMAN	38 kDa	100%
45	Calmodulin OS=Homo sapiens GN=CALM1 PE=1 SV=2	CALM_HUMAN	17 kDa	100%
46	Complement component 1 Q subcomponent-binding protein, mitochondrial OS=Homo sapiens GN=C1QBP PE=1 SV=1	C1QBP_HUMAN	31 kDa	100%
47	60S acidic ribosomal protein P2 OS=Homo sapiens GN=RPLP2 PE=1 SV=1	RLA2_HUMAN	12 kDa	100%
48	Thioredoxin OS=Homo sapiens GN=TXN PE=1 SV=3	THIO_HUMAN	12 kDa	100%
49	Splicing factor, proline- and glutamine-rich OS=Homo sapiens GN=SFPQ PE=1 SV=2	SFPQ_HUMAN	76 kDa	100%
50	Anterior gradient protein 2 homolog OS=Homo sapiens GN=AGR2 PE=1 SV=1	AGR2_HUMAN	20 kDa	99%
51	Ezrin OS=Homo sapiens GN=EZR PE=1 SV=4	EZRI_HUMAN	69 kDa	98%
52	Bcl-2-associated transcription factor 1 OS=Homo sapiens GN=BCLAF1 PE=1 SV=2	BCLF1_HUMAN	106 kDa	100%
53	Protein unc-45 homolog A OS=Homo sapiens GN=UNC45A PE=1 SV=1	UN45A_HUMAN	103 kDa	100%
54	Heterogeneous nuclear ribonucleoprotein Q OS=Homo sapiens GN=SYNCRIP PE=1 SV=2	HNRPQ_HUMAN	70 kDa	100%
55	40S ribosomal protein S3 OS=Homo sapiens GN=RPS3 PE=1 SV=2	RS3_HUMAN	27 kDa	100%
56	NEDD8 OS=Homo sapiens GN=NEDD8 PE=1 SV=1	NEDD8_HUMAN	9 kDa	100%
57	Chromobox protein homolog 3 OS=Homo sapiens GN=CBX3 PE=1 SV=4	CBX3_HUMAN	21 kDa	100%
58	Prelamin-A/C OS=Homo sapiens GN=LMNA PE=1 SV=1	LMNA_HUMAN	74 kDa	100%

59	Protein disulfide-isomerase A6 OS=Homo sapiens GN=PDIA6 PE=1 SV=1	PDIA6_HUMAN	48 kDa	100%
60	Spectrin alpha chain, non-erythrocytic 1 OS=Homo sapiens GN=SPTAN1 PE=1 SV=3	SPTN1_HUMAN	285 kDa	100%
61	Ras GTPase-activating-like protein IQGAP1 OS=Homo sapiens GN=IQGAP1 PE=1 SV=1	IQGA1_HUMAN	189 kDa	100%
62	X-ray repair cross-complementing protein 5 OS=Homo sapiens GN=XRCC5 PE=1 SV=3	XRCC5_HUMAN	83 kDa	100%
63	Y-box-binding protein 3 OS=Homo sapiens GN=YBX3 PE=1 SV=4	YBOX3_HUMAN	40 kDa	100%
64	Serine/arginine-rich splicing factor 5 OS=Homo sapiens GN=SRSF5 PE=1 SV=1	SRSF5_HUMAN	31 kDa	100%
65	26S proteasome non-ATPase regulatory subunit 11 OS=Homo sapiens GN=PSMD11 PE=1 SV=3	PSD11_HUMAN	47 kDa	99%
66	Apolipoprotein B-100 OS=Homo sapiens GN=APOB PE=1 SV=2	APOB_HUMAN	516 kDa	99%
67	40S ribosomal protein S7 OS=Homo sapiens GN=RPS7 PE=1 SV=1	RS7_HUMAN	22 kDa	97%
68	Keratin, type I cytoskeletal 19 OS=Homo sapiens GN=KRT19 PE=1 SV=4	K1C19_HUMAN	44 kDa	100%
69	Myoferlin OS=Homo sapiens GN=MYOF PE=1 SV=1	MYOF_HUMAN	235 kDa	100%
70	Protein SET OS=Homo sapiens GN=SET PE=1 SV=3	SET_HUMAN	33 kDa	100%
71	Na(+)/H(+) exchange regulatory cofactor NHE-RF1 OS=Homo sapiens GN=SLC9A3R1 PE=1 SV=4	NHRF1_HUMAN	39 kDa	100%
72	Heterogeneous nuclear ribonucleoproteins A2/B1 OS=Homo sapiens GN=HNRNPA2B1 PE=1 SV=2	ROA2_HUMAN	37 kDa	100%
73	ATP synthase subunit alpha, mitochondrial OS=Homo sapiens GN=ATP5A1 PE=1 SV=1	ATPA_HUMAN	60 kDa	100%
74	Glyceraldehyde-3-phosphate dehydrogenase OS=Homo sapiens GN=GAPDH PE=1 SV=3	G3P_HUMAN	36 kDa	100%
75	Splicing factor 3B subunit 3 OS=Homo sapiens GN=SF3B3 PE=1 SV=4	SF3B3_HUMAN	136 kDa	100%
76	Cathepsin D OS=Homo sapiens GN=CTSD PE=1 SV=1	CATD_HUMAN	45 kDa	100%
77	NADP-dependent malic enzyme OS=Homo sapiens GN=ME1 PE=1 SV=1	MAOX_HUMAN	64 kDa	99%
78	Serine/arginine-rich splicing factor 2 OS=Homo sapiens GN=SRSF2 PE=1 SV=4	SRSF2_HUMAN	25 kDa	99%
79	6-phosphogluconate dehydrogenase, decarboxylating OS=Homo sapiens GN=PGD PE=1 SV=3	6PGD_HUMAN	53 kDa	100%
80	pre-rRNA processing protein FTSJ3 OS=Homo sapiens GN=FTSJ3 PE=1 SV=2	SPB1_HUMAN	97 kDa	100%
81	Cluster of Sarcoplasmic/endoplasmic reticulum calcium ATPase 2 OS=Homo sapiens GN=ATP2A2 PE=1 SV=1 (AT2A2_HUMAN)	AT2A2_HUMAN [2]	115 kDa	100%
81.1	Sarcoplasmic/endoplasmic reticulum calcium ATPase 2 OS=Homo sapiens GN=ATP2A2 PE=1 SV=1	AT2A2_HUMAN	115 kDa	100%
81.2	Sarcoplasmic/endoplasmic reticulum calcium ATPase 1 OS=Homo sapiens GN=ATP2A1 PE=1 SV=1	AT2A1_HUMAN	110 kDa	96%
82	Peroxiredoxin-6 OS=Homo sapiens GN=PRDX6 PE=1 SV=3	PRDX6_HUMAN	25 kDa	100%

83	Prefoldin subunit 4 OS=Homo sapiens GN=PFDN4 PE=1 SV=1	PFD4_HUMAN	15 kDa	100%
84	Ubiquitin-like modifier-activating enzyme 1 OS=Homo sapiens GN=UBA1 PE=1 SV=3	UBA1_HUMAN	118 kDa	100%
85	X-ray repair cross-complementing protein 6 OS=Homo sapiens GN=XRCC6 PE=1 SV=2	XRCC6_HUMAN	70 kDa	100%
86	Pre-mRNA-processing factor 40 homolog A OS=Homo sapiens GN=PRPF40A PE=1 SV=2	PR40A_HUMAN	109 kDa	100%
87	Thyroid hormone receptor-associated protein 3 OS=Homo sapiens GN=THRAP3 PE=1 SV=2	TR150_HUMAN	109 kDa	100%
88	40S ribosomal protein S14 OS=Homo sapiens GN=RPS14 PE=1 SV=3	RS14_HUMAN	16 kDa	100%
89	Src substrate cortactin OS=Homo sapiens GN=CTTN PE=1 SV=2	SRC8_HUMAN	62 kDa	100%
90	Triosephosphate isomerase OS=Homo sapiens GN=TPI1 PE=1 SV=3	TPIS_HUMAN	31 kDa	100%
91	60S ribosomal protein L4 OS=Homo sapiens GN=RPL4 PE=1 SV=5	RL4_HUMAN	48 kDa	100%
92	Proliferation-associated protein 2G4 OS=Homo sapiens GN=PA2G4 PE=1 SV=3	PA2G4_HUMAN	44 kDa	99%
93	Fructose-bisphosphate aldolase A OS=Homo sapiens GN=ALDOA PE=1 SV=2	ALDOA_HUMAN	39 kDa	100%
94	Cold shock domain-containing protein E1 OS=Homo sapiens GN=CSDE1 PE=1 SV=2	CSDE1_HUMAN	89 kDa	100%
95	Eukaryotic translation initiation factor 2 subunit 2 OS=Homo sapiens GN=EIF2S2 PE=1 SV=2	IF2B_HUMAN	38 kDa	100%
96	Transitional endoplasmic reticulum ATPase OS=Homo sapiens GN=VCP PE=1 SV=4	TERA_HUMAN	89 kDa	100%
97	Isocitrate dehydrogenase [NADP], mitochondrial OS=Homo sapiens GN=IDH2 PE=1 SV=2	IDHP_HUMAN	51 kDa	100%
98	Heterogeneous nuclear ribonucleoprotein A1-like 2 OS=Homo sapiens GN=HNRNPA1L2 PE=2 SV=2	RA1L2_HUMAN (+1)	34 kDa	100%
99	Heterogeneous nuclear ribonucleoprotein A3 OS=Homo sapiens GN=HNRNPA3 PE=1 SV=2	ROA3_HUMAN	40 kDa	100%
100	Stathmin OS=Homo sapiens GN=STMN1 PE=1 SV=3	STMN1_HUMAN	17 kDa	100%
101	Myristoylated alanine-rich C-kinase substrate OS=Homo sapiens GN=MARCKS PE=1 SV=4	MARCS_HUMAN	32 kDa	100%
102	Ubiquitin carboxyl-terminal hydrolase 14 OS=Homo sapiens GN=USP14 PE=1 SV=3	UBP14_HUMAN	56 kDa	100%
103	Gelsolin OS=Homo sapiens GN=GSN PE=1 SV=1	GELS_HUMAN	86 kDa	100%
104	Brain acid soluble protein 1 OS=Homo sapiens GN=BASP1 PE=1 SV=2	BASP1_HUMAN	23 kDa	100%
105	Superkiller viralicidic activity 2-like 2 OS=Homo sapiens GN=SKIV2L2 PE=1 SV=3	SK2L2_HUMAN	118 kDa	100%
106	Ubiquitin-conjugating enzyme E2 variant 1 OS=Homo sapiens GN=UBE2V1 PE=1 SV=2	UB2V1_HUMAN	16 kDa	100%
107	Vesicle-trafficking protein SEC22b OS=Homo sapiens GN=SEC22B PE=1 SV=4	SC22B_HUMAN	25 kDa	100%
108	Talin-1 OS=Homo sapiens GN=TLN1 PE=1 SV=3	TLN1_HUMAN	270 kDa	100%

109	60S ribosomal protein L10 OS=Homo sapiens GN=RPL10 PE=1 SV=4	RL10_HUMAN	25 kDa	99%
110	26S proteasome non-ATPase regulatory subunit 6 OS=Homo sapiens GN=PSMD6 PE=1 SV=1	PSMD6_HUMAN	46 kDa	100%
111	Hypoxia up-regulated protein 1 OS=Homo sapiens GN=HYOU1 PE=1 SV=1	HYOU1_HUMAN	111 kDa	100%
112	F-actin-capping protein subunit alpha-1 OS=Homo sapiens GN=CAPZA1 PE=1 SV=3	CAZA1_HUMAN	33 kDa	100%
113	Neutral alpha-glucosidase AB OS=Homo sapiens GN=GANAB PE=1 SV=3	GANAB_HUMAN	107 kDa	100%
114	Spectrin beta chain, non-erythrocytic 1 OS=Homo sapiens GN=SPTBN1 PE=1 SV=2	SPTB2_HUMAN	275 kDa	100%
115	C-1-tetrahydrofolate synthase, cytoplasmic OS=Homo sapiens GN=MTHFD1 PE=1 SV=3	C1TC_HUMAN	102 kDa	100%
116	Heat shock protein 105 kDa OS=Homo sapiens GN=HSPH1 PE=1 SV=1	HS105_HUMAN	97 kDa	100%
117	Elongation factor 2 OS=Homo sapiens GN=EEF2 PE=1 SV=4	EF2_HUMAN	95 kDa	100%
118	Cluster of Puromycin-sensitive aminopeptidase OS=Homo sapiens GN=NPEPPS PE=1 SV=2 (PSA_HUMAN)	PSA_HUMAN [2]	103 kDa	100%
118.1	Puromycin-sensitive aminopeptidase OS=Homo sapiens GN=NPEPPS PE=1 SV=2	PSA_HUMAN	103 kDa	95%
119	Heat shock protein beta-1 OS=Homo sapiens GN=HSPB1 PE=1 SV=2	HSPB1_HUMAN	23 kDa	100%
120	RNA-binding protein 39 OS=Homo sapiens GN=RBM39 PE=1 SV=2	RBM39_HUMAN	59 kDa	100%
121	Endoplasmic reticulum resident protein 29 OS=Homo sapiens GN=ERP29 PE=1 SV=4	ERP29_HUMAN	29 kDa	100%
122	Cluster of Phosphoglycerate kinase 1 OS=Homo sapiens GN=PGK1 PE=1 SV=3 (PGK1_HUMAN)	PGK1_HUMAN	45 kDa	100%
122.1	Phosphoglycerate kinase 1 OS=Homo sapiens GN=PGK1 PE=1 SV=3	PGK1_HUMAN	45 kDa	99%
122.2	Phosphoglycerate kinase 2 OS=Homo sapiens GN=PGK2 PE=1 SV=3	PGK2_HUMAN	45 kDa	11%
123	Serine/arginine repetitive matrix protein 2 OS=Homo sapiens GN=SRRM2 PE=1 SV=2	SRRM2_HUMAN	300 kDa	100%
124	Nuclear mitotic apparatus protein 1 OS=Homo sapiens GN=NUMA1 PE=1 SV=2	NUMA1_HUMAN	238 kDa	100%
125	C-Jun-amino-terminal kinase-interacting protein 4 OS=Homo sapiens GN=SPAG9 PE=1 SV=4	JIP4_HUMAN	146 kDa	100%
126	Putative tropomyosin alpha-3 chain-like protein OS=Homo sapiens PE=5 SV=2	TPM3L_HUMAN	26 kDa	100%
127	Proteasome subunit alpha type-5 OS=Homo sapiens GN=PSMA5 PE=1 SV=3	PSA5_HUMAN	26 kDa	100%
128	Leucine-rich PPR motif-containing protein, mitochondrial OS=Homo sapiens GN=LRPPRC PE=1 SV=3	LPPRC_HUMAN	158 kDa	100%
129	ATP synthase subunit gamma, mitochondrial OS=Homo sapiens GN=ATP5C1 PE=1 SV=1	ATPG_HUMAN	33 kDa	100%
130	Elongation factor Ts, mitochondrial OS=Homo sapiens GN=TSFM PE=1 SV=2	EFTS_HUMAN	35 kDa	100%
131	Vacuolar protein sorting-associated protein 13C OS=Homo sapiens GN=VPS13C PE=1 SV=1	VP13C_HUMAN	422 kDa	99%

132	BolA-like protein 2 OS=Homo sapiens GN=BOLA2 PE=1 SV=1	BOLA2_HUMAN	10 kDa	99%
133	Ubiquitin-60S ribosomal protein L40 OS=Homo sapiens GN=UBA52 PE=1 SV=2	RL40_HUMAN (+3)	15 kDa	99%
134	Fragile X mental retardation syndrome-related protein 1 OS=Homo sapiens GN=FXR1 PE=1 SV=3	FXR1_HUMAN	70 kDa	100%
135	Lamin-B1 OS=Homo sapiens GN=LMNB1 PE=1 SV=2	LMNB1_HUMAN	66 kDa	100%
136	4F2 cell-surface antigen heavy chain OS=Homo sapiens GN=SLC3A2 PE=1 SV=3	4F2_HUMAN	68 kDa	100%
137	Bifunctional purine biosynthesis protein PURH OS=Homo sapiens GN=ATIC PE=1 SV=3	PUR9_HUMAN	65 kDa	100%
138	Eukaryotic translation initiation factor 3 subunit B OS=Homo sapiens GN=EIF3B PE=1 SV=3	EIF3B_HUMAN	92 kDa	100%
139	Eukaryotic translation initiation factor 4 gamma 1 OS=Homo sapiens GN=EIF4G1 PE=1 SV=4	IF4G1_HUMAN	175 kDa	100%
140	14-3-3 protein eta OS=Homo sapiens GN=YWHAH PE=1 SV=4	1433F_HUMAN	28 kDa	100%
141	Aspartate--tRNA ligase, cytoplasmic OS=Homo sapiens GN=DARS PE=1 SV=2	SYDC_HUMAN	57 kDa	100%
142	26S protease regulatory subunit 6A OS=Homo sapiens GN=PSMC3 PE=1 SV=3	PRS6A_HUMAN	49 kDa	100%
143	Putative pre-mRNA-splicing factor ATP-dependent RNA helicase DHX15 OS=Homo sapiens GN=DHX15 PE=1 SV=2	DHX15_HUMAN	91 kDa	100%
144	Heterogeneous nuclear ribonucleoproteins C1/C2 OS=Homo sapiens GN=HNRNPC PE=1 SV=4	HNRPC_HUMAN (+1)	34 kDa	100%
145	Heterogeneous nuclear ribonucleoprotein L OS=Homo sapiens GN=HNRNPL PE=1 SV=2	HNRPL_HUMAN	64 kDa	100%
146	Transcription activator BRG1 OS=Homo sapiens GN=SMARCA4 PE=1 SV=2	SMCA4_HUMAN	185 kDa	100%
147	Heat shock 70 kDa protein 4 OS=Homo sapiens GN=HSPA4 PE=1 SV=4	HSP74_HUMAN	94 kDa	100%
148	14-3-3 protein theta OS=Homo sapiens GN=YWHAQ PE=1 SV=1	1433T_HUMAN	28 kDa	100%
149	Glycogen phosphorylase, brain form OS=Homo sapiens GN=PYGB PE=1 SV=5	PYGB_HUMAN	97 kDa	100%
150	Peptidyl-prolyl cis-trans isomerase A OS=Homo sapiens GN=PPIA PE=1 SV=2	PPIA_HUMAN	18 kDa	100%
151	Cytospin-B OS=Homo sapiens GN=SPECC1 PE=1 SV=1	CYTSB_HUMAN	119 kDa	100%
152	Cluster of Keratin, type I cytoskeletal 14 OS=Homo sapiens GN=KRT14 PE=1 SV=4 (K1C14_HUMAN)	K1C14_HUMAN [3]	52 kDa	100%
152.1	Keratin, type I cytoskeletal 14 OS=Homo sapiens GN=KRT14 PE=1 SV=4	K1C14_HUMAN	52 kDa	75%
153	DNA-directed RNA polymerase II subunit RPB1 OS=Homo sapiens GN=POLR2A PE=1 SV=2	RPB1_HUMAN	217 kDa	100%
154	Prostaglandin E synthase 3 OS=Homo sapiens GN=PTGES3 PE=1 SV=1	TEBP_HUMAN	19 kDa	100%
155	Protein arginine N-methyltransferase 8 OS=Homo sapiens GN=PRMT8 PE=1 SV=2	ANM8_HUMAN	45 kDa	100%
156	Programmed cell death 6-interacting protein OS=Homo sapiens GN=PDCD6IP PE=1 SV=1	PDC6I_HUMAN	96 kDa	100%

157	Fructose-1,6-bisphosphatase 1 OS=Homo sapiens GN=FBP1 PE=1 SV=5	F16P1_HUMAN	37 kDa	100%
158	Proteasome activator complex subunit 2 OS=Homo sapiens GN=PSME2 PE=1 SV=4	PSME2_HUMAN	27 kDa	100%
159	40S ribosomal protein S18 OS=Homo sapiens GN=RPS18 PE=1 SV=3	RS18_HUMAN	18 kDa	99%
160	Eukaryotic translation initiation factor 3 subunit C OS=Homo sapiens GN=EIF3C PE=1 SV=1	EIF3C_HUMAN	105 kDa	99%
161	Glutaredoxin-3 OS=Homo sapiens GN=GLRX3 PE=1 SV=2	GLRX3_HUMAN	37 kDa	99%
162	GrpE protein homolog 1, mitochondrial OS=Homo sapiens GN=GRPEL1 PE=1 SV=2	GRPE1_HUMAN	24 kDa	99%
163	Heterogeneous nuclear ribonucleoprotein K OS=Homo sapiens GN=HNRNPK PE=1 SV=1	HNRPK_HUMAN	51 kDa	99%
164	Bifunctional 3'-phosphoadenosine 5'-phosphosulfate synthase 2 OS=Homo sapiens GN=PAPSS2 PE=1 SV=2	PAPS2_HUMAN	70 kDa	100%
165	Fatty acid synthase OS=Homo sapiens GN=FASN PE=1 SV=3	FAS_HUMAN	273 kDa	100%
166	14-3-3 protein sigma OS=Homo sapiens GN=SFN PE=1 SV=1	1433S_HUMAN	28 kDa	100%
167	Sodium/potassium-transporting ATPase subunit alpha-1 OS=Homo sapiens GN=ATP1A1 PE=1 SV=1	AT1A1_HUMAN	113 kDa	100%
168	Proteasome subunit alpha type-7 OS=Homo sapiens GN=PSMA7 PE=1 SV=1	PSA7_HUMAN	28 kDa	100%
169	PDZ domain-containing protein GIPC1 OS=Homo sapiens GN=GIPC1 PE=1 SV=2	GIPC1_HUMAN	36 kDa	100%
170	Dolichyl-diphosphooligosaccharide--protein glycosyltransferase subunit 1 OS=Homo sapiens GN=RPN1 PE=1 SV=1	RPN1_HUMAN	69 kDa	100%
171	Vigilin OS=Homo sapiens GN=HDLBP PE=1 SV=2	VIGLN_HUMAN	141 kDa	100%
172	Heterogeneous nuclear ribonucleoprotein D0 OS=Homo sapiens GN=HNRNPD PE=1 SV=1	HNRPD_HUMAN	38 kDa	100%
173	PRKC apoptosis WT1 regulator protein OS=Homo sapiens GN=PAWR PE=1 SV=1	PAWR_HUMAN	37 kDa	100%
174	Actin-related protein 3 OS=Homo sapiens GN=ACTR3 PE=1 SV=3	ARP3_HUMAN	47 kDa	100%
175	SWI/SNF-related matrix-associated actin-dependent regulator of chromatin subfamily A member 5 OS=Homo sapiens GN=SMARCA5 PE=1 SV=1	SMCA5_HUMAN	122 kDa	100%
176	Interleukin enhancer-binding factor 3 OS=Homo sapiens GN=ILF3 PE=1 SV=3	ILF3_HUMAN	95 kDa	100%
177	Cytochrome b-c1 complex subunit 7 OS=Homo sapiens GN=UQCRB PE=1 SV=2	QCR7_HUMAN	14 kDa	100%
178	DnaJ homolog subfamily B member 1 OS=Homo sapiens GN=DNAJB1 PE=1 SV=4	DNJB1_HUMAN	38 kDa	100%
179	Serine/arginine-rich splicing factor 1 OS=Homo sapiens GN=SRSF1 PE=1 SV=2	SRSF1_HUMAN	28 kDa	100%
180	Rho GDP-dissociation inhibitor 1 OS=Homo sapiens GN=ARHGDIA PE=1 SV=3	GDIR1_HUMAN	23 kDa	100%
181	Plasminogen activator inhibitor 1 RNA-binding protein OS=Homo sapiens GN=SERBP1 PE=1 SV=2	PAIRB_HUMAN	45 kDa	100%

182	LDLR chaperone MESD OS=Homo sapiens GN=MESDC2 PE=1 SV=2	MESD_HUMAN	26 kDa	100%
183	Heterogeneous nuclear ribonucleoprotein H2 OS=Homo sapiens GN=HNRNPH2 PE=1 SV=1	HNRH2_HUMAN	49 kDa	100%
184	Heterogeneous nuclear ribonucleoprotein R OS=Homo sapiens GN=HNRNPR PE=1 SV=1	HNRPR_HUMAN	71 kDa	100%
185	Lupus La protein OS=Homo sapiens GN=SSB PE=1 SV=2	LA_HUMAN	47 kDa	100%
186	Dihydrolipoyl dehydrogenase, mitochondrial OS=Homo sapiens GN=DLD PE=1 SV=2	DLDH_HUMAN	54 kDa	100%
187	40S ribosomal protein S3a OS=Homo sapiens GN=RPS3A PE=1 SV=2	RS3A_HUMAN	30 kDa	100%
188	Transportin-1 OS=Homo sapiens GN=TNPO1 PE=1 SV=2	TNPO1_HUMAN	102 kDa	100%
189	Tyrosine--tRNA ligase, cytoplasmic OS=Homo sapiens GN=YARS PE=1 SV=4	SYYC_HUMAN	59 kDa	100%
190	DNA-(apurinic or apyrimidinic site) lyase OS=Homo sapiens GN=APEX1 PE=1 SV=2	APEX1_HUMAN	36 kDa	100%
191	ATP-dependent RNA helicase A OS=Homo sapiens GN=DHX9 PE=1 SV=4	DHX9_HUMAN	141 kDa	100%
192	N-alpha-acetyltransferase 10 OS=Homo sapiens GN=NAA10 PE=1 SV=1	NAA10_HUMAN	26 kDa	100%
193	Uncharacterized protein C7orf50 OS=Homo sapiens GN=C7orf50 PE=1 SV=1	CG050_HUMAN	22 kDa	100%
194	Guanine nucleotide-binding protein subunit beta-2-like 1 OS=Homo sapiens GN=GNB2L1 PE=1 SV=3	GBLP_HUMAN	35 kDa	100%
195	Exportin-2 OS=Homo sapiens GN=CSE1L PE=1 SV=3	XPO2_HUMAN	110 kDa	100%
196	Hepatoma-derived growth factor OS=Homo sapiens GN=HDGF PE=1 SV=1	HDGF_HUMAN	27 kDa	100%
197	Protein FAM162A OS=Homo sapiens GN=FAM162A PE=1 SV=2	F162A_HUMAN	17 kDa	99%
198	Glucose-6-phosphate 1-dehydrogenase OS=Homo sapiens GN=G6PD PE=1 SV=4	G6PD_HUMAN	59 kDa	99%
199	Stomatin-like protein 2, mitochondrial OS=Homo sapiens GN=STOML2 PE=1 SV=1	STML2_HUMAN	39 kDa	99%
200	Tumor protein D54 OS=Homo sapiens GN=TPD52L2 PE=1 SV=2	TPD54_HUMAN	22 kDa	99%
201	Malate dehydrogenase, cytoplasmic OS=Homo sapiens GN=MDH1 PE=1 SV=4	MDHC_HUMAN	36 kDa	99%
202	DBIRD complex subunit KIAA1967 OS=Homo sapiens GN=KIAA1967 PE=1 SV=2	K1967_HUMAN	103 kDa	100%
203	UDP-glucose 6-dehydrogenase OS=Homo sapiens GN=UGDH PE=1 SV=1	UGDH_HUMAN	55 kDa	100%
204	14-3-3 protein gamma OS=Homo sapiens GN=YWHAG PE=1 SV=2	1433G_HUMAN	28 kDa	100%
205	40S ribosomal protein S17-like OS=Homo sapiens GN=RPS17L PE=2 SV=1	RS17L_HUMAN (+1)	16 kDa	100%
206	Calpastatin OS=Homo sapiens GN=CAST PE=1 SV=4	ICAL_HUMAN	77 kDa	100%
207	Aconitate hydratase, mitochondrial OS=Homo sapiens GN=ACO2 PE=1 SV=2	ACON_HUMAN	85 kDa	100%

208	Nuclear migration protein nudC OS=Homo sapiens GN=NUDC PE=1 SV=1	NUDC_HUMAN	38 kDa	100%
209	Calpain-1 catalytic subunit OS=Homo sapiens GN=CAPN1 PE=1 SV=1	CAN1_HUMAN	82 kDa	100%
210	Scaffold attachment factor B1 OS=Homo sapiens GN=SAFB PE=1 SV=4	SAFB1_HUMAN	103 kDa	100%
211	Peroxiredoxin-5, mitochondrial OS=Homo sapiens GN=PRDX5 PE=1 SV=4	PRDX5_HUMAN	22 kDa	100%
212	NADH dehydrogenase [ubiquinone] flavoprotein 2, mitochondrial OS=Homo sapiens GN=NDUFV2 PE=1 SV=2	NDUV2_HUMAN	27 kDa	100%
213	Putative RNA-binding protein Luc7-like 2 OS=Homo sapiens GN=LUC7L2 PE=1 SV=2	LC7L2_HUMAN	47 kDa	100%
214	Nucleobindin-1 OS=Homo sapiens GN=NUCB1 PE=1 SV=4	NUCB1_HUMAN	54 kDa	100%
215	Cingulin OS=Homo sapiens GN=CGN PE=1 SV=2	CING_HUMAN	136 kDa	100%
216	Cytoplasmic dynein 1 intermediate chain 2 OS=Homo sapiens GN=DYNC1I2 PE=1 SV=3	DC1I2_HUMAN	71 kDa	100%
217	Protein furry homolog-like OS=Homo sapiens GN=FRYL PE=1 SV=2	FRYL_HUMAN	340 kDa	100%
218	Sorting nexin-6 OS=Homo sapiens GN=SNX6 PE=1 SV=1	SNX6_HUMAN	47 kDa	100%
219	Serine/arginine-rich splicing factor 7 OS=Homo sapiens GN=SRSF7 PE=1 SV=1	SRSF7_HUMAN	27 kDa	100%
220	Epiplakin OS=Homo sapiens GN=EPPK1 PE=1 SV=2	EPIPL_HUMAN	556 kDa	100%
221	Tyrosine-protein phosphatase non-receptor type 1 OS=Homo sapiens GN=PTPN1 PE=1 SV=1	PTN1_HUMAN	50 kDa	100%
222	Inorganic pyrophosphatase OS=Homo sapiens GN=PPA1 PE=1 SV=2	IPYR_HUMAN	33 kDa	100%
223	1,2-dihydroxy-3-keto-5-methylthiopentene dioxygenase OS=Homo sapiens GN=ADI1 PE=1 SV=1	MTND_HUMAN	21 kDa	100%
224	Eukaryotic translation initiation factor 5A-1 OS=Homo sapiens GN=EIF5A PE=1 SV=2	IF5A1_HUMAN	17 kDa	100%
225	Spermatogenesis-defective protein 39 homolog OS=Homo sapiens GN=VIPAS39 PE=1 SV=1	SPE39_HUMAN	57 kDa	100%
226	RNA-binding motif protein, X chromosome OS=Homo sapiens GN=RBMX PE=1 SV=3	RBMX_HUMAN (+1)	42 kDa	100%
227	Hsp90 co-chaperone Cdc37 OS=Homo sapiens GN=CDC37 PE=1 SV=1	CDC37_HUMAN	44 kDa	100%
228	Nuclear autoantigenic sperm protein OS=Homo sapiens GN=NASP PE=1 SV=2	NASP_HUMAN	85 kDa	100%
229	Transportin-2 OS=Homo sapiens GN=TNPO2 PE=1 SV=3	TNPO2_HUMAN	101 kDa	100%
230	Heterogeneous nuclear ribonucleoprotein U OS=Homo sapiens GN=HNRNPU PE=1 SV=6	HNRPU_HUMAN	91 kDa	100%
231	60S ribosomal export protein NMD3 OS=Homo sapiens GN=NMD3 PE=1 SV=1	NMD3_HUMAN	58 kDa	100%
232	Prohibitin OS=Homo sapiens GN=PHB PE=1 SV=1	PHB_HUMAN	30 kDa	100%
233	Vacuolar protein sorting-associated protein 26A OS=Homo sapiens GN=VPS26A PE=1 SV=2	VP26A_HUMAN	38 kDa	100%

234	Perilipin-3 OS=Homo sapiens GN=PLIN3 PE=1 SV=3	PLIN3_HUMAN	47 kDa	100%
235	Calumenin OS=Homo sapiens GN=CALU PE=1 SV=2	CALU_HUMAN	37 kDa	100%
236	Spliceosome RNA helicase DDX39B OS=Homo sapiens GN=DDX39B PE=1 SV=1	DX39B_HUMAN	49 kDa	100%
237	Transcription elongation factor SPT6 OS=Homo sapiens GN=SUPT6H PE=1 SV=2	SPT6H_HUMAN	199 kDa	100%
238	Protein SON OS=Homo sapiens GN=SON PE=1 SV=4	SON_HUMAN	264 kDa	100%
239	Sorting nexin-5 OS=Homo sapiens GN=SNX5 PE=1 SV=1	SNX5_HUMAN	47 kDa	100%
240	40S ribosomal protein S6 OS=Homo sapiens GN=RPS6 PE=1 SV=1	RS6_HUMAN	29 kDa	100%
241	Serine/threonine-protein phosphatase 6 regulatory subunit 3 OS=Homo sapiens GN=PPP6R3 PE=1 SV=2	PP6R3_HUMAN	98 kDa	100%
242	tRNA-splicing ligase RtcB homolog OS=Homo sapiens GN=C22orf28 PE=1 SV=1	RTCB_HUMAN	55 kDa	100%
243	Protein TFG OS=Homo sapiens GN=TFG PE=1 SV=2	TFG_HUMAN	43 kDa	99%
244	Transaldolase OS=Homo sapiens GN=TALDO1 PE=1 SV=2	TALDO_HUMAN	38 kDa	99%
245	Gem-associated protein 5 OS=Homo sapiens GN=GEMIN5 PE=1 SV=3	GEMI5_HUMAN	169 kDa	99%
246	Protein HEXIM1 OS=Homo sapiens GN=HEXIM1 PE=1 SV=1	HEXI1_HUMAN	41 kDa	99%
247	Aspartyl/asparaginyl beta-hydroxylase OS=Homo sapiens GN=ASPH PE=1 SV=3	ASPH_HUMAN	86 kDa	99%
248	Calcium-regulated heat stable protein 1 OS=Homo sapiens GN=CARHSP1 PE=1 SV=2	CHSP1_HUMAN	16 kDa	99%
249	Ribonuclease inhibitor OS=Homo sapiens GN=RNH1 PE=1 SV=2	RINI_HUMAN	50 kDa	99%
250	60S ribosomal protein L23a OS=Homo sapiens GN=RPL23A PE=1 SV=1	RL23A_HUMAN	18 kDa	99%
251	Small nuclear ribonucleoprotein E OS=Homo sapiens GN=SNRPE PE=1 SV=1	RUXE_HUMAN	11 kDa	99%
252	Mitochondrial import receptor subunit TOM20 homolog OS=Homo sapiens GN=TOMM20 PE=1 SV=1	TOM20_HUMAN	16 kDa	99%
253	Prenylcysteine oxidase 1 OS=Homo sapiens GN=PCYOX1 PE=1 SV=3	PCYOX_HUMAN	57 kDa	99%
254	Treacle protein OS=Homo sapiens GN=TCOF1 PE=1 SV=3	TCOF_HUMAN	152 kDa	99%
255	Heat shock protein beta-8 OS=Homo sapiens GN=HSPB8 PE=1 SV=1	HSPB8_HUMAN	22 kDa	98%
256	Nucleolar RNA helicase 2 OS=Homo sapiens GN=DDX21 PE=1 SV=5	DDX21_HUMAN	87 kDa	100%
257	Ran-specific GTPase-activating protein OS=Homo sapiens GN=RANBP1 PE=1 SV=1	RANG_HUMAN	23 kDa	100%
258	Eukaryotic translation initiation factor 5 OS=Homo sapiens GN=EIF5 PE=1 SV=2	IF5_HUMAN	49 kDa	100%
259	Eukaryotic translation initiation factor 2 subunit 1 OS=Homo sapiens GN=EIF2S1 PE=1 SV=3	IF2A_HUMAN	36 kDa	100%

260	DNA topoisomerase 1 OS=Homo sapiens GN=TOP1 PE=1 SV=2	TOP1_HUMAN	91 kDa	100%
261	Adapter molecule crk OS=Homo sapiens GN=CRK PE=1 SV=2	CRK_HUMAN	34 kDa	100%
262	Trifunctional enzyme subunit alpha, mitochondrial OS=Homo sapiens GN=HADHA PE=1 SV=2	ECHA_HUMAN	83 kDa	100%
263	RNA-binding protein 28 OS=Homo sapiens GN=RBM28 PE=1 SV=3	RBM28_HUMAN	86 kDa	100%
264	Keratin, type II cytoskeletal 6A OS=Homo sapiens GN=KRT6A PE=1 SV=3	K2C6A_HUMAN	60 kDa	100%
265	Heterogeneous nuclear ribonucleoprotein H OS=Homo sapiens GN=HNRNPH1 PE=1 SV=4	HNRH1_HUMAN	49 kDa	100%
266	3-hydroxyisobutyrate dehydrogenase, mitochondrial OS=Homo sapiens GN=HIBADH PE=1 SV=2	3HIDH_HUMAN	35 kDa	100%
267	Endoplasmin OS=Homo sapiens GN=HSP90B1 PE=1 SV=1	ENPL_HUMAN	92 kDa	100%
268	tRNA methyltransferase 112 homolog OS=Homo sapiens GN=TRMT112 PE=1 SV=1	TR112_HUMAN	14 kDa	100%
269	RNA-binding protein with serine-rich domain 1 OS=Homo sapiens GN=RNPS1 PE=1 SV=1	RNPS1_HUMAN	34 kDa	100%
270	Reticulocalbin-2 OS=Homo sapiens GN=RCN2 PE=1 SV=1	RCN2_HUMAN	37 kDa	100%
271	26S proteasome non-ATPase regulatory subunit 1 OS=Homo sapiens GN=PSMD1 PE=1 SV=2	PSMD1_HUMAN	106 kDa	100%
272	Zinc finger CCHC domain-containing protein 8 OS=Homo sapiens GN=ZCCHC8 PE=1 SV=2	ZCHC8_HUMAN	79 kDa	100%
273	Cytoskeleton-associated protein 4 OS=Homo sapiens GN=CKAP4 PE=1 SV=2	CKAP4_HUMAN	66 kDa	100%
274	14-3-3 protein beta/alpha OS=Homo sapiens GN=YWHAB PE=1 SV=3	1433B_HUMAN	28 kDa	100%
275	Leucine-rich repeat-containing protein 59 OS=Homo sapiens GN=LRRC59 PE=1 SV=1	LRC59_HUMAN	35 kDa	100%
276	Septin-7 OS=Homo sapiens GN=SEPT7 PE=1 SV=2	SEPT7_HUMAN	51 kDa	100%
277	T-complex protein 1 subunit beta OS=Homo sapiens GN=CCT2 PE=1 SV=4	TCPB_HUMAN	57 kDa	100%
278	E3 ubiquitin-protein ligase UBR4 OS=Homo sapiens GN=UBR4 PE=1 SV=1	UBR4_HUMAN	574 kDa	100%
279	Signal recognition particle receptor subunit beta OS=Homo sapiens GN=SRPRB PE=1 SV=3	SRPRB_HUMAN	30 kDa	100%
280	Adenylyl cyclase-associated protein 1 OS=Homo sapiens GN=CAP1 PE=1 SV=5	CAP1_HUMAN	52 kDa	100%
281	Chloride intracellular channel protein 1 OS=Homo sapiens GN=CLIC1 PE=1 SV=4	CLIC1_HUMAN	27 kDa	100%
282	Microtubule-associated protein tau OS=Homo sapiens GN=MAPT PE=1 SV=5	TAU_HUMAN	79 kDa	100%
283	Galactokinase OS=Homo sapiens GN=GALK1 PE=1 SV=1	GALK1_HUMAN	42 kDa	100%
284	Catenin alpha-1 OS=Homo sapiens GN=CTNNA1 PE=1 SV=1	CTNA1_HUMAN	100 kDa	100%
285	60S ribosomal protein L27 OS=Homo sapiens GN=RPL27 PE=1 SV=2	RL27_HUMAN	16 kDa	100%

286	Eukaryotic translation initiation factor 3 subunit H OS=Homo sapiens GN=EIF3H PE=1 SV=1	EIF3H_HUMAN	40 kDa	100%
287	Protein HID1 OS=Homo sapiens GN=HID1 PE=1 SV=1	HID1_HUMAN	89 kDa	100%
288	AP-1 complex subunit beta-1 OS=Homo sapiens GN=AP1B1 PE=1 SV=2	AP1B1_HUMAN	105 kDa	99%
289	Methylcrotonoyl-CoA carboxylase subunit alpha, mitochondrial OS=Homo sapiens GN=MCCC1 PE=1 SV=3	MCCA_HUMAN	80 kDa	99%
290	Sec1 family domain-containing protein 1 OS=Homo sapiens GN=SCFD1 PE=1 SV=4	SCFD1_HUMAN	72 kDa	99%
291	RNA-binding protein 25 OS=Homo sapiens GN=RBM25 PE=1 SV=3	RBM25_HUMAN	100 kDa	99%
292	Eukaryotic translation initiation factor 6 OS=Homo sapiens GN=EIF6 PE=1 SV=1	IF6_HUMAN	27 kDa	99%
293	A-kinase anchor protein 8-like OS=Homo sapiens GN=AKAP8L PE=1 SV=3	AKP8L_HUMAN	72 kDa	99%
294	Actin-related protein 2 OS=Homo sapiens GN=ACTR2 PE=1 SV=1	ARP2_HUMAN	45 kDa	99%
295	Cytosolic acyl coenzyme A thioester hydrolase OS=Homo sapiens GN=ACOT7 PE=1 SV=3	BACH_HUMAN	42 kDa	99%
296	BH3-interacting domain death agonist OS=Homo sapiens GN=BID PE=1 SV=1	BID_HUMAN	22 kDa	99%
297	Chromobox protein homolog 1 OS=Homo sapiens GN=CBX1 PE=1 SV=1	CBX1_HUMAN	21 kDa	99%
298	ATP-dependent RNA helicase DDX19A OS=Homo sapiens GN=DDX19A PE=1 SV=1	DD19A_HUMAN (+1)	54 kDa	99%
299	Endoplasmic reticulum resident protein 44 OS=Homo sapiens GN=ERP44 PE=1 SV=1	ERP44_HUMAN	47 kDa	99%
300	Golgi to ER traffic protein 4 homolog OS=Homo sapiens GN=GET4 PE=1 SV=1	GET4_HUMAN	37 kDa	99%
301	Hepatoma-derived growth factor-related protein 2 OS=Homo sapiens GN=HDGFRP2 PE=1 SV=1	HDGR2_HUMAN	74 kDa	99%
302	L-lactate dehydrogenase A chain OS=Homo sapiens GN=LDHA PE=1 SV=2	LDHA_HUMAN	37 kDa	99%
303	Platelet-activating factor acetylhydrolase IB subunit alpha OS=Homo sapiens GN=PAFAH1B1 PE=1 SV=2	LIS1_HUMAN	47 kDa	99%
304	Vesicular integral-membrane protein VIP36 OS=Homo sapiens GN=LMAN2 PE=1 SV=1	LMAN2_HUMAN	40 kDa	99%
305	PCTP-like protein OS=Homo sapiens GN=STARD10 PE=1 SV=2	PCTL_HUMAN	33 kDa	99%
306	Prohibitin-2 OS=Homo sapiens GN=PHB2 PE=1 SV=2	PHB2_HUMAN	33 kDa	99%
307	Proteasome activator complex subunit 1 OS=Homo sapiens GN=PSME1 PE=1 SV=1	PSME1_HUMAN	29 kDa	99%
308	Splicing factor 3A subunit 3 OS=Homo sapiens GN=SF3A3 PE=1 SV=1	SF3A3_HUMAN	59 kDa	99%
309	Exportin-1 OS=Homo sapiens GN=XPO1 PE=1 SV=1	XPO1_HUMAN	123 kDa	99%
310	Synaptic vesicle membrane protein VAT-1 homolog OS=Homo sapiens GN=VAT1 PE=1 SV=2	VAT1_HUMAN	42 kDa	99%
311	Fragile X mental retardation syndrome-related protein 2 OS=Homo sapiens GN=FXR2 PE=1 SV=2	FXR2_HUMAN	74 kDa	99%

312	Elongation factor 1-beta OS=Homo sapiens GN=EEF1B2 PE=1 SV=3	EF1B_HUMAN	25 kDa	99%
313	Keratin, type II cytoskeletal 1 OS=Homo sapiens GN=KRT1 PE=1 SV=6	K2C1_HUMAN	66 kDa	99%
314	Coatomer subunit beta' OS=Homo sapiens GN=COPB2 PE=1 SV=2	COPB2_HUMAN	102 kDa	99%
315	Myosin regulatory light chain 12A OS=Homo sapiens GN=MYL12A PE=1 SV=2	ML12A_HUMAN (+1)	20 kDa	98%
316	Ras-related protein Rab-1A OS=Homo sapiens GN=RAB1A PE=1 SV=3	RAB1A_HUMAN	23 kDa	98%
317	Kynurenine 3-monooxygenase OS=Homo sapiens GN=KMO PE=1 SV=2	KMO_HUMAN	56 kDa	97%
318	ATP synthase subunit d, mitochondrial OS=Homo sapiens GN=ATP5H PE=1 SV=3	ATP5H_HUMAN	18 kDa	97%
319	Transformer-2 protein homolog beta OS=Homo sapiens GN=TRA2B PE=1 SV=1	TRA2B_HUMAN	34 kDa	97%
320	Density-regulated protein OS=Homo sapiens GN=DENR PE=1 SV=2	DENR_HUMAN	22 kDa	97%
321	Developmentally-regulated GTP-binding protein 1 OS=Homo sapiens GN=DRG1 PE=1 SV=1	DRG1_HUMAN	41 kDa	97%
322	Peroxiredoxin-1 OS=Homo sapiens GN=PRDX1 PE=1 SV=1	PRDX1_HUMAN (+1)	22 kDa	96%
323	Hematological and neurological expressed 1 protein OS=Homo sapiens GN=HN1 PE=1 SV=3	HN1_HUMAN	16 kDa	91%
324	ATP-citrate synthase OS=Homo sapiens GN=ACLY PE=1 SV=3	ACLY_HUMAN	121 kDa	100%
325	DnaJ homolog subfamily B member 11 OS=Homo sapiens GN=DNAJB11 PE=1 SV=1	DJB11_HUMAN	41 kDa	100%
326	Eukaryotic translation initiation factor 3 subunit G OS=Homo sapiens GN=EIF3G PE=1 SV=2	EIF3G_HUMAN	36 kDa	100%
327	Keratin, type I cytoskeletal 9 OS=Homo sapiens GN=KRT9 PE=1 SV=3	K1C9_HUMAN	62 kDa	100%
328	Rho-related GTP-binding protein RhoB OS=Homo sapiens GN=RHOB PE=1 SV=1	RHOB_HUMAN	22 kDa	100%
329	Splicing factor 3B subunit 1 OS=Homo sapiens GN=SF3B1 PE=1 SV=3	SF3B1_HUMAN	146 kDa	100%
330	Dynamin-like 120 kDa protein, mitochondrial OS=Homo sapiens GN=OPA1 PE=1 SV=3	OPA1_HUMAN	112 kDa	100%
331	Zinc finger protein 207 OS=Homo sapiens GN=ZNF207 PE=1 SV=1	ZN207_HUMAN	51 kDa	100%
332	Protein LSM14 homolog B OS=Homo sapiens GN=LSM14B PE=1 SV=1	LS14B_HUMAN	42 kDa	100%
333	Cytosol aminopeptidase OS=Homo sapiens GN=LAP3 PE=1 SV=3	AMPL_HUMAN	56 kDa	100%
334	Protein 4.1 OS=Homo sapiens GN=EPB41 PE=1 SV=4	41_HUMAN	97 kDa	100%
335	Periplakin OS=Homo sapiens GN=PPL PE=1 SV=4	PEPL_HUMAN	205 kDa	100%
336	Cytochrome b-c1 complex subunit Rieske, mitochondrial OS=Homo sapiens GN=UQCRFS1 PE=1 SV=2	UCRI_HUMAN	30 kDa	99%
337	tRNA (cytosine(34)-C(5))-methyltransferase OS=Homo sapiens GN=NSUN2 PE=1 SV=2	NSUN2_HUMAN	86 kDa	99%

338	Glucose-6-phosphate isomerase OS=Homo sapiens GN=GPI PE=1 SV=4	G6PI_HUMAN	63 kDa	99%
339	Heterogeneous nuclear ribonucleoprotein A/B OS=Homo sapiens GN=HNRNPAB PE=1 SV=2	ROAA_HUMAN	36 kDa	99%
340	Echinoderm microtubule-associated protein-like 4 OS=Homo sapiens GN=EML4 PE=1 SV=3	EMAL4_HUMAN	109 kDa	99%
341	Heme-binding protein 1 OS=Homo sapiens GN=HEBP1 PE=1 SV=1	HEBP1_HUMAN	21 kDa	99%
342	Proactivator polypeptide OS=Homo sapiens GN=PSAP PE=1 SV=2	SAP_HUMAN	58 kDa	99%
343	Synaptotagmin-like protein 2 OS=Homo sapiens GN=SYTL2 PE=1 SV=3	SYTL2_HUMAN	105 kDa	99%
344	Ubiquitin carboxyl-terminal hydrolase 5 OS=Homo sapiens GN=USP5 PE=1 SV=2	UBP5_HUMAN	96 kDa	99%
345	A-kinase anchor protein 8 OS=Homo sapiens GN=AKAP8 PE=1 SV=1	AKAP8_HUMAN	76 kDa	99%
346	Annexin A1 OS=Homo sapiens GN=ANXA1 PE=1 SV=2	ANXA1_HUMAN	39 kDa	99%
347	AP-1 complex subunit sigma-1A OS=Homo sapiens GN=AP1S1 PE=1 SV=1	AP1S1_HUMAN	19 kDa	99%
348	Large proline-rich protein BAG6 OS=Homo sapiens GN=BAG6 PE=1 SV=2	BAG6_HUMAN	119 kDa	99%
349	Copper chaperone for superoxide dismutase OS=Homo sapiens GN=CCS PE=1 SV=1	CCS_HUMAN	29 kDa	99%
350	Charged multivesicular body protein 2a OS=Homo sapiens GN=CHMP2A PE=1 SV=1	CHM2A_HUMAN	25 kDa	99%
351	Dynactin subunit 2 OS=Homo sapiens GN=DCTN2 PE=1 SV=4	DCTN2_HUMAN	44 kDa	99%
352	ERO1-like protein alpha OS=Homo sapiens GN=ERO1L PE=1 SV=2	ERO1A_HUMAN	54 kDa	99%
353	Redox-regulatory protein FAM213A OS=Homo sapiens GN=FAM213A PE=1 SV=3	F213A_HUMAN	26 kDa	99%
354	Peptidyl-prolyl cis-trans isomerase FKBP5 OS=Homo sapiens GN=FKBP5 PE=1 SV=2	FKBP5_HUMAN	51 kDa	99%
355	Glutamine--fructose-6-phosphate aminotransferase [isomerizing] 1 OS=Homo sapiens GN=GFPT1 PE=1 SV=3	GFPT1_HUMAN	79 kDa	99%
356	Histone H2A.V OS=Homo sapiens GN=H2AFV PE=1 SV=3	H2AV_HUMAN (+1)	14 kDa	99%
357	Histone H4 OS=Homo sapiens GN=HIST1H4A PE=1 SV=2	H4_HUMAN	11 kDa	99%
358	Heterogeneous nuclear ribonucleoprotein F OS=Homo sapiens GN=HNRNPF PE=1 SV=3	HNRPF_HUMAN	46 kDa	99%
359	Matrin-3 OS=Homo sapiens GN=MATR3 PE=1 SV=2	MATR3_HUMAN	95 kDa	99%
360	Nucleosome assembly protein 1-like 4 OS=Homo sapiens GN=NAP1L4 PE=1 SV=1	NP1L4_HUMAN (+1)	43 kDa	99%
361	Dihydrolipoyllysine-residue succinyltransferase component of 2-oxoglutarate dehydrogenase complex, mitochondrial OS=Homo sapiens GN=DLST PE=1 SV=4	ODO2_HUMAN	49 kDa	99%
362	Serine/threonine-protein kinase OSR1 OS=Homo sapiens GN=OXSR1 PE=1 SV=1	OXSR1_HUMAN	58 kDa	99%

363	26S proteasome non-ATPase regulatory subunit 4 OS=Homo sapiens GN=PSMD4 PE=1 SV=1	PSMD4_HUMAN	41 kDa	99%
364	Histone-binding protein RBBP4 OS=Homo sapiens GN=RBBP4 PE=1 SV=3	RBBP4_HUMAN	48 kDa	99%
365	Protein RER1 OS=Homo sapiens GN=RER1 PE=1 SV=1	RER1_HUMAN	23 kDa	99%
366	60S ribosomal protein L14 OS=Homo sapiens GN=RPL14 PE=1 SV=4	RL14_HUMAN	23 kDa	99%
367	39S ribosomal protein L41, mitochondrial OS=Homo sapiens GN=MRPL41 PE=1 SV=1	RM41_HUMAN	15 kDa	99%
368	40S ribosomal protein S8 OS=Homo sapiens GN=RPS8 PE=1 SV=2	RS8_HUMAN	24 kDa	99%
369	Small glutamine-rich tetratricopeptide repeat-containing protein alpha OS=Homo sapiens GN=SGTA PE=1 SV=1	SGTA_HUMAN	34 kDa	99%
370	Superoxide dismutase [Cu-Zn] OS=Homo sapiens GN=SOD1 PE=1 SV=2	SODC_HUMAN	16 kDa	99%
371	Valine--tRNA ligase OS=Homo sapiens GN=VARS PE=1 SV=4	SYVC_HUMAN	140 kDa	99%
372	Protein YIPF3 OS=Homo sapiens GN=YIPF3 PE=1 SV=1	YIPF3_HUMAN	38 kDa	99%
373	Scaffold attachment factor B2 OS=Homo sapiens GN=SAFB2 PE=1 SV=1	SAFB2_HUMAN	107 kDa	99%
374	Dolichyl-diphosphooligosaccharide--protein glycosyltransferase subunit 2 OS=Homo sapiens GN=RPN2 PE=1 SV=3	RPN2_HUMAN	69 kDa	99%
375	Twinfilin-1 OS=Homo sapiens GN=TWF1 PE=1 SV=3	TWF1_HUMAN	40 kDa	99%
376	UV excision repair protein RAD23 homolog B OS=Homo sapiens GN=RAD23B PE=1 SV=1	RD23B_HUMAN	43 kDa	99%
377	Spectrin beta chain, non-erythrocytic 4 OS=Homo sapiens GN=SPTBN4 PE=1 SV=2	SPTN4_HUMAN	289 kDa	99%
378	Transmembrane emp24 domain-containing protein 9 OS=Homo sapiens GN=TMED9 PE=1 SV=2	TMED9_HUMAN	27 kDa	99%
379	Desmoplakin OS=Homo sapiens GN=DSP PE=1 SV=3	DESP_HUMAN	332 kDa	99%
380	Hsc70-interacting protein OS=Homo sapiens GN=ST13 PE=1 SV=2	F10A1_HUMAN (+1)	41 kDa	99%
381	V-type proton ATPase catalytic subunit A OS=Homo sapiens GN=ATP6V1A PE=1 SV=2	VATA_HUMAN	68 kDa	99%
382	Stress-induced-phosphoprotein 1 OS=Homo sapiens GN=STIP1 PE=1 SV=1	STIP1_HUMAN	63 kDa	99%
383	Poly [ADP-ribose] polymerase 1 OS=Homo sapiens GN=PARP1 PE=1 SV=4	PARP1_HUMAN	113 kDa	98%
384	Cation-dependent mannose-6-phosphate receptor OS=Homo sapiens GN=M6PR PE=1 SV=1	MPRD_HUMAN	31 kDa	98%
385	M-phase phosphoprotein 8 OS=Homo sapiens GN=MPHOSPH8 PE=1 SV=2	MPP8_HUMAN	97 kDa	98%
386	Carbonic anhydrase 2 OS=Homo sapiens GN=CA2 PE=1 SV=2	CAH2_HUMAN	29 kDa	98%
387	Ras-related protein Rab-7a OS=Homo sapiens GN=RAB7A PE=1 SV=1	RAB7A_HUMAN	23 kDa	98%
388	N-acetyl-D-glucosamine kinase OS=Homo sapiens GN=NAGK PE=1 SV=4	NAGK_HUMAN	37 kDa	98%

389	Electron transfer flavoprotein subunit alpha, mitochondrial OS=Homo sapiens GN=ETFA PE=1 SV=1	ETFA_HUMAN	35 kDa	98%
390	Coatomer subunit epsilon OS=Homo sapiens GN=COPE PE=1 SV=3	COPE_HUMAN	34 kDa	98%
391	Eukaryotic translation initiation factor 4E OS=Homo sapiens GN=EIF4E PE=1 SV=2	IF4E_HUMAN	25 kDa	97%
392	Eukaryotic translation initiation factor 3 subunit J OS=Homo sapiens GN=EIF3J PE=1 SV=2	EIF3J_HUMAN	29 kDa	97%
393	N-acetyltransferase 10 OS=Homo sapiens GN=NAT10 PE=1 SV=2	NAT10_HUMAN	116 kDa	97%
394	Small nuclear ribonucleoprotein Sm D2 OS=Homo sapiens GN=SNRPD2 PE=1 SV=1	SMD2_HUMAN	14 kDa	97%
395	60S ribosomal protein L22 OS=Homo sapiens GN=RPL22 PE=1 SV=2	RL22_HUMAN	15 kDa	96%
396	60S ribosomal protein L19 OS=Homo sapiens GN=RPL19 PE=1 SV=1	RL19_HUMAN	23 kDa	96%
397	Tax1-binding protein 3 OS=Homo sapiens GN=TAX1BP3 PE=1 SV=2	TX1B3_HUMAN	14 kDa	96%
398	Sorting nexin-3 OS=Homo sapiens GN=SNX3 PE=1 SV=3	SNX3_HUMAN	19 kDa	96%
399	Proliferating cell nuclear antigen OS=Homo sapiens GN=PCNA PE=1 SV=1	PCNA_HUMAN	29 kDa	95%
400	Ornithine aminotransferase, mitochondrial OS=Homo sapiens GN=OAT PE=1 SV=1	OAT_HUMAN	49 kDa	95%
401	26S proteasome non-ATPase regulatory subunit 12 OS=Homo sapiens GN=PSMD12 PE=1 SV=3	PSD12_HUMAN	53 kDa	95%
402	Putative nascent polypeptide-associated complex subunit alpha-like protein OS=Homo sapiens GN=NACAP1 PE=5 SV=1	NACP1_HUMAN	23 kDa	95%
403	Apoptosis inhibitor 5 OS=Homo sapiens GN=API5 PE=1 SV=3	API5_HUMAN	59 kDa	95%
404	Serpin H1 OS=Homo sapiens GN=SERPINH1 PE=1 SV=2	SERPH_HUMAN	46 kDa	94%
405	UDP-N-acetylglucosamine transferase subunit ALG14 homolog OS=Homo sapiens GN=ALG14 PE=2 SV=1	ALG14_HUMAN	24 kDa	90%
406	Cofilin-1 OS=Homo sapiens GN=CFL1 PE=1 SV=3	COF1_HUMAN	19 kDa	99%
407	Coatomer subunit zeta-1 OS=Homo sapiens GN=COPZ1 PE=1 SV=1	COPZ1_HUMAN	20 kDa	99%
408	Cytochrome c oxidase subunit 5B, mitochondrial OS=Homo sapiens GN=COX5B PE=1 SV=2	COX5B_HUMAN	14 kDa	99%
409	ATP-dependent RNA helicase DDX1 OS=Homo sapiens GN=DDX1 PE=1 SV=2	DDX1_HUMAN	82 kDa	99%
410	Epidermal growth factor receptor kinase substrate 8-like protein 1 OS=Homo sapiens GN=EPS8L1 PE=1 SV=3	ES8L1_HUMAN	80 kDa	99%
411	RNA-binding protein FUS OS=Homo sapiens GN=FUS PE=1 SV=1	FUS_HUMAN	53 kDa	99%
412	Rab GDP dissociation inhibitor alpha OS=Homo sapiens GN=GDI1 PE=1 SV=2	GDIA_HUMAN	51 kDa	99%
413	Importin-7 OS=Homo sapiens GN=IPO7 PE=1 SV=1	IPO7_HUMAN	120 kDa	99%
414	Opioid growth factor receptor OS=Homo sapiens GN=OGFR PE=1 SV=3	OGFR_HUMAN	73 kDa	99%

415	Membrane-associated progesterone receptor component 1 OS=Homo sapiens GN=PGRMC1 PE=1 SV=3	PGRC1_HUMAN	22 kDa	99%
416	Prolactin regulatory element-binding protein OS=Homo sapiens GN=PREB PE=1 SV=2	PREB_HUMAN	45 kDa	99%
417	Rab11 family-interacting protein 1 OS=Homo sapiens GN=RAB11FIP1 PE=1 SV=2	RFIP1_HUMAN	137 kDa	99%
418	60S ribosomal protein L35a OS=Homo sapiens GN=RPL35A PE=1 SV=2	RL35A_HUMAN	13 kDa	99%
419	Lysosome membrane protein 2 OS=Homo sapiens GN=SCARB2 PE=1 SV=2	SCRB2_HUMAN	54 kDa	99%
420	Alpha-soluble NSF attachment protein OS=Homo sapiens GN=NAPA PE=1 SV=3	SNAA_HUMAN	33 kDa	99%
421	Asparagine--tRNA ligase, cytoplasmic OS=Homo sapiens GN=NARS PE=1 SV=1	SYNC_HUMAN	63 kDa	99%
422	Transketolase OS=Homo sapiens GN=TKT PE=1 SV=3	TKT_HUMAN	68 kDa	99%
423	E3 UFM1-protein ligase 1 OS=Homo sapiens GN=UFL1 PE=1 SV=2	UFL1_HUMAN	90 kDa	99%
424	Vacuolar protein sorting-associated protein 37A OS=Homo sapiens GN=VPS37A PE=1 SV=1	VP37A_HUMAN	44 kDa	99%
425	AN1-type zinc finger protein 6 OS=Homo sapiens GN=ZFAND6 PE=1 SV=2	ZFAN6_HUMAN	23 kDa	99%
426	Ubiquitin thioesterase OTUB1 OS=Homo sapiens GN=OTUB1 PE=1 SV=2	OTUB1_HUMAN	31 kDa	99%
427	ATP synthase-coupling factor 6, mitochondrial OS=Homo sapiens GN=ATP5J PE=1 SV=1	ATP5J_HUMAN	13 kDa	99%
428	Glia maturation factor beta OS=Homo sapiens GN=GMFB PE=1 SV=2	GMFB_HUMAN	17 kDa	99%
429	Coiled-coil and C2 domain-containing protein 1A OS=Homo sapiens GN=CC2D1A PE=1 SV=1	C2D1A_HUMAN	104 kDa	99%
430	Regulator of microtubule dynamics protein 1 OS=Homo sapiens GN=RMDN1 PE=1 SV=1	RMD1_HUMAN	36 kDa	98%
431	Kynureninase OS=Homo sapiens GN=KYNU PE=1 SV=1	KYNU_HUMAN	52 kDa	98%
432	Coiled-coil domain-containing protein 47 OS=Homo sapiens GN=CCDC47 PE=1 SV=1	CCD47_HUMAN	56 kDa	98%
433	Acyl-CoA desaturase OS=Homo sapiens GN=SCD PE=1 SV=2	ACOD_HUMAN	42 kDa	98%
434	60S ribosomal protein L31 OS=Homo sapiens GN=RPL31 PE=1 SV=1	RL31_HUMAN	14 kDa	98%
435	Protein arginine N-methyltransferase 5 OS=Homo sapiens GN=PRMT5 PE=1 SV=4	ANM5_HUMAN	73 kDa	98%
436	Glutathione S-transferase kappa 1 OS=Homo sapiens GN=GSTK1 PE=1 SV=3	GSTK1_HUMAN	25 kDa	98%
437	Survival of motor neuron-related-splicing factor 30 OS=Homo sapiens GN=SMNDC1 PE=1 SV=1	SPF30_HUMAN	27 kDa	98%
438	RuvB-like 1 OS=Homo sapiens GN=RUVBL1 PE=1 SV=1	RUVB1_HUMAN	50 kDa	97%
439	Basic leucine zipper and W2 domain-containing protein 2 OS=Homo sapiens GN=BZW2 PE=1 SV=1	BZW2_HUMAN	48 kDa	97%
440	ADP-ribosylation factor GTPase-activating protein 1 OS=Homo sapiens GN=ARFGAP1 PE=1 SV=2	ARFG1_HUMAN	45 kDa	97%

441	E3 ubiquitin/ISG15 ligase TRIM25 OS=Homo sapiens GN=TRIM25 PE=1 SV=2	TRI25_HUMAN	71 kDa	96%
442	FAD-linked sulfhydryl oxidase ALR OS=Homo sapiens GN=GFER PE=1 SV=2	ALR_HUMAN	23 kDa	96%
443	Synapse-associated protein 1 OS=Homo sapiens GN=SYAP1 PE=1 SV=1	SYAP1_HUMAN	40 kDa	96%
444	Single-stranded DNA-binding protein, mitochondrial OS=Homo sapiens GN=SSBP1 PE=1 SV=1	SSBP_HUMAN	17 kDa	96%
445	Septin-11 OS=Homo sapiens GN=SEPT11 PE=1 SV=3	SEP11_HUMAN	49 kDa	96%
446	Isoamyl acetate-hydrolyzing esterase 1 homolog OS=Homo sapiens GN=IAH1 PE=1 SV=1	IAH1_HUMAN	28 kDa	96%
447	Probable ATP-dependent RNA helicase DHX36 OS=Homo sapiens GN=DHX36 PE=1 SV=2	DHX36_HUMAN	115 kDa	96%
448	Nuclear cap-binding protein subunit 1 OS=Homo sapiens GN=NCBP1 PE=1 SV=1	NCBP1_HUMAN	92 kDa	96%
449	26S proteasome non-ATPase regulatory subunit 7 OS=Homo sapiens GN=PSMD7 PE=1 SV=2	PSD7_HUMAN	37 kDa	96%
450	High mobility group protein B1 OS=Homo sapiens GN=HMGB1 PE=1 SV=3	HMGB1_HUMAN	25 kDa	96%
451	Ras-related protein Rab-3D OS=Homo sapiens GN=RAB3D PE=1 SV=1	RAB3D_HUMAN	24 kDa	95%
452	N-acylneuraminate-9-phosphatase OS=Homo sapiens GN=NANP PE=1 SV=1	NANP_HUMAN	28 kDa	95%
453	Charged multivesicular body protein 7 OS=Homo sapiens GN=CHMP7 PE=1 SV=1	CHMP7_HUMAN	51 kDa	95%
454	SWI/SNF-related matrix-associated actin-dependent regulator of chromatin subfamily B member 1 OS=Homo sapiens GN=SMARCB1 PE=1 SV=2	SNF5_HUMAN	44 kDa	95%
455	Histone H1.4 OS=Homo sapiens GN=HIST1H1E PE=1 SV=2	H14_HUMAN (+1)	22 kDa	95%
456	Citrate synthase, mitochondrial OS=Homo sapiens GN=CS PE=1 SV=2	CISY_HUMAN	52 kDa	95%
457	Ras-related protein Rab-14 OS=Homo sapiens GN=RAB14 PE=1 SV=4	RAB14_HUMAN	24 kDa	95%
458	Serine/arginine repetitive matrix protein 1 OS=Homo sapiens GN=SRRM1 PE=1 SV=2	SRRM1_HUMAN	102 kDa	95%
459	Exocyst complex component 8 OS=Homo sapiens GN=EXOC8 PE=1 SV=2	EXOC8_HUMAN	82 kDa	94%
460	Tumor necrosis factor receptor type 1-associated DEATH domain protein OS=Homo sapiens GN=TRADD PE=1 SV=2	TRADD_HUMAN	34 kDa	94%
461	Vacuolar protein sorting-associated protein 4A OS=Homo sapiens GN=VPS4A PE=1 SV=1	VPS4A_HUMAN	49 kDa	94%
462	Dynamin-2 OS=Homo sapiens GN=DNM2 PE=1 SV=2	DYN2_HUMAN	98 kDa	93%
463	28S ribosomal protein S27, mitochondrial OS=Homo sapiens GN=MRPS27 PE=1 SV=3	RT27_HUMAN	48 kDa	93%
464	Nuclear factor of activated T-cells, cytoplasmic 3 OS=Homo sapiens GN=NFATC3 PE=1 SV=1	NFAC3_HUMAN	116 kDa	91%
465	Trans-acting T-cell-specific transcription factor GATA-3 OS=Homo sapiens GN=GATA3 PE=1 SV=1	GATA3_HUMAN	48 kDa	91%

466	Microtubule-associated serine/threonine-protein kinase 2 OS=Homo sapiens GN=MAST2 PE=1 SV=2	MAST2_HUMAN	196 kDa	90%
467	Epithelial cell adhesion molecule OS=Homo sapiens GN=EPCAM PE=1 SV=2	EPCAM_HUMAN	35 kDa	89%
468	Splicing factor 3B subunit 4 OS=Homo sapiens GN=SF3B4 PE=1 SV=1	SF3B4_HUMAN	44 kDa	89%
469	Cytosolic carboxypeptidase 2 OS=Homo sapiens GN=AGBL2 PE=2 SV=2	CBPC2_HUMAN	104 kDa	89%

Table S3 Differentially expressed proteins in Cos, Dehy, CD, and VOSL treated groups

Cos *vs* Control			Dehy *vs* Control			CD *vs* Control			VOSL *vs* Control		
ID	Type(s)	Log2 FC	ID	Type(s)	Log2 FC	ID	Type(s)	Log2 FC	ID	Type(s)	Log2 FC
ACTR3	other	-1.0	ACOT7	enzyme	0.8	AKAP8	other	-1.0	API5	other	-2.5
ALDOA	enzyme	-0.8	AGR2	other	-0.9	API5	other	-1.6	ALG14	enzyme	0.6
APEX1	enzyme	-0.8	AKAP8	other	1.0	ATP5J	transporter	-0.8	AKAP8	other	-0.7
ATIC	enzyme	0.6	API5	other	-2.6	BASP1	transcription regulator	0.6	ATP5C1	transporter	0.9
ATP5C1	transporter	-0.6	ATP5H	enzyme	-0.6	BZW2	translation regulator	0.7	ATP5J	transporter	-0.7
ATP5I	transporter	-0.6	BASP1	transcription regulator	0.6	CFL1	other	-0.6	BID	other	-0.8
BOLA2/BOLA2B	other	-0.6	BID	other	-0.6	CS	enzyme	-0.7	CAPN1	peptidase	-0.9
C1QBP	transcription regulator	-1.3	CCDC47	other	-0.6	DENR	other	-0.6	DNAJB11	other	1.1
CAPN1	peptidase	0.8	CFL1	other	-0.7	EIF4G1	translation regulator	-0.9	EIF4G1	translation regulator	-0.7
CARHSP1	other	-0.6	CHMP7	transporter	-0.9	EPCAM	other	-0.7	FLNB	other	-0.7
CCDC90B	other	-2.5	DPP3	peptidase	-0.8	FLNB	other	-0.8	FRYL	other	-0.7
CCT2	kinase	-1.3	EIF6	translation regulator	-1.0	GFER	enzyme	-1.3	GATA3	transcription regulator	-1.0
CD2BP2	other	0.6	EPB41	other	0.7	GNB2L1	enzyme	-0.9	GEMIN5	other	-0.8
CLIC1	ion channel	-1.7	EPS8L1	other	0.6	HNRNPU	transporter	-0.7	GNB2L1	enzyme	-0.8
CLTC	other	-2.4	EZR	other	-0.8	HSPH1	other	-0.9	HDLBP	transporter	-0.7
COL1A1	other	0.7	FAM213A	other	-1.6	KMO	enzyme	0.9	HIST1H1E	other	0.7
EIF6	translation regulator	1.6	FASN	enzyme	-0.7	KRT1	other	0.7	HSPB8	kinase	-0.6
FAM162A	other	0.6	GFER	enzyme	0.6	LUC7L2	other	0.8	KMO	enzyme	0.9
FAM21A/FAM21C	other	-1.8	GNB2L1	enzyme	-1.4	M6PR	transporter	0.8	LSM14B	other	-0.6
FXR1	other	-2.6	GSTK1	enzyme	0.9	MAPT	kinase	0.8	MARCKS	other	-0.8
GCN1L1	translation regulator	1.5	HID1	other	0.7	MATR3	other	-0.8	MATR3	other	-0.8
GEMIN5	other	1.2	HMGB1	transcription regulator	0.7	ME1	enzyme	0.8	MCCC1	enzyme	-0.6

Table S3 (Continued)

Cos *vs* Control			Dehy *vs* Control			CD *vs* Control			VOSL *vs* Control		
ID	Type(s)	Log2 FC	ID	Type(s)	Log2 FC	ID	Type(s)	Log2 FC	ID	Type(s)	Log2 FC
GFPT1	enzyme	0.9	HNRNPAB	enzyme	-0.8	NASP	other	-0.6	ME1	enzyme	1.0
HNRNPA1L2	other	-0.7	HNRNPC	other	-0.6	NMD3	other	-0.8	NASP	other	-0.7
HSP90AB1	enzyme	-1.2	HSPB1	other	0.8	PGK1	kinase	-0.6	NFATC3	transcription regulator	-1.2
HSPA4	other	-0.7	HSPH1	other	-0.8	PGK1	kinase	-0.6	NUCB1	other	0.7
HSPB8	kinase	1.2	LMNB1	other	-0.7	PGRMC1	transmembrane receptor	-0.7	PA2G4	transcription regulator	-0.7
HSPD1	enzyme	-1.0	LUC7L2	other	1.4	RANBP1	other	-0.7	PARP1	enzyme	0.7
HYOU1	other	-1.3	MAPT	kinase	0.9	RBM25	other	-0.6	PCNA	enzyme	0.7
ILF3	transcription regulator	-2.6	NAP1L4	other	-0.7	RMDN1	other	-0.6	RBM25	other	-0.8
KMO	enzyme	0.7	NFATC3	transcription regulator	0.8	RPN2	enzyme	-1.6	RPN2	enzyme	-2.1
KRT19	other	0.7	NUCB1	other	0.6	SAFB	other	-1.1	SAFB	other	-0.9
LAP3	peptidase	0.7	PCNA	enzyme	0.7	SCARB2	other	0.6	SF3B3	other	-0.6
LIG1	enzyme	1.2	PTGES3	enzyme	0.7	SMARCA4	transcription regulator	-0.6	SFN	other	0.8
LMAN2	transporter	1.3	RAB3D	enzyme	-0.9	TCOF1	transporter	-1.2	SND1	enzyme	-0.6
LMNA	other	1.5	RANBP1	other	-0.8	TFG	other	0.7	SON	other	-0.7
LMNB1	other	0.7	RDX	other	-0.6	TNPO2	transporter	-0.8	SRRM2	other	-0.9
LRRC59	other	0.6	RPN2	enzyme	-2.8	TRIM28	transcription regulator	-2.0	SSBP1	other	0.6
MAPT	kinase	1.3	SAFB	other	-0.7				STMN1	other	-0.6
MRPL41	other	-0.7	SEC13	transporter	0.6				TCOF1	transporter	-0.8
MYH10	other	-2.8	SFN	other	1.7				TFG	other	1.0
NCL	other	-0.6	SFPQ	other	-0.6				TPI1	enzyme	1.1
NEDD8	enzyme	-0.6	SMARCB1	transcription regulator	-1.1				TXN	enzyme	0.6
NPM1	transcription regulator	-1.0	SON	other	-0.6				UBR4	enzyme	-2.1

Table S3 (Continued)

Cos *vs* Control			Dehy *vs* Control			CD *vs* Control			VOSL *vs* Control		
ID	Type(s)	Log2 FC	ID	Type(s)	Log2 FC	ID	Type(s)	Log2 FC	ID	Type(s)	Log2 FC
OGFR	other	0.8	SPAG9	other	0.6				UQCRFS1	enzyme	-0.7
PAWR	transcription regulator	-0.6	SRRM1	other	0.8				VPS26A	transporter	-0.6
PFN1	other	-0.8	SSBP1	other	0.8				ZFAND6	other	-0.9
PGRMC1	transmembrane receptor	-2.4	STMN1	other	-0.8						
PYGB	enzyme	-1.0	SYNCRIP	other	-0.6						
RPS17/RPS17L	other	-1.0	TCOF1	transporter	-0.7						
RPS3	enzyme	-0.8	TFG	other	1.3						
SAE1	enzyme	-0.9	TOP1	enzyme	-0.7						
SAMM50	other	-1.0	TPI1	enzyme	1.1						
SEMA4C	other	-1.2	TUBB	other	-0.7						
SET	phosphatase	-0.9	TUBB	other	-0.7						
SF3B3	other	0.9	UQCRFS1	enzyme	-1.0						
SFN	other	2.0	XRCC6	enzyme	-0.6						
SRRT	other	1.0	YWHAB	transcription regulator	0.7						
SRSF5	other	-0.7	YWHAZ	enzyme	0.6						
STMN1	other	-2.7									
TPI1	enzyme	-0.6									
TRIM28	transcription regulator	-1.2									
TUBB	other	-1.0									
UQCRB	enzyme	0.7									
XRCC6	enzyme	1.2									

FC (fold change) = treated group / control group

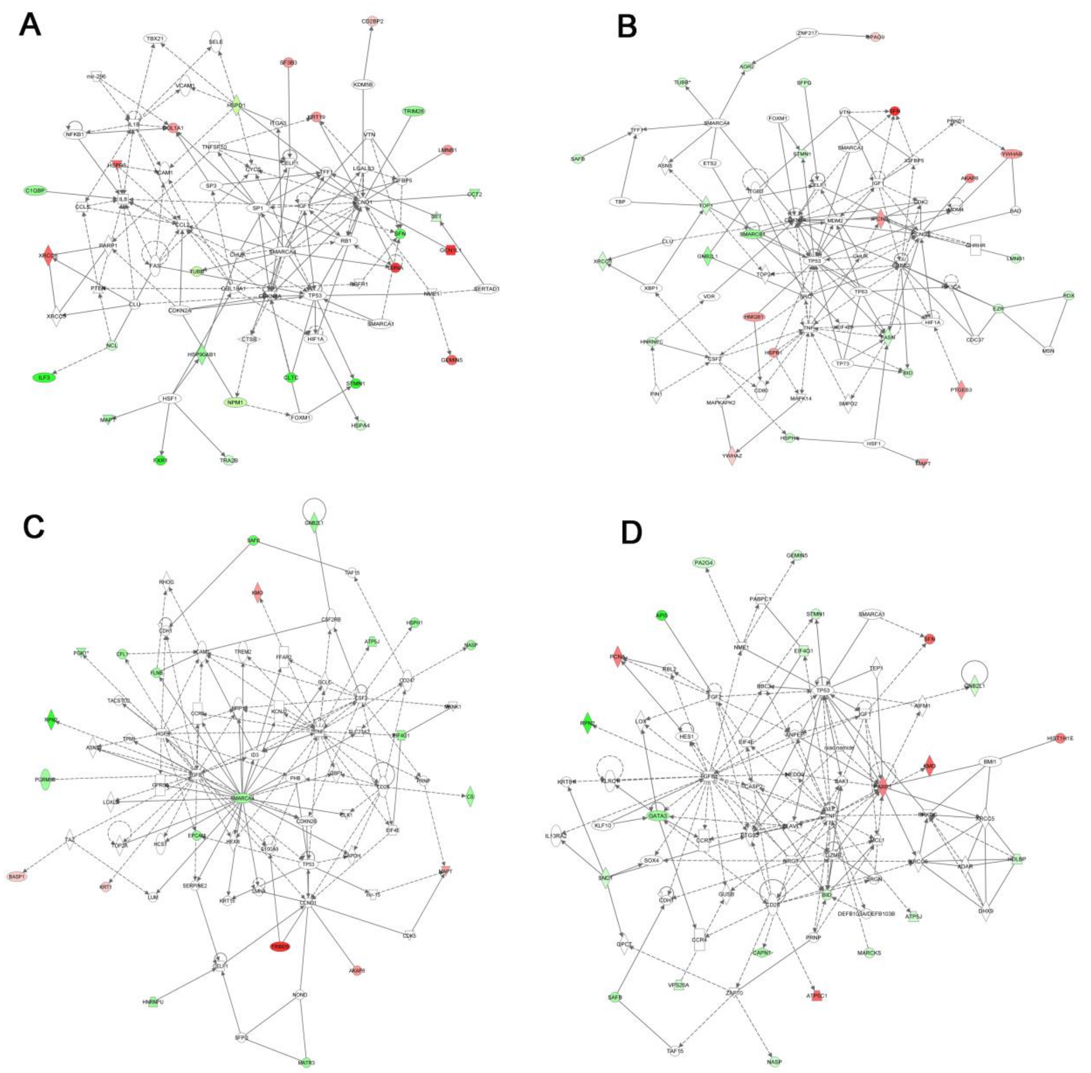

Figure S1. The interaction networks of differentially expressed proteins in (A) Cos-treated group; (B) Dehy-treated group; (C) CD-treated group; (D) VOSL-treated group. Red color represents “up-regulated”; Green color represents “down-regulated”.

The interaction networks of differentially expressed proteins in the Cos-treated group enriched 27 proteins (Fig. S1A), thereinto, 15 proteins, which were APEX1, C1QBP, COL1A1, FAM162A, FXR1, HSPB8, HSPD1, LMNA, MAPT, NPM1, PGRMC1, RPS3, SET, SFN, STMN1, involved in the physiologic functions of cell death and survival. The interaction networks of differentially expressed proteins in the Dehy-treated group enriched 26 proteins (Fig. S1B), of which there were 14 proteins, API5, BID, CFL1, EZR, FASN, GNB2L1, HMGB1, HSPB1, MAPT, SFN, SMARCB1, SON, TOP1 and YWHAZ, involved in the physiologic functions of cell death and survival. The interaction networks of differentially expressed proteins in the CD-treated group enriched 22 proteins (Fig. S1C), among which there were 4 proteins, MAPT, CFL1, FLNB and

SMARCA4, involved in the physiologic functions of cellular assembly, organization and cell cycle. The interaction networks in the VOSL-treated group enriched 23 differentially expressed proteins (Fig. S1D), in which there were 11 proteins, API5, BID, GNB2L1, HSPB8, PARP1, RBM25, SFN, SND1, SON, TXN and UBR4, involved in the physiologic functions of cell death and survival.

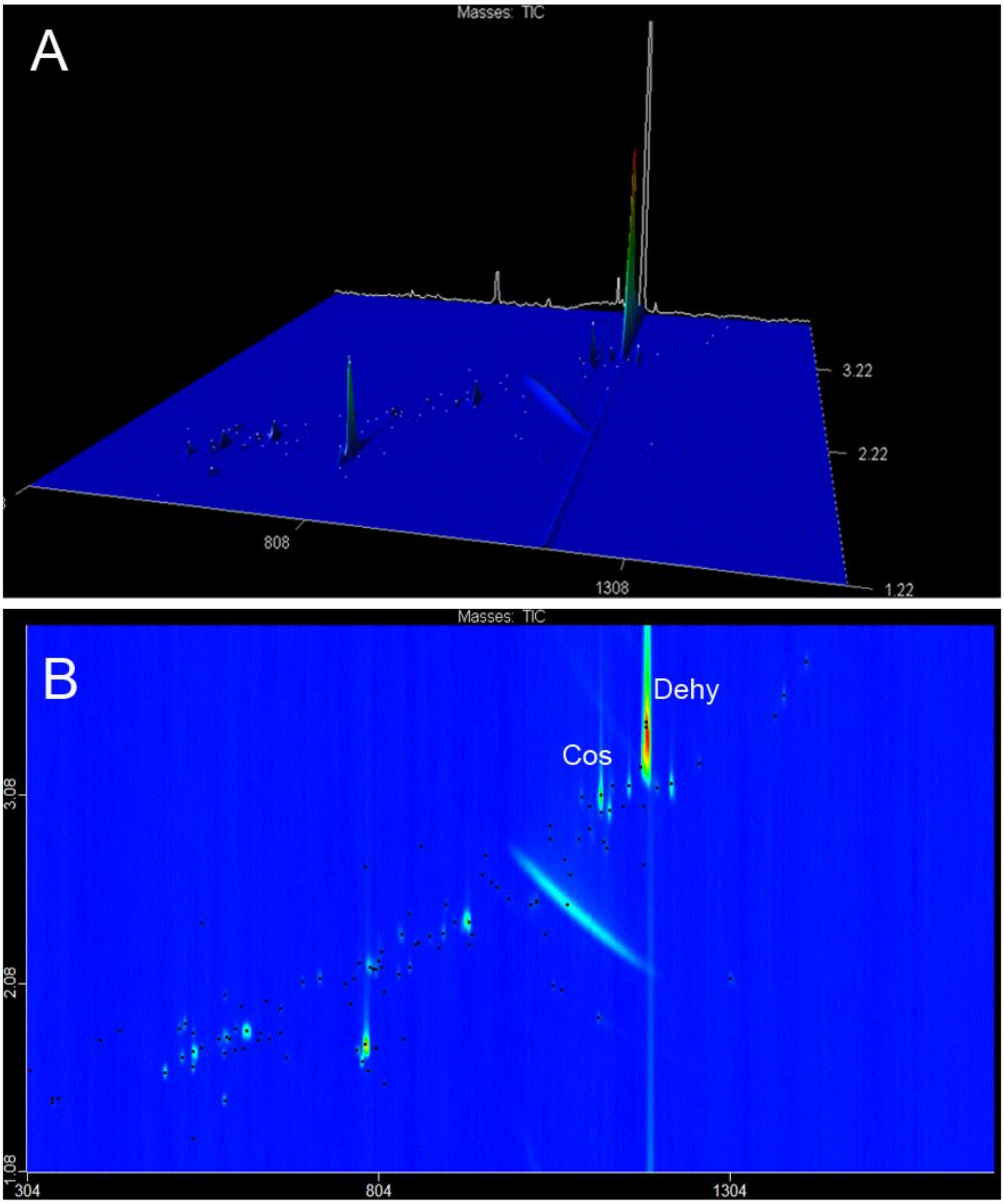

Figure S2. (A) The total ion chromatogram (three dimension, 3D) of VOSL by comprehensive two-dimensional gas chromatography-time-of-flight mass spectrometry (GC×GC–TOF /MS), (B) The total ion chromatogram (two dimension, 2D) of VOSL by GC×GC–TOF/MS.

Each 1 μL aliquot of the VOSL solution (0.1 mg/mL in hexane) was injected in splitless mode into a Pegasus 4D GC×GC–TOF/MS system (LECO Corporation, St Joseph, MI,USA). Separation and analysis were performed on a comprehensive 2D gas chromatography. The first dimension (1D) column was a non-polar DB-5 capillary column (30 m×250 μm I.D., J&W Scientific, Folsom, CA,

USA), coupled to a second dimension (2D) medium polarity DB-17 capillary column (1.8 m ×100 μm I.D., J&W Scientific, Folsom, CA, USA) by a glass union. The 2D column was held in a separate oven and maintained at a 5 ° C higher temperature than the 1D oven. The 2D separation run time was 4 s and was controlled with an internal cryogenic modulator. The analysis results revealed that the content of Cos and Dehy account for nearly 72% of VOSL. The details of constituents in VOSL were shown in **Table S4**

Table S4 The main constituents of VOSL

Compound	Retention time (s) (1D,2D)	Similarity	Unique Mass	Area %
Dehydro costuslactone	1184 , 3.470	942	230	50.46
Costunolide	1072 , 2.500	821	232	21.84
Cyclotrisiloxane, hexamethyl-	1848 , 0.850	922	207	10.40
9,12,15-Octadecatrienal	784 , 1.760	842	79	4.17
Oxacyclotetradeca-4,11-diyne	1120 , 3.080	763	91	3.15
β-Cyclocostunolide	1068 , 0.700	788	232	2.28
Cyclotetrasiloxane, octamethyl-	2016 , 0.870	844	281	1.04
Lanceol, cis	932 , 2.410	833	79	1.35
Dihydro costuslactone	1132 , 3.000	821	217	1.31
Alantolacton	1220 , 3.140	847	232	1.17

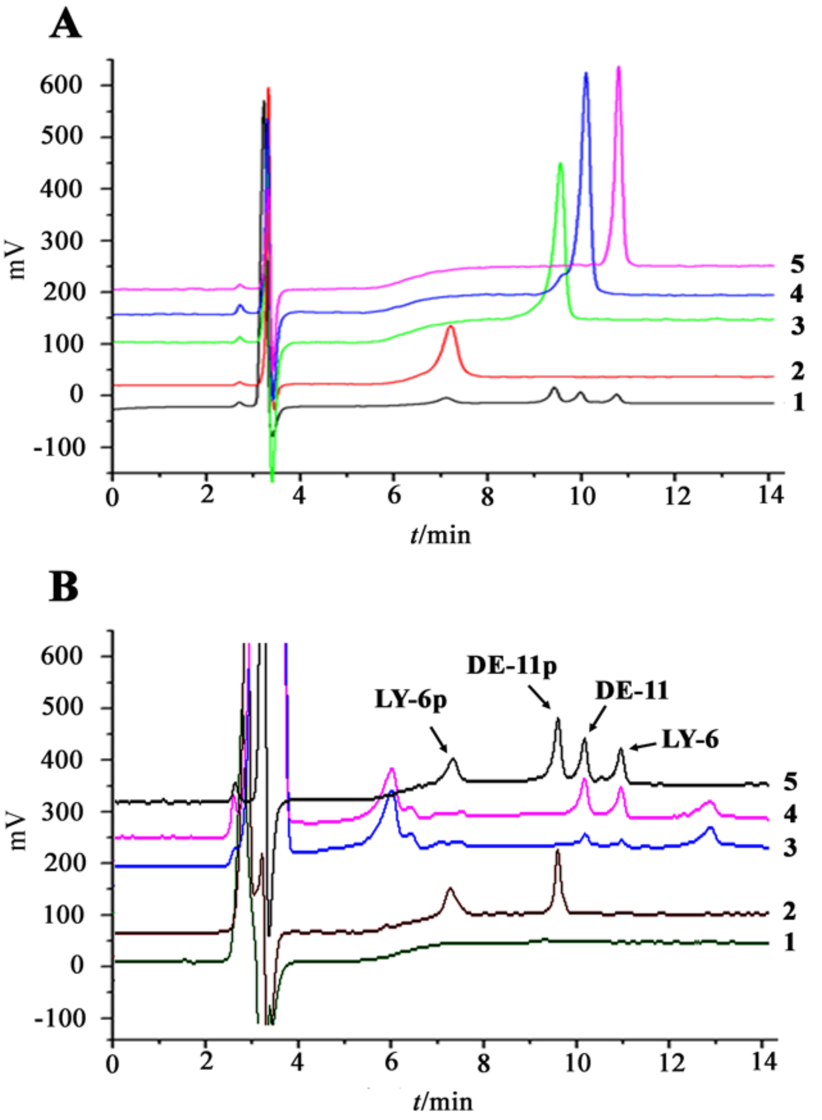

Figure S3. (A) Overlap chromatography of (1) Mixed peptide standards, (2) Phosphorylated peptide LY-6p, (3) Phosphorylated peptide DE-11p, (4) Nonphosphorylated peptide DE-11, and (5) Nonphosphorylated peptide LY-6 by high performance liquid chromatography coupled with ultraviolet detection (HPLC-UV); (B) Overlap chromatography of (1) Elution buffer Ⅱ, (2) Elution buffer Ⅰ, (3) Wash buffer Ⅱ, (4) Wash buffer Ⅰ after TiO_2 beads enrichment, and (5) Mixed peptide in loading buffer before TiO_2 beadsenrichment by HPLC-UV.

The commercial TiO_2 beads (GL Sciences, Tokyo, Japan) were used to enrichment of phosphorylated peptides according to the manufacturer's instructions, and the protocol was described in the method section "*TiO_2 enrichment and iTRAQ labeling*" of manuscript. A Shimadzu LC-20A series HPLC (Shimadzu, Kyoto, Japan) equipped with a binary pump and an

ultraviolet detector was used for the peptide samples. A TSKgel ODS-100V C18 column (5 μm, 250 × 4.6 mm, TOSOH, Japan) was used in the analysis. The detection wavelength was set at 220 nm. Solvent A (water, containing 0.05% TFA) and solvent B (ACN, containing 0.05% TFA) were used for gradient elution with the following program: 10–24% B for 0–14 min. The flow rate was 1.0 mL/min, the column temperature was 35 ℃, and the injection volume was 10 μL. The results revealed that the phosphorylated peptides (LY-6P and DE-11P) were in the elution buffer, and the nonphosphorylated peptides were in the wash buffer, therefore, the commercial TiO_2 beads had excellent enrichment efficiency for phosphorylated peptides. Using three concentration levels peptides to evaluated the recovery rates of phosphorylated peptides, and the details were shown in **Table S5**.

Table S5 Recovery of the peptide standards after TiO_2 beads enrichment

Concentration	Recovery (%) n=3			
(μg/mL)	LY-6p	DE-11p	LY-6	DE-11
10	83.7 ±3.5	75.8 ±1.9	0	0
50	86.2 ±2.7	77.0 ±2.3	0	0
100	88.4 ±2.1	81.4 ±3.8	0	0

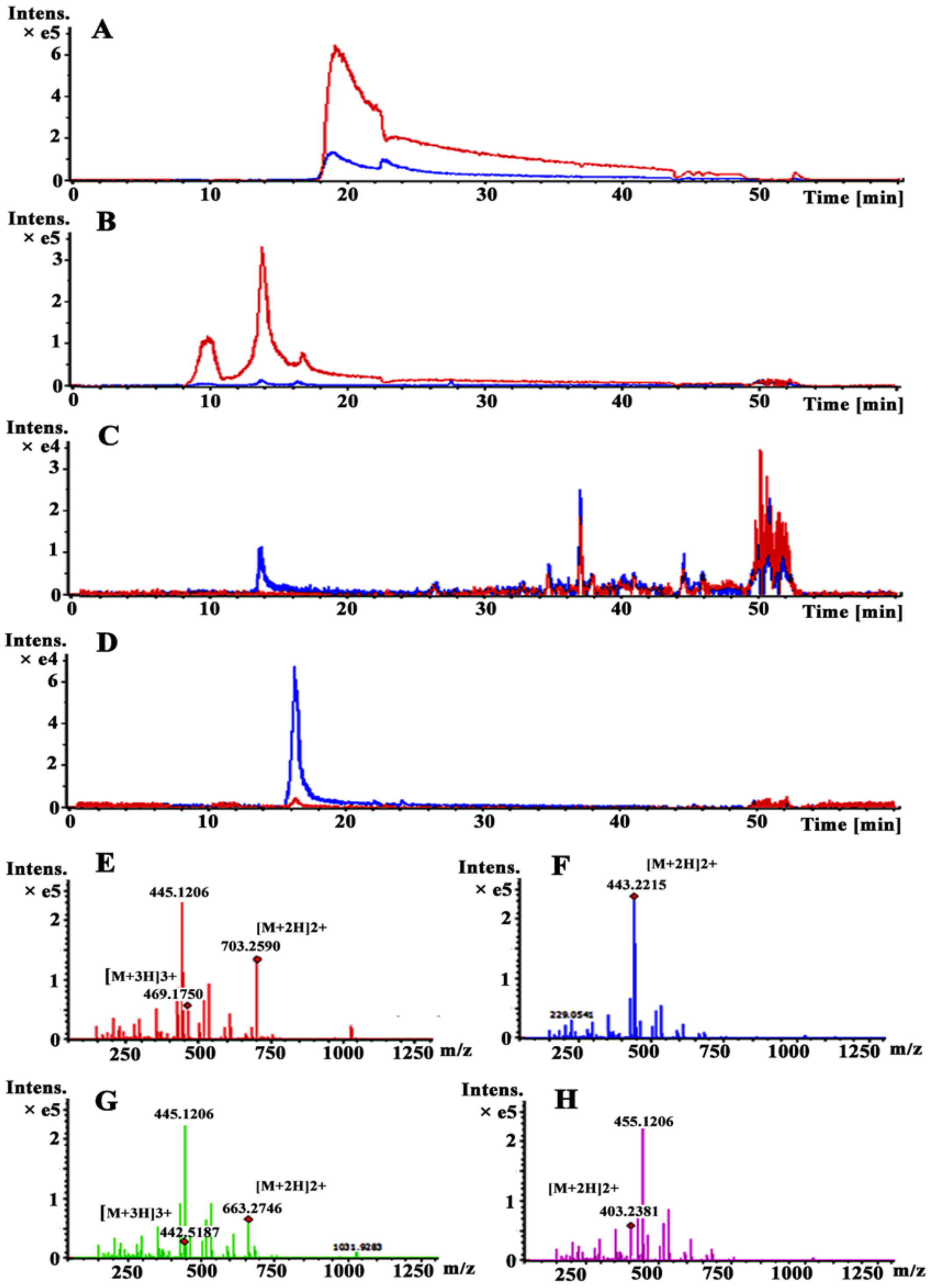

Figure S4. Extracted ion chromatogram of the peptides (A) DE-11p, (B) LY-6p, (C) LY-6, (D) DE-11, before (blue line) and after (red line) TiO_2 beads enrichment; Mass spectrum of the peptides (E) DE-11p, (F) LY-6p, (G) DE-11, and (H) LY-6 by nano liquid chromatography-maxis impact Q-TOF/MS system in positive scan model.

Considering the concentration of phosphorylated peptides will far lower than that of nonphosphorylated peptides in the peptide samples from MCF-7 breast cancer cells, we used

mixed peptide samples consisted of LY-6 (1 μg/mL) , DE-11 (1 μg/mL), LY-6p (0.1 g/mL) and DE-11p (0.1 μg/mL) as test sample to evaluate enrichment efficiency of commercial TiO_2 beads for phosphopeptide when high concentration nonphosphorylated peptides were as background. The results demonstrated that the mass response intensity of phosphopeptides DE-11p and LY-6p increased 6.5 times and 13.6 times, respectively, and nonphosphorylated peptides DE-11 and LY-6 almost could not be detected in the elution buffer after TiO2 beads enrichment. These results further demonstrated that the commercial TiO_2 beads can enrich phosphorylated peptides efficiently.
